# The Design and Simulation of a 16-Sensors Plantar Pressure Insole Layout for Different Applications: From Sports to Clinics, a Pilot Study

**DOI:** 10.3390/s21041450

**Published:** 2021-02-19

**Authors:** Alfredo Ciniglio, Annamaria Guiotto, Fabiola Spolaor, Zimi Sawacha

**Affiliations:** 1 Department of Information Engineering, DEI, University of Padova, 35131 Padova, Italy; ciniglioal@dei.unipd.it (A.C.); guiotto@dei.unipd.it (A.G.); fabiola.spolaor@unipd.it (F.S.); 2 Department of Medicine, DIMED, University of Padova, 35131 Padova, Italy

**Keywords:** plantar pressure insoles, layout, gait analysis, drop landing, weight lifting

## Abstract

The quantification of plantar pressure distribution is widely done in the diagnosis of lower limbs deformities, gait analysis, footwear design, and sport applications. To date, a number of pressure insole layouts have been proposed, with different configurations according to their applications. The goal of this study is to assess the validity of a 16-sensors (1.5 × 1.5 cm) pressure insole to detect plantar pressure distribution during different tasks in the clinic and sport domains. The data of 39 healthy adults, acquired with a Pedar-X^®^ system (Novel GmbH, Munich, Germany) during walking, weight lifting, and drop landing, were used to simulate the insole. The sensors were distributed by considering the location of the peak pressure on all trials: 4 on the hindfoot, 3 on the midfoot, and 9 on the forefoot. The following variables were computed with both systems and compared by estimating the Root Mean Square Error (RMSE): Peak/Mean Pressure, Ground Reaction Force (GRF), Center of Pressure (COP), the distance between COP and the origin, the Contact Area. The lowest (0.61%) and highest (82.4%) RMSE values were detected during gait on the medial-lateral COP and the GRF, respectively. This approach could be used for testing different layouts on various applications prior to production.

## 1. Introduction

Plantar pressure occurs on foot skin during daily activities and it represents the first variable used to conceive and validate footwear design [1]. The information derived from plantar pressure is critical not only for footwear design, but also in gait and posture research, for diagnosing lower extremity diseases or balance disorders, in injury prevention in sports, and other biomechanical applications [1]. Early studies mainly focused on foot deformities or foot diseases (e.g., normal gait, toe in, toe out, over supination, and heel walking gait abnormalities) and in pathological foot evaluation [2,3,4,5]; however, from 1985 onwards, researchers started to apply plantar pressure knowledge into ergonomics, sports, and footwear industries, in line with advanced technology growth [6]. In 1993, Frederick and Hartner optimized sports performance with thin-film pressure sensors and relatively inexpensive data acquisition hardware [7]. In 1999, Mueller et al. [4] drafted a guideline for the application of plantar pressure assessment in the evaluation and design of footwear for people without impairments [8,9]. Since 2000, an increased number of research has reported on athletic plantar pressure analysis with the aim to improve sports achievements [10,11]. In 2008, Morris et al. [12] analysed clinical gait analysis and investigated the pattern of walking by means of a custom shoe-integrated sensor system for wireless gait analysis and real-time feedback, in which spatial pressure distribution of the foot was used in pattern recognition and numerical analysis. The data available in the literature confirms the ever-growing interest in the measurement and interpretation of plantar pressure data, both in pathological and healthy subjects [13] (i.e., diabetes, foot injuries, foot surgery, footwear and insole etc.) [2,3,4,5,6,7,8,9,10,11,12,13].

In order to relate to different application requirements, a variety of plantar pressure measurement systems have been developed, divided mainly into two kinds: platforms system and in-shoe systems [1]. In particular, recently, the state of the art showed a huge interest in the design of low-cost, wearable plantar pressure devices to allow measurement in unrestricted environments where continuous monitoring during daily-life conditions for long periods of time are needed [14].

In this context, various pressure insoles are available in the market or have been developed in some laboratories, differing in size, sensor number, sensor type, and sensor layout, and consequently for their response to loading and accuracy [15]. The commercially available insoles span from 960 to 24 sensors and adopt different technologies: resistive sensors (960 sensors Tekscan F-Scan, Tekscan Inc., South Boston, MA, USA), capacitive sensors (230 sensors Xsensors, XSensor^®^ Technology Corporation, Calgary, AB, Canada, 85–99 sensors Novel Pedar-X^®^, Novel gmbh, Munich, Germany, 13 sensors Moticon OpenGo, Moticon ReGo AG, Munchen, Germany), and piezoresistive sensors (24 Parotec, Paromed GmbH, Neubeuern, Germany) [1,16]. Furthermore both in Zizoua et al. [17] and De Rossi et al. [18], we can find two examples of plantar pressure insoles with a large number of sensors—954 resistive sensors for medical diagnostic and 64 optoelectronic sensors for gait analysis applications.

It has been reported that the ideal plantar pressure device should be mobile, cable-free, placed in the sole of the shoe, and capable of effectively measuring outside the clinical or laboratory context [19,20]. In the context of gait analysis applications in a free living environment, a low number of sensors is preferrable. For instance, Lin et al. in 2016 [14] presented a low-cost sensor array including 48 pressure sensors, a 3-axis accelerometer, a 3-axis gyroscope, and a 3-axis magnetometer, while Aqueveque et al. [21] and Shu et al. [22] described a plantar pressure insole device with a reduced number of sensors (8 and 6 sensors, respectively). Over the last years, there has been an increasing interest in not only developing in-shoe foot plantar pressure insoles, but also instrumented socks or other textile applications [23,24,25,26,27]. Esfahani et al. [23] successfully assessed the accuracy of a system composed of instrumented socks (3 sensors) and smart shirts in classifying different human activities (i.e., simulated occupational tasks, normal and abnormal walking patterns, and several typical daily activities). In Preece et al. [25], instrumented socks with one resistive sensor were developed to provide the automatic identification of gait events. Similar devices can be found in Tirosh et al. [26] and Oks et al. [27] with 3 or 5 sensors for gait analysis purposes, respectively. Some are suitable for specific tasks, some for clinical purposes, some for sport performance and injury prevention, and some are designed for gait phases detection only; however, their validity and repeatability influence their appropriateness for specific tasks in both clinical and research settings [15]. All these results reported in Aqueveque 2020 [21] highlight that different gait measurement methods have been developed in order to identify parameters that can contribute to gait cycles.

Even though these publications reported solutions with a lower number of sensors, some authors have claimed that the sole of foot can be divided into 15 areas that cover most body weight changes [22], and should allow minor loss of information with respect to the platform system [1,2,3,4,5,6,7,8,9,10,11,12,13,14,15,16,17,18,19,20,21,22].

The aim of the present contribution is to assess the validity of a 16-sensors (1.5 × 1.5 cm) pressure insole in detecting plantar pressure distribution during different tasks, both in the clinic and sport domains. In defining the layout, the spatial location of the peak plantar pressure from a dataset of healthy subjects, performing tasks from clinical to sport applications, was considered. Furthermore, the five main regions of interest in the foot, according to the common methods generally applied for masking the footprint, were taken into account [28]: medial and lateral hindfoot, midfoot, medial and lateral forefoot, also including the toes. The data acquired with a Novel Pedar-X^®^ system (99 sensors, 100 Hz) were used to simulate the 16-sensors layout and the reliability of its measures was assessed by comparing the following variables estimated with both systems: Peak and Mean Pressure, Ground Reaction Force (GRF), Center of Pressure (COP), the distance between COP and the origin, the Contact Area. According to Price et al. (2016) [15], Pedar-X^®^ could be considered a gold standard for in-shoe plantar pressure measures as it reveals the greatest accuracy and repeatability compared to the other three state of the art devices. The results of this study may therefore provide insights on which plantar pressure variables are the most affected from the reduction in sensor number and the increase in sensor size. This information could be useful in planning plantar insole devices that are suitable for a wide variety of applications through a low number of sensors.

## 2. Material and Methods

### 2.1. Subjects

The data of 39 healthy subjects were retrospectively selected from the database of plantar pressure data available at the BiomovLab (Department of Information Engineering of the University of Padova). Anonymized data were available and each subject was associated with a numerical code. Inclusion criteria were: healthy subjects with no record of orthopedics or neurologic disease, at least 3 trials per subject available, plantar pressure data from plantar pressure insoles acquired with the same device. The data of 3 different cohorts of subjects were extracted from the database as follows: 10 subjects divided into 7 males and 3 females who performed at least ten steps while walking on a flat 10 m walkway; 11 subjects divided into 9 males and 2 females who performed 6 single leg drops landing from a 32-cm height [29]; 18 subjects divided into 8 males and 10 females, who performed 3 consecutive squat lifts carrying, respectively, 16 kg and 8 kg from the floor to 73 cm height (according to the NIOSH—UNI EN 1005-27) [30]. The demographic data of each group of subjects is reported in Table 1.

### 2.2. Instrumental Protocol

All the data were acquired at the BiomovLab of the Department of Information Engineering of the University of Padova between the years 2005 and 2019, through a stereophotogrammetric system (BTS, 6 cameras, 60 Hz) synchronized with a 3D force platform (Bertec FP2060, 960 Hz) and plantar pressure insoles (Pedar-X^®^, Novel, 100 Hz). The following insoles sizes were used: 38–39, 40–41, 42–43, 44–45. On each subject, reflective markers were applied according to a modified version of Leardini et al., 2007 [31], as in Sawacha et al., 2009 [32]. Before the beginning of each acquisition session, the zeroing process was performed following the guidelines of the Novel Pedar manual as follows: a message will appear to unload the left insole (i.e., have the subject lift their left foot slightly off the ground), after a message will appear to unload the right insole. After the zeroing process, the subject is ready to perform the task.

In the current study the temporal frames of each task were defined by combining the force plate data, plantar pressure data, and the trajectories of the 5th lumbar vertebra marker. The right and left heel markers are as follows:

- gait: initial contact was detected as the first instant when the heel pressure signal exceeds its threshold (defined during the zeroing process); the toe-off event was detected as the first instant when the hallux pressure signal goes below its threshold. The heel marker trajectory was also used in order to confirm the step detection.
- drop: the subject stood in a balanced position near the front edge of the 32 cm platform with the foot of the testing leg completely off the platform and suspended over the floor, with the heel of that foot resting against the front of the platform. This placed the subject’s center of mass as far forward as possible in an attempt to limit horizontal motion. The subject’s weight was supported fully on the platform by the non-testing leg. To initiate movement, the subject weight-shifted forward and dropped vertically, while attempting to land in a balanced position on the testing leg. Subjects were instructed to “fall” from the platform without jumping or lowering their body prior to leaving the platform [26]. The beginning of the task was detected as the first instant when the hallux pressure signal goes below the threshold (of the last leg in contact with the platform). The end of the task was detected two seconds after landing. The heel marker trajectory together with the force plate was also used in order to confirm the task detection.
- lifting: Each subject performed 3 consecutive squat lifts carrying 16 kg for the male cohort and 8 kg for the female cohort, respectively, from the floor to 73 cm height. The task was divided into five phases:
  ○ Unloaded descending (UD): the subject takes the weight that is placed on the floor;
  ○ Loaded ascending (LA): the subject grabs the weight and lifts it onto the support;
  ○ Leave and peak: the subject places the weight on the support and grabs it again to start the next phase (this phase was not analyzed);
  ○ Loaded descending (LD): the subject carries the weight to its initial position;
  ○ Unloaded ascending (UA): the subject returns to its initial position.

The temporal frames of each phase were detected by considering the 5th lumbar vertebra marker trajectory.

### 2.3. Simulated Sensor Layouts

**Figure 1:**
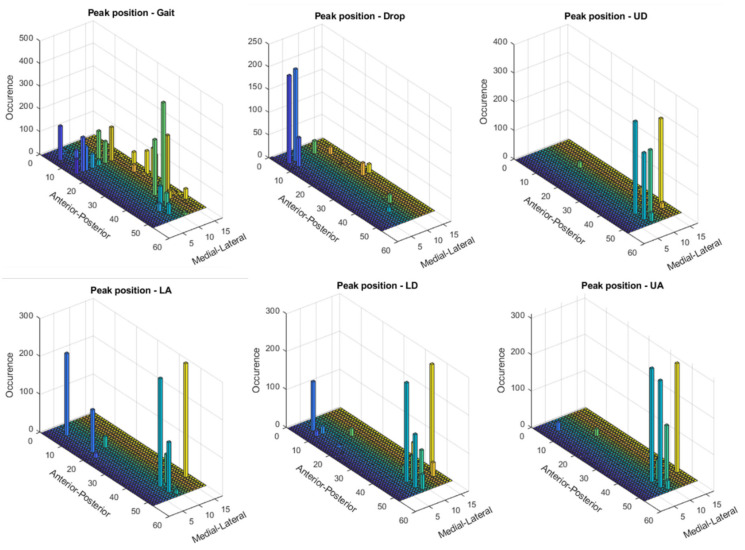
Each bar in the bar plot represents the resultant location of the Peak Pressure on the Novel Pedar-X^®^ insole sensors by considering all the available trials for each task. On the *y*-axis, the longitudinal axis of the insole is represented (anterior-posterior axis), while on the *x*-axis, the medial-lateral axis is represented. On the *z*-axis, the number of times that the Peak Pressure occurs on each sensor is represented.

**Figure 2:**
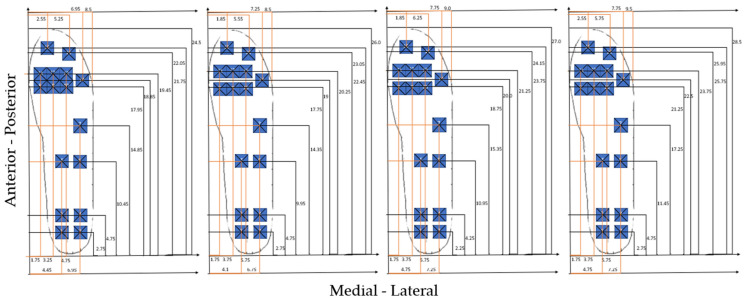
Simulated layout of insoles size, respectively from left to right, 38–39, 40–41, 42–43, and 44–45. The location of each sensor is reported in cm. On the *x*-axis, the medial lateral axis is represented, while on the *y*-axis, the anterior-posterior axis of the insole is represented.

Experimental data acquired with Novel insoles (99 sensors, 5 × 5 mm) were exported from the Novel EmedLink software and imported in Matlab (2018b). By considering that 15 sensors were reported to cover most of the body weight changes [22], the layout proposed in Shu et al. [22] was updated by including a further sensor in correspondence to the medial aspect of the midfoot, in order to enable the detection of the foot type (i.e., flat foot, cavus foot, normal foot). Hence, a 16-sensors (1.5 × 1.5 cm) insole was extracted from the Novel sensors map. A larger sensor size was chosen in an attempt to ensure full coverage of the five main regions of interest in the foot, according to the two common methods generally applied for masking the footprint (i.e., manual and automated masking): medial and lateral hindfoot, midfoot, medial and lateral forefoot, also including the toes [28]. In defining the sensor layout on the plantar aspect of the foot, the position of the center of each sensor in the Novel insole, the desired sensors number, their shape, their size, and the empty space between sensors were taken into account, as in Shu [22]. The original dataset was then analyzed and the sensors where more often the peak of pressure was detected by considering all trials, all tasks, and all insole sizes were chosen (see Figure 1). Hence, three sensors from the Novel Pedar-X^®^ Insole were grouped together and the following layout was obtained (see Figure 2): 4 sensors on the hindfoot (2 on the medial and two on the lateral aspect) to capture the plantar pressure distribution during both the heel contact and the loading response; 2 on the lateral and 1 on the medial aspect of the midfoot, to enable the classification of different foot types such as cavus and flat foot; 7 on the forefoot (to cover both medial and lateral forefoot) and 2 on the toes (forefoot), to capture the push off phase of gait. The 15 × 15 mm sensor was conceived trying to find the best compromise between covering the regions of interest and trying to limit the size of the empty spaces between sensors, which could also produce loss of information. In order to compare the data of the simulated insole and the experimental dataset, the experimental sensors closer to each simulated sensor were grouped together (see Figure 2) and their data averaged. A different layout was generated for each foot size.

### 2.4. Data Analysis

The most relevant parameters that can be calculated using the plantar pressure insoles were considered: Peak and Mean Pressure, the vertical component of the Ground Reaction Force (GRF), Center of Pressure (COP), the distance between COP and the origin (dCOP), and Contact Area. All data, except for the COP, were filtered using a 3rd order lowpass Butterworth filter with a cutoff frequency of 1/8 of the sampling frequency [33].

In this study, two different approaches were adopted to calculate the COP; in the first formulation, according to [22], the following equations were applied:

(1) $$X_{\mathrm{cop}}\;=\;\frac{\sum_{i=1}^{n}X_{i}V_{i}}{\sum_{i=1}^{n}V_{i}}\;Y_{\mathrm{cop}}\;=\;\frac{\sum_{i=1}^{n}Y_{i}V_{i}}{\sum_{i=1}^{n}V_{i}}$$

*n* denotes the total number of sensors, *i* denotes a certain sensor, X and Y are the coordinates of the whole foot shape area, and *V_i_* is the value of the *i*-th sensor.

In the second formulation, according to [21], the COP was calculated, using the weighted average:

(2) $$X_{\mathrm{cop}2}\;=\;\frac{\sum_{i=1}^{n}X_{cop,i}p_{i}}{\sum_{i=1}^{n}p_{i}}\;Y_{\mathrm{cop}2}\;=\;\frac{\sum_{i=1}^{n}Y_{cop,i}p_{i}}{\sum_{i=1}^{n}p_{i}}$$

*p_i_* is the weight of the *i*-th sensor, calculated by normalizing the pressure value of *i*-th sensor, with the Peak Pressure of the *i*-th sensor.

In order to compare the simulated layout (16 sensors) with the original dataset (Novel Pedar), the Root Mean Square Error (RMSE) was calculated between each variable in percentage of the gold standard value (i.e., Novel):

(3) $$RMSE\%=\;\frac{\sqrt{\sum_{i=1}^{n}{\left(a-\alpha \right)}^{2}}}{Experimental\;Peak}*100\%$$

*a* is the observed values (i.e., Novel), and α is the expected values.

Left and right insoles values were averaged.

## 3. Results

**Figure 3:**
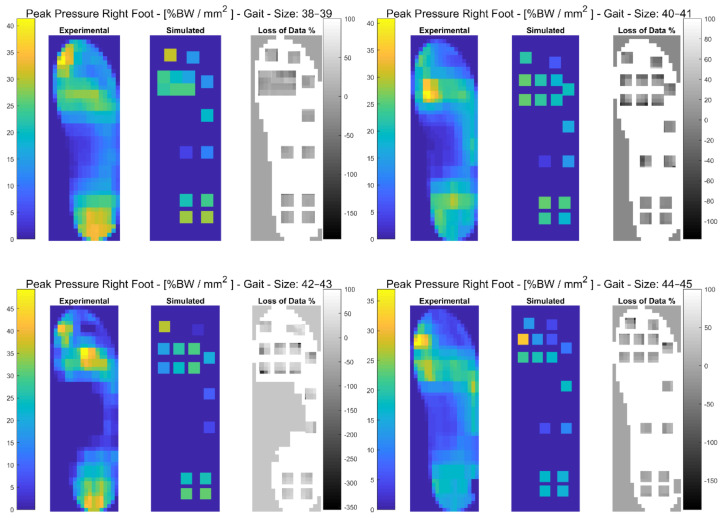
The Peak Pressure of the right foot for each insole size in %BW/mm^2^ during gait is represented: from left to right the Experimental (Pedar-X^®^ system), simulated (prototype layout), and loss of data% footprints were reported. Experimental and Simulated data were color-coded: yellow revealed the highest pressure, blue the lowest. Loss of data% was grey-level coded: white revealed the percentage of the data that was not detected by the simulated layout, from grey to black, the percentage of the data that was estimated by the simulated layout was indicated.

**Figure 4:**
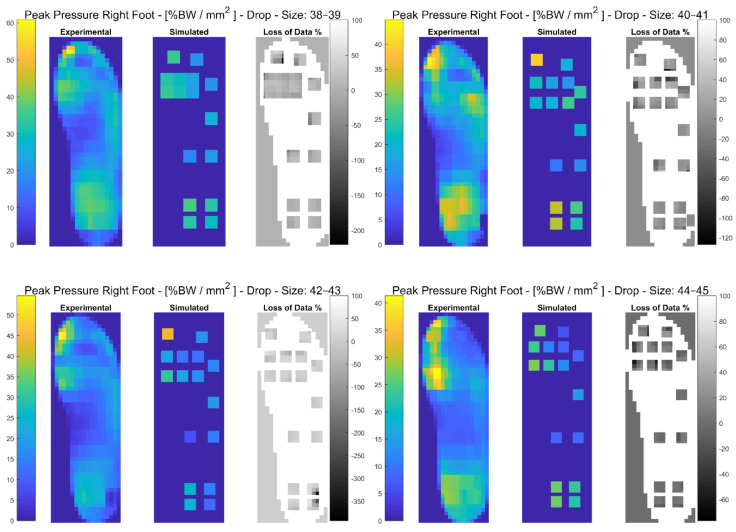
The Peak Pressure of right foot for each insole size in %BW/mm^2^ during drop landing is represented: from left to right the Experimental (Pedar-X^®^ system), simulated (prototype layout), and loss of data% footprints were reported. Experimental and Simulated data were color-coded: yellow revealed the highest pressure, blue the lowest. Loss of data% was grey-level coded: white revealed the percentage of the data that was not detected by the simulated layout, from grey to black the percentage of the data that was estimated by the simulated layout was indicated.

**Figure 5:**
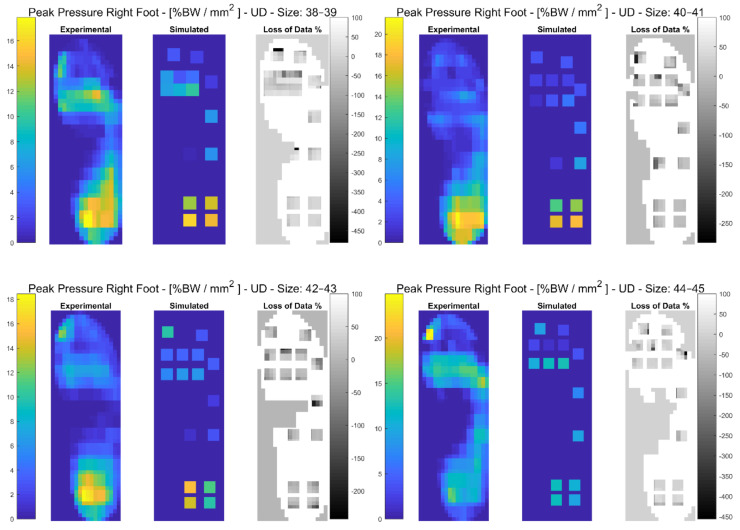
The Peak Pressure of right foot for each insole size in %BW/mm^2^ during unloaded descending is represented: from left to right the Experimental (Pedar-X^®^ system), simulated (prototype layout), and loss of data% footprints were reported. Experimental and simulated data were color-coded: yellow revealed the highest pressure, blue the lowest. Loss of data% was grey-level coded: white revealed the percentage of the data that was not detected by the simulated layout, from grey to black, the percentage of the data that was estimated by the simulated layout was indicated.

**Figure 6:**
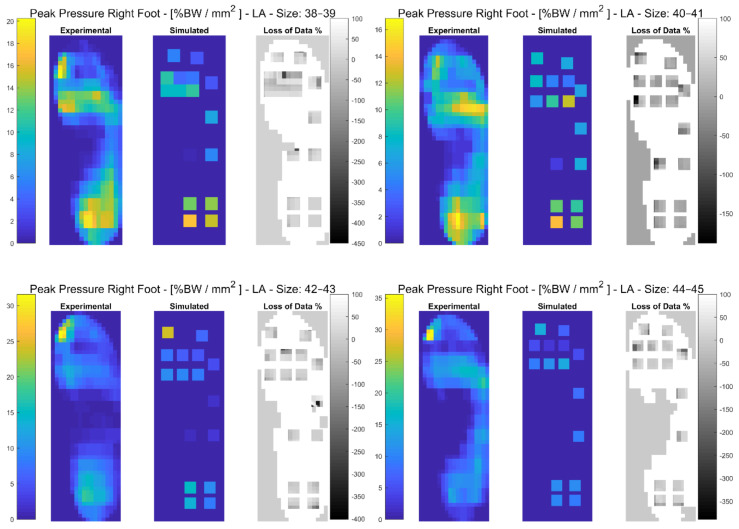
The Peak Pressure of right foot for each insole size in %BW/mm^2^ during loaded ascending is represented: from left to right the Experimental (Pedar-X^®^ system), simulated (prototype layout), and loss of data% footprints were reported. Experimental and Simulated data were color-coded: yellow revealed the highest pressure, blue the lowest. Loss of data% was grey-level coded: white revealed the percentage of the data that was not detected by the simulated layout, from grey to black the percentage of the data that was estimated by the simulated layout was indicated.

**Figure 7:**
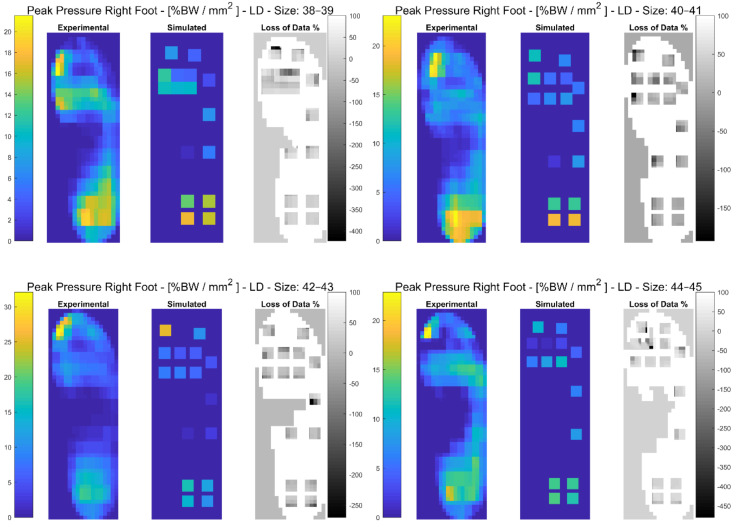
The Peak Pressure of right foot for each insole size in %BW/mm^2^ during loaded descending is represented: from left to right the Experimental (Pedar-X^®^ system), simulated (prototype layout), and loss of data% footprints were reported. Experimental and Simulated data were color-coded: yellow revealed the highest pressure, blue the lowest. Loss of data% was grey-level coded: white revealed the percentage of the data that was not detected by the simulated layout, from grey to black, the percentage of the data that was estimated by the simulated layout was indicated.

**Figure 8:**
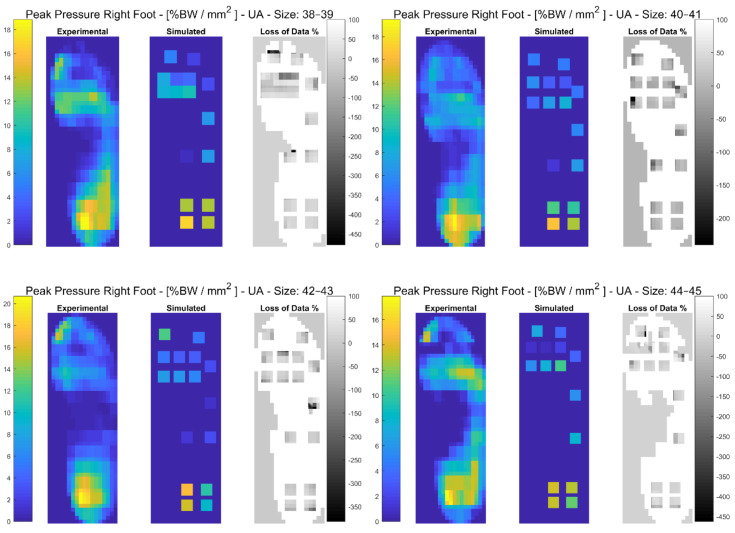
The Peak Pressure of the right foot for each insole size in %BW/mm^2^ during unloaded ascending is represented: from left to right the Experimental (Pedar-X^®^ system), simulated (prototype layout), and loss of data% footprints were reported. Experimental and simulated data were color-coded: yellow revealed the highest pressure, blue the lowest. Loss of data% was grey-level coded: white revealed the percentage of the data that was not detected by the simulated layout, from grey to black, the percentage of the data that was estimated by the simulated layout was indicated.

In the following paragraphs, the results were reported for each task, for each computed variable on each insole size in terms of maximum and minimum RMSE values. The loss of data was also computed from the pressure maps according to the following equation (reported in the Figure 3, Figure 4, Figure 5, Figure 6, Figure 7 and Figure 8):

(4) $$Loss\;of\;Data\;\%=\;\frac{Experimental\;Value-Simulated}{Experimental\;Value}*100\%\;$$

### 3.1. Gait

The results showed that the minimum RMSE value of 0.61% was registered on the Medial-Lateral COP displacement for the insole size 42–43 and the maximum RMSE values of 82.45% and 82.40% were registered on the Mean Pressure and on the GRF, respectively, for insole size 44–45. A detailed description of both the variables (mean (SD)) and the RMSE ranges for each insole size, can be found in Table 2. In Figure 3, and in Supplementary Materials (Figures S1–S14), the plantar pressure distribution in the Pedar-X^®^ system, the simulated layout, and the loss of data% were represented. Furthermore, in Figures S1–S14, the temporal distribution of Peak Pressure, Mean Pressure, GRF, dCOP, Medial-Lateral and Anterior-Posterior COP, Contact Area, as well as RMSE (calculated on each variable in percentage of the corresponding value in the gold standard Pedar-X^®^) were reported for each insole size.

### 3.2. Drop Landing

The results showed that the minimum RMSE value of 0.93% was registered on the dCOP for insole size 40–41 and maximum RMSE of 72.12% was registered on the GRF for insole size 44–45. A detailed description of both the variables (mean (SD)) and the RMSE ranges for each insole size can be found in Table 3. In Figure 4, and in Figures S15–S28, the plantar pressure distribution in the Pedar-X^®^ system, the simulated layout, and the loss of data% (see Equation (4)) are represented. Furthermore, in Figures S15–S28, the temporal distribution of Peak Pressure, Mean Pressure, GRF, dCOP, Medial-Lateral and Anterior-Posterior COP, Contact Area, as well as RMSE (calculated on each variable in percentage of the corresponding value in the gold standard Pedar-X^®^), were reported for each insole size.

### 3.3. Weight Lifting

#### 3.3.1. Unloaded Descending

Results showed that the minimum RMSE value of 1.04% was registered on the Anterior-Posterior COP for insole size 44–45 and maximum RMSE value of 70.40% was registered on the GRF for insole size 44–45. A detailed description of both the variables (mean(SD)) and the RMSE ranges for each insole size can be found in Table 4. In Figure 5, and in Figures S29–S42, the plantar pressure distribution in the Pedar-X^®^ system, the simulated layout, and the loss of data% are represented. Furthermore, in Figures S29–S42, the temporal distribution of Peak Pressure, Mean Pressure, GRF, dCOP, Medial-Lateral and Anterior-Posterior COP, Contact Area, as well as RMSE (calculated on each variable in percentage of the corresponding value in the gold standard Pedar-X^®^) were reported for each insole size.

#### 3.3.2. Loaded Ascending

The results showed that the minimum RMSE value of 2.21% was registered on the Anterior-Posterior COP for insole size 38–39 and maximum RMSE value of 70.87% was registered on Mean Pressure for insole size 44–45. A detailed description of both the variables (mean(SD)) and the RMSE ranges for each insole size can be found in Table 5. In Figure 6 and in Figures S43–S56, the plantar pressure distribution in the Pedar-X^®^ system, the simulated layout, and the loss of data% were represented. Furthermore, in Figures S43–S56, the temporal distribution of the Peak Pressure, the Mean Pressure, the GRF, dCOP, Medial-Lateral and Anterior-Posterior COP, Contact Area, as well as RMSE (calculated on each variable in terms of percentage of the corresponding value in the gold standard Pedar-X^®^) were reported for each insole size.

#### 3.3.3. Loaded Descending

Results showed that RMSE values ranged between 1.94% for the Anterior-Posterior COP for size 44–45 and 72.17% for the Mean Pressure for size 44–45. A detailed description of both the variables (mean(SD)) and the RMSE ranges for each insole size, can be found in Table 6. In Figure 7 and in Figures S56–S70 the plantar pressure distribution in the Pedar-X^®^ system, the simulated layout, and the loss of data% were represented. Furthermore in Figures S56–S70 the temporal distribution of the Peak Pressure, the Mean Pressure, the GRF, dCOP, Medial-Lateral and Anterior-Posterior COP, Contact Area as well as the RMSE (calculated on each variable in percentage of the corresponding value in the gold standard Pedar-X^®^) were reported for each insole size.

#### 3.3.4. Unloaded Ascending

The results showed that the minimum RMSE value of 1.04% was registered on the Anterior-Posterior COP for insole size 44–45 and maximum RMSE value of 70.39% was registered on the Mean Pressure for insole size 44–45. A detailed description of both the variables (mean(SD)) and the RMSE ranges for each insole size, can be found in Table 7. In Figure 8 and in Figures S71–S84, the plantar pressure distribution in the Pedar-X^®^ system, the simulated layout, and the loss of data% were represented. Furthermore in Figures S71–S84, the temporal distribution of Peak Pressure, Mean Pressure, GRF, dCOP, Medial-Lateral and Anterior-Posterior COP, Contact Area, as well as RMSE (calculated on each variable in percentage of the corresponding value in the gold standard Pedar-X^®^) were reported for each insole size.

## 4. Discussion

The current study focused on the assessment of the feasibility of adopting a plantar pressure insole with a low number of sensors in order to monitor plantar pressure distribution across different tasks spanning from clinical to sport and work-related injury risk applications. For this purpose, a 16-sensors layout was simulated and applied to a dataset including self-selected speed walking trials, drop landing, and weight lifting tasks. Notably, differing from previous studies, the sensor layout was defined by taking into account the spatial location of the peak pressure acquired during different tasks spanning from sport to clinical applications. Furthermore, both number and sensor size were defined as a compromise between taking into account the peak pressure location on the plantar aspect of the foot, and by ensuring the coverage of the most common regions of interest, according to state-of-the-art masking procedures [28]. Indeed, clinical applications generally apply a mask to the footprint, and sub-divide it into regions of interest, in order to analyze plantar pressure parameters accordingly [28]. This has been recognized to provide more descriptive and clinically relevant information than when examining the foot as a whole [28]. Two masking techniques are commonly adopted (manual and automated) and five main regions of interest are usually identified (medial and lateral hindfoot, midfoot, medial and lateral forefoot, including the toes) [28]. Based on these considerations, a 16-sensors layout ensuring sufficient contact area in these five regions was selected, thus assuring the assessment of plantar pressure variables during the different phases of each analyzed task [16,18].

Differently from majority of the studies (see Table 8), the results of the simulations were compared with the original dataset acquired by means of the Novel Pedar-X^®^ system with respect to the most common plantar pressure variables [1,2,3,4,7,8,11,13,15]. Compressively, the presence of differences on each of the observed variables, regardless of the task and insole size, was revealed. This finds agreement with the results of Stöggl and Martiner [16] who compared a 13-sensor plantar insole device with the Novel Pedar-X^®^ system across multiple tasks (i.e., walking, running, jumping) and concluded that the system with the lower number of sensors underestimates the GRF (see Table 9). In particular, the RMSE% calculated in the GRF during self-selected speed walking, presented in this study (RMSE% = 32 ÷ 82 in the 40–41 and 44–45 insole sizes, respectively), was comparable to the one extracted from the dataset presented in Stoggl and Martiner [16] during slow and fast walking (RMSE% = 47.9 (fast) ÷ 76.4 (slow)). When considering a task with higher contact forces and shorter contact time, such as drop landing, we reported higher RMSE% values in the GRF (RMSE% = 31.8 ÷ 72.25 in our study vs. RMSE% = 35.1 ÷ 47.5 in [16]). However, the evaluated tasks were slightly different, as in their case a drop jump test was analyzed. In this respect, the authors observed that in their study, differences diminished when ground contact times were longer and forces lower (i.e., walking). This observation was not confirmed by our results when considering that the highest RMSE% on the GRF (82%) was observed during walking and on Peak Pressure (42.32%) during the loaded ascending phase of the weight lifting task, in both cases in the largest insoles size (44–45). The results on the GRF suggest that the larger the insole size, the lower the accuracy of the measures. However, this was not confirmed by other variables such as Peak and Mean Pressure or COP. Overall, the task that was most affected by the sensors’ reduction in terms of GRF can be considered the self-selected speed walking. However, our results did not give a clear indication of a least-affected task, as both gait and drop landing tasks recorded a minimum RMSE% of 32% of the Novel Pedar-X^®^ system’s value.

When considering specific tasks, we can make the following considerations.

### 4.1. Walking

The best RMSE scores for the estimates of GRF and Peak Pressure, respectively, of 32% (40–41 insole size) and 6% (44–45 insole size), were detected in this task. However, for the same task, the maximum RMSE% of 82% (44–45 insole size) was estimated for both GRF and Mean Pressure. While for the Contact Area, the RMSE% ranged between 43% (38–39 insole size) and 83% (44–45 insole size); for both the Anterior-Posterior and Medial-Lateral COP excursions, very low RMSE% was revealed, and in particular the best one was recorded in the Anterior-Posterior direction (0.6% RMSE%, insole size 42–43).

### 4.2. Drop Landing

When considering GRF, RMSE% values ranged between 32% (42–43 insole size) and 72% (44–45 insole size). In terms of Peak Pressure, RMSE% ranged between 21% (38–39 insole size) and 40% (40–41 insole size), while for Mean Pressure estimates, it ranged between 29% (42–43 insole size) and 72% (44–45 insole size). Very high RMSE% values were reported for the Contact Area that ranged between 53% (38–39 insole size) and 82% (44–45 insole size), thus showing a high impact of sensors reduction on the assessment of this variable during tasks characterized by high contact forces applied during short contact time. In agreement with the other tasks, COP excursion recorded the lowest RMSE% values ranging between 2% in the Anterior-Posterior excursion (40–41 insole size) and 8% in both Medial-Lateral (42–43 insole size) and Anterior-Posterior (44–45 insole size) excursions.

### 4.3. Weight Lifting

When considering both GRF and Mean Pressure estimates, a maximum RMSE value of 72% was reported during the loaded descending phase (44–45 insole size). For the same variables, the lowest RMSE% value of 43% (38–39 insole size) was detected during the unloaded descending phase. Peak Pressure instead reported the maximum RMSE% value of 45% during the unloaded descending phase (44–45 insole size), and the minimum value of 11% during both the unloaded descending and the loaded ascending (42–43 insole size) phases. In terms of Contact Area, the maximum RMSE% value of 76% was reported during both the unloaded ascending and the loaded descending phases (44–45 insole size). In addition, in this case, the COP excursion in the medial-lateral direction reported the lowest RMSE% values ranging between 1% (44–45 insole size) during the unloaded ascending phase and 12% (44–45 insole) during the loaded ascending phase.

Compressively, the results of the current study showed that RMSE% for estimates from Mean Pressure and GRF reported the highest values, when compared with the other variables. This was found regardless of the task analyzed. However, consistently across all variables, a strong association between the larger insole size and the lower accuracy of the measures was observed. Noticeably these results clearly indicated that COP measures could be assessed with good precision, regardless of sensors reduction and insole size.

When considering our results in light of plantar pressure monitoring applications, the impact of an underestimate in Peak Pressure should be considered more relevant in the prediction of diabetic ulceration, where subjects at risk are identified based on the site of high plantar pressure. The latter is generally expressed as a Peak Pressure level or pressure-time integral [34] or as Peak Pressure values on sites of previous ulceration (in case of ulcer recurrence). Several studies have showed that in diabetic plantar ulcer prevention, techniques that provide an effective degree of off-loading play an important role in plantar foot ulcer healing [35]. Within this context, a large effort has been made in defining the threshold for off-loading, which is required to adequately heal neuropathic foot ulcers [34]. Nevertheless, in planning foot orthoses, the variables more often considered in the attempt to decrease excessive plantar pressures from specific sites of actual or potential ulceration, were Peak Pressure, duration of loading, rate of loading, pressure/time integral, and total contact surface area. People with diabetic neuropathy may develop rigid feet as a result of muscle alterations [36,37], joint stiffness, and thickening of tendons and ligaments [38], with consequent altered Peak Pressure, GRF, and Contact Area [36,39], thus showing the importance of assessing with high accuracy not only the Peak or Mean Pressure, but also Contact Area and GRF.

In foot disorders, in general, higher pressure is associated with foot alterations compared to subjects with normal feet [6], and in Xiong et al. [40], a foot pressure pain threshold was determined, thus showing the crucial role played by the reliability of pressure measures (mean and peak) in assessing foot disorders. When also considering other variables such as Contact Area and GRF, their role should not be neglected in the design of footwear and foot orthotics that target reducing higher foot contact forces by transferring the load to other regions [6]. Contact Area is also a variable of particular interest for people at risk of falls [41], as a lack of balance could be the result of a reduced tactile contact with the ground due to a reduced Contact Area, which weakens the reflex action of foot and leg muscles.

When moving to sports applications, pressure measuring devices have been identified as appropriate tools for injury prevention [42] or for coaching [6], and overall, the key variables considered are: plantar pressure, GRF and its location, Contact Area, timing, and stride parameters [42]. In the case of peak GRF, this was found to be associated with running injuries [42], and in particular some studies have focused on injuries located in specific foot areas such as metatarsal stress fractures or plantar fasciitis, thus showing the important role of precise assessment of the Contact Area [43,44].

Noteworthy mentions are COP measures applications, as COP has been identified as a measure of neuromuscular control during posture and gait. Tracking COP during the stance phase of gait can help identify possible foot pathologies, assess the effectiveness or help the design of foot orthotics, and allow balance control investigation during gait [45]. COP excursion in both anterior-posterior and medial-lateral directions is frequently used in clinical practice. The results of our study seem to confirm the possibility of assessing this variable with sufficient precision with a reduced sensors number, thus opening the possibility for real-life applications, such as ambient-assisted living and sports, which require flexibility, mobility, simplicity, and applicability in various environments [6,22].

The important limitations of the current study should not be neglected. First, only healthy subjects were involved, as compared to previous works that assessed the suitability of the proposed in-shoe plantar pressure device on pathological subjects as well [24].

Furthermore the size of the sensors chosen to simulate the 16-sensors layout is in contrast with the state-of-the-art guidelines for plantar pressure devices, which indicate to adopt a spatial resolution no larger than 6.2 mm [46], or 5 mm [47]. This is further supported by Lord’s model [48] and by Pataky [49], who generalized Lord’s model [48] for non-pathological feet and considered not only the metatarsal area. They showed that while with a 5-mm resolution the Peak Pressure can be estimated with an accuracy of 90% on the metatarsal heads, a 10-mm resolution can lead to a 30% underestimation [49]. Therefore, the larger the size of the sensors, the larger the underestimates could be on both Peak Pressure and GRF. However, it should be mentioned that these studies were conducted on plantar pressure platforms and no one took into consideration the extension of these results to plantar pressure insoles. Based on [1], in the case of in-shoe systems, a number of sensors equal to 15 should be considered adequate for the majority of applications in both sport and clinical fields, and the best solution should take into account both limiting sensor size but avoiding large empty spaces, which could also produce loss of information. However, insoles with larger sensors are commercially available and their use suggested for any application [16]. For instance, in comparison with an insole with the same number of sensors, our simulated insole presents a sensorized area of 3600 mm^2^ over a total surface of 15787 mm^2^ (considering an insole size of 38–39), while Moticon presents a sensorized area of 10109 mm^2^ over a total surface of 15787 mm^2^ (considering an insole size of 38–39).

State-of-the-art also reported solutions with a reduced number of sensors with larger size such as Wang et al., 2016 [50] and Shu et al., 2010 [22], where the entire foot sole was divided into 6 or 7 sensors, respectively, with 4 or 3 pressure-sensing cells in the metatarsal region, 1 pressure-sensing cell or none in the midfoot, and 3 pressure-sensing cells in the heel region. By considering that we adopted larger size sensors, our results find agreement with Stöggl and Martiner [16], who reported an underestimate ranging between 35.1% (RMSE%) and 76.4% (RMSE%) on GRF with a Moticon device characterized by 13 sensors. It should be mentioned that Figure 2, Figure 3, Figure 4, Figure 5, Figure 6, Figure 7 and Figure 8 showed that the results of the simulated layouts were mainly affected in terms of the amount of peak of pressure that was underestimated rather than its spatial location on the insole, in agreement with Wang 2016 [50], who compared the influence of different layouts, sizes, and number of pressure-sensing cells on COP coordinates’ estimation. Their results indicated that reliable COP estimation could be obtained with seven pressure-sensing cells of 2.0–2.5 cm, which represented the best compromise between simplifying the wearable system and obtaining precise information.

It should be further taken into account that the simulated insole was derived from a dataset captured by means of a capacitive sensors plantar pressure insole, and cannot be extended straightforward to an insole with a reduced number of resistive or piezoresistive sensors of similar size. In this respect, future developments should include the application of this methodology to a similar dataset obtained from resistive sensors insoles.

Furthermore, our study did not take into account the impact of a reduced number of sensors in detecting gait cycle phases, which was out of the scope of the current paper, but could be considered in the future.

Least but not less important, this study can benefit from the possibility to compare the results with measures obtained from a prototype with the same characteristics, in order to take into account other parameters that characterize sensor performance (linearity, hysteresis, pressure range, and temperature sensitivity) rather than only sensor size [1].

It should not be neglected that compared to plantar pressure insoles, smart socks have been recognized as a more flexible and mobile solution, with improved performance and efficiency in terms of power consumption and communication technology, with reduced costs. Furthermore, a better compliance was reported from the users [51,52,53,54,55]. However, to the author’s knowledge, no studies conducted with this technology (see Table 8) have assessed the validity of all the plantar pressure measures as the present contribution (i.e., Peak and Mean Pressure, vertical component of the Ground Reaction Force (GRF), Center of Pressure (COP), the distance between COP and the origin (dCOP), and Contact Area).

In conclusion, when high accuracy in the absolute values of the variables extracted from the plantar pressure measurement device is required, the Novel Pedar-X^®^ should be considered preferable, as the layout with a reduced number of sensors underestimated Mean and Peak Pressure, GRF, and Contact Area across all trials and tasks, regardless of insole size. It should be mentioned that the highest RMSE% values were recorded on simulation of the larger sizes of foot insoles, thus showing an association between insole size and accuracy of the measures. This aspect should be kept in mind when designing pressure insole devices with a constant sensors size and a low number of sensors. However, when limiting the analysis to COP displacement, the results indicate that the simulated configuration with 16 sensors has a good measurement performance with RMSE% ranging between 10.21% and 0.61%.

## Figures and Tables

**Table 1:** Demographic data of each group of subjects.

Task	Age [Years] Mean (SD)	Weight [Kg] Mean (SD)	Height [m] Mean (SD)	BMI [Kg/m^2^] Mean (SD)	Shoe Size Mean (SD)
Gait	28.3 (7.27)	68.7 (10.97)	1.72 (0.05)	23.19 (2.96)	40.30 (2.31)
Drop Landing	26.2 (3.33)	68.45 (7.71)	1.75 (0.07)	22.22 (1.33)	41.27 (1.55)
Lifting	25.89 (2.14)	65.72 (10.31)	1.71 (0.10)	22.31 (1.90)	40.72 (2.27)

**Table 2:** Comparison between each variable estimated with Pedar-X^®^ and with the simulated layout for each insole size. Below, the units of measurement of each variable are provided: Peak Pressure [%BW/mm^2^], Mean Pressure [%BW/mm^2^], GRF [%BW], dCOP [%Surface Area], Medial-Lateral (Med-Lat) COP [mm], Anterior-Posterior (Ant-Post) COP [mm], and Contact Area [%Surface Area]. RMSE was calculated in terms of percentage of the corresponding value in the gold standard (Pedar-X^®^).

	**38–39** **Mean ± SD**		**38–39** **RMSE %**	**40–41** **Mean ± SD**		**40–41** **RMSE %**
	**Pedar-X^®^**	**Simulated Layout**		**Pedar-X^®^**	**Simulated Layout**	
Peak Pressure	24.1 ± 6	19.1 ± 4.2	<7.6–22.6>	18.9 ± 6.5	15.3 ± 4.6	<9.7–16.9>
Mean Pressure	3.6 ± 0.4	1.1 ± 0.1	<36.8–64>	2.8 ± 0.6	0.8 ± 0.2	<32–54.5>
GRF	74.3 ± 8.4	22.6 ± 2.9	<35.8–63.7>	62 ± 12.8	17.9 ± 3.8	<32–54.4>
dCOP	0.6 ± 0.07	0.7 ± 0.07	<4.2–15.9>	0.7 ± 0.09	0.7 ± 0.1	<2.2–8.1>
Med-Lat COP	121.9 ± 57	131.2 ± 58.4	<0.7–5.9>	140.9 ± 66.9	149.5 ± 71	<4.5–8.6>
Ant-Post COP	30.4 ± 6.4	29.2 ± 7	<2.3–7.9>	31.8 ± 7.5	33.2 ± 7.5	<0.8–5.8>
Contact Area	62.6 ± 9.2	18.7 ± 3	<43–73.5>	60.6 ± 13.2	16.2 ± 3.8	<49–77>
	**42–43** **Mean ± SD**		**42–43** **RMSE %**	**44–45** **Mean ± SD**		**44–45** **RMSE %**
	**Pedar-X^®^**	**Simulated Layout**		**Pedar-X^®^**	**Simulated Layout**	
Peak Pressure	27.1 ± 4.6	20.6 ± 4	<10.6–34.7>	16.6 ± 2.5	13.7 ± 1.8	<6.2–40.2>
Mean Pressure	2.3 ± 0.2	0.8 ± 0.1	<42–69.8>	2.8 ± 0.1	0.6 ± 0.04	<40.9–82.4>
GRF	56.2 ± 5.3	19.1 ± 2	<41.1–69.5>	75.8 ± 3.5	15.4 ± 1.2	<41–82.4>
dCOP	0.7 ±0.05	0.7 ± 0.05	<0.59–13>	0.6 ± 0.05	0.6 ± 0.06	<6.6–12.1>
Med-Lat COP	108.7 ± 58.1	117.7 ± 60.5	<2.2–7.9>	144.3 ± 64	152.5 ± 70	<6.1–11.1>
Ant-Post COP	45.6 ± 6.2	42.6 ± 7	<0.6–5.9>	49.6 ± 8.4	43 ± 8.7	<1.6–9.4>
Contact Area	62.9 ± 9	21.6 ± 2.7	<48.3–78.6>	65.8 ± 7.6	13.9 ± 2	<52.1–83.5>

**Table 3:** Comparison between each variable estimated with Pedar-X^®^ and with the simulated layout for each insole size. Below, the units of measurement of each variable are reported: Peak Pressure [%BW/mm^2^], Mean Pressure [%BW/mm^2^], GRF [%BW], dCOP [%Surface Area], Medial-Lateral (Med-Lat) COP [mm], Anterior-Posterior (Ant-Post) COP [mm], and Contact Area [%Surface Area]. The RMSE was calculated in terms of percentage of the corresponding value in the gold standard (Pedar-X^®^).

	**38–39** **Mean ± SD**		**38–39** **RMSE %**	**40–41** **Mean ± SD**		**40–41** **RMSE %**
	**Pedar-X^®^**	**Simulated Layout**		**Pedar-X^®^**	**Simulated Layout**	
Peak Pressure	34.3 ± 12.7	22.8 ± 6.6	<21.5–36.7>	30.7 ± 8.7	23.6 ± 6	<14.3–39.7>
Mean Pressure	4.4 ± 0.4	1.4 ± 0.2	<38–69>	4.4 ± 0.5	1.2 ± 0.2	<36.5–59>
GRF	94.9 ± 8.2	28.8 ± 4.6	<40.6–68.9>	97.7 ± 10.6	27.8 ± 4	<38–68.9>
dCOP	0.8 ± 0.1	0.9 ± 0.1	<2.6–9.9>	0.8 ± 0.09	0.8 ± 0.07	<0.9–8.1>
Med-Lat COP	136 ± 17.3	148 ± 17.1	<2.7–7.4>	158 ± 14.9	171.9 ± 15.8	<4.9–10.1>
Ant-Post COP	29.5 ± 3.5	29.2 ± 3.6	<3–7.8>	37.8 ± 3.3	38.8 ± 3.5	<1.9–5.5>
Contact Area	68.8 ± 12.2	21.4 ± 3	<53.5–74.4>	74.5 ± 9.7	20 ± 1.9	<60.6–77.8>
	**42–43** **Mean ± SD**		**42–43** **RMSE %**	**44–45** **Mean ± SD**		**44–45** **RMSE %**
	**Pedar-X^®^**	**Simulated Layout**		**Pedar-X^®^**	**Simulated Layout**	
Peak Pressure	29.4 ± 11	20.7 ± 8.5	<14–24.9>	28.2 ± 13.9	22.1 ± 11.3	<7.8–25.9>
Mean Pressure	3.7 ± 0.7	0.9 ± 0.2	<29.2–63.7>	4.1 ± 0.4	1.1 ± 0.3	<39.7–72.1>
GRF	89.6 ± 16.8	22.6 ± 4.4	<31.8–63.7>	110.2 ± 10.4	28.8 ± 8.1	<41.3–72.2>
dCOP	0.7 ± 0.1	0.8 ± 0.01	<2.5–9.1>	0.7 ± 0.08	0.7 ± 0.1	<5.8–10.6>
Med-Lat COP	142.3 ± 13.4	154.1 ± 13.1	<6.3–8.1>	149.6 ± 15.3	165.6 ± 15	<3.2–6.9>
Ant-Post COP	42.2 ± 3.9	38 ± 3.9	<2.9–7.5>	44.3 ± 2.5	38.4 ± 2.4	<1.5–8.1>
Contact Area	73.1 ± 14.5	17.8 ± 2.3	<63.7–78.3>	77.9 ± 13.5	17 ± 1.2	<61.5–81.6>

**Table 4:** Comparison between each variable estimated with Pedar-X^®^ and with the simulated layout for each insole size. Below, the units of measurement of each variable are provided: Peak Pressure [%BW/mm^2^], Mean Pressure [%BW/mm^2^], GRF [%BW], dCOP [%Surface Area], Medial-Lateral (Med-Lat) COP [mm], Anterior-Posterior (Ant-Post) COP [mm], and Contact Area [%Surface Area]. The RMSE was calculated in terms of percentage of the corresponding value in the gold standard (Pedar-X^®^).

	**38–39** **Mean ± SD**		**38–39** **RMSE %**	**40–41** **Mean ± SD**		**40–41** **RMSE %**
	**Pedar-X^®^**	**Simulated Layout**		**Pedar-X^®^**	**Simulated Layout**	
Peak Pressure	17.8 ± 3.9	12.8 ± 3	<16.4–26.3>	23.1 ± 9.5	15.9 ± 3.9	<18.5–29.2>
Mean Pressure	2.4 ± 0.5	0.7 ± 0.1	<43.2–54.8>	2.2 ± 0.7	0.6 ± 0.2	<44.6–51.3>
GRF	49.9 ± 11.2	14.6 ± 2.9	<43.1–54.6>	48.5 ± 15.3	13.4 ± 3.7	<44.5–51.3>
dCOP	0.6 ± 0.1	0.6 ± 0.2	<7.3–9.2>	0.4 ± 0.1	0.4 ± 0.1	<4.4–8>
Med-Lat COP	89.2 ± 2.7	97.9 ± 3.4	<3.9–5.2>	100.2 ± 7.2	110.2 ± 8.4	<5.2–6.9>
Ant-Post COP	29 ± 0.6	27.9 ± 0.6	<2.5–4.3>	29.6 ± 1.1	30.1 ± 1.1	<3.5–5.3>
Contact Area	83.7 ± 12.9	29.5 ± 7.2	<62.9–66.2>	71.6 ± 19.4	21.9 ± 6.9	<66.7–71.1>
	**42–43** **Mean ± SD**		**42–43** **RMSE %**	**44–45** **Mean ± SD**		**44–45** **RMSE %**
	**Pedar-X^®^**	**Simulated Layout**		**Pedar-X^®^**	**Simulated Layout**	
Peak Pressure	14.8 ± 4.2	12 ± 3.5	<11.3–14.6>	15.5 ± 4	9.9 ± 2.3	<18.1–45.2>
Mean Pressure	2 ± 0.4	0.5 ± 0.1	<44.9–62.4>	2.1 ± 0.2	0.5 ± 0.05	<61.8–69.6>
GRF	47.4 ± 8.6	13 ± 3.1	<44.8–62.3>	56.9 ± 6.5	12.6 ± 1.4	<61.7–69.5>
dCOP	0.5 ± 0.1	0.5 ± 0.2	<8.5–13.3>	0.6 ± 0.1	0.6 ± 0.1	<9.2–14.1>
Med-Lat COP	77 ± 3.6	82.3 ± 4.6	<3.9–5>	96.9 ± 7.3	102.9 ± 8.3	<7.5–10.9>
Ant-Post COP	50.3 ± 0.8	46.2 ± 0.7	<4.1–5>	51.1 ± 0.8	46.3 ± 0.9	<1.4–4.5>
Contact Area	77.1 ± 12.7	23.1 ± 6.4	<68.3–71.5>	81.8 ± 9.5	20.5 ± 3.4	<72.6–75.8>

**Table 5:** Comparison between each variable estimated with Pedar-X^®^ and with the Simulated layout for each insole size. Below, the units of measurement of each variable: Peak Pressure [%BW/mm^2^], Mean Pressure [%BW/mm^2^], GRF [%BW], dCOP [%Surface Area], Medial-Lateral (Med-Lat) COP [mm], Anterior-Posterior (Ant-Post) COP [mm] and Contact Area [%Surface Area]. The RMSE was calculated in terms of percentage of the corresponding value in the gold standard (Pedar-X^®^).

	**38–39** **Mean ± SD**		**38–39** **RMSE %**	**40–41** **Mean ± SD**		**40–41** **RMSE %**
	**Pedar-X^®^**	**Simulated Layout**		**Pedar-X^®^**	**Simulated Layout**	
Peak Pressure	20.5 ± 6	14 ± 2.7	<17.2–26.3>	21.3 ± 6.7	14.4 ± 3.6	<21.6–31.3>
Mean Pressure	2.7 ± 0.5	0.8 ± 0.2	<49.6–56.6>	2.5 ± 0.7	0.7 ± 0.2	<44.4–51.1>
GRF	56 ± 10.2	17.2 ± 3.3	<49.5–56.5>	56.1 ± 14.5	15.7 ± 4	<44.3–51>
dCOP	0.6 ± 0.1	0.6 ± 0.2	<7.5–9.7>	0.6 ± 0.1	0.6 ± 0.2	<7.2–8.5>
Med-Lat COP	107.1 ± 4.3	120 ± 5.4	<4.1–4.7>	130.3 ± 4.2	142.3 ± 4.1	<5.2–7.3>
Ant-Post COP	31 ± 0.6	30.3 ± 0.7	<2.2–5>	33.4 ± 0.9	34.7 ± 0.9	<5–6.4>
Contact Area	84.4 ± 11	29.9 ± 6.1	<63.8–65.4>	83.7 ± 14.7	24.9 ± 5.4	<66.5–69.4>
	**42–43** **Mean ± SD**		**42–43** **RMSE %**	**44–45** **Mean ± SD**		**44–45** **RMSE %**
	**Pedar-X^®^**	**Simulated Layout**		**Pedar-X^®^**	**Simulated Layout**	
Peak Pressure	21.5 ± 9.9	16.8 ± 7.1	<11–15.3>	19 ± 7.7	10.4 ± 2.5	<19.8–42.3>
Mean Pressure	2.4 ± 0.4	0.7 ± 0.1	<45–59.6>	2.2 ± 0.3	0.5 ± 0.1	<57.7–70.9>
GRF	58.4 ± 10.5	16.4 ± 3.4	<45–59.5>	59.3 ± 8.2	13.7 ± 2.3	<57.5–70.8>
dCOP	0.6 ± 0.2	0.7 ± 0.2	<7.5–9.7>	0.6 ± 0.1	0.6 ± 0.2	<3.2–5.6>
Med-Lat COP	102.1 ± 3.9	111.2 ± 4.8	<4.9–6.3>	126.8 ± 5.2	135.2 ± 5.4	<9–12>
Ant-Post COP	47.2 ± 0.5	43.1 ± 0.6	<4.5–6.4>	47.7 ± 0.9	41.6 ± 1	<2.2–5>
Contact Area	82.9 ± 13.1	24.5 ± 6.2	<70.3–71.5>	83.7 ± 9.2	21.7 ± 2.6	<72–75.1>

**Table 6:** Comparison between each variable estimated with Pedar-X^®^ and with the Simulated layout during loaded descending for each insole size. Below, the units of measurement of each variable: Peak Pressure [%BW/mm^2^], Mean Pressure [%BW/mm^2^], GRF [%BW], dCOP [%Surface Area], Medial-Lateral (Med-Lat) COP [mm], Anterior-Posterior (Ant-Post) COP [mm] and Contact Area [%Surface Area]. The RMSE was calculated in percentage of the corresponding value in the gold standard (Pedar-X^®^).

	**38–39** **Mean ± SD**		**38–39** **RMSE %**	**40–41** **Mean ± SD**		**40–41** **RMSE %**
	**Pedar-X^®^**	**Simulated layout**		**Pedar-X^®^**	**Simulated layout**	
Peak Pressure	19.9 ± 5.5	14 ± 2.9	<16.2–23>	25 ± 10.3	16.2 ± 6	<17.9–28.6>
Mean Pressure	2.6 ± 0.5	0.8 ± 0.2	<46.5–54.4>	2.5 ± 0.7	0.7 ± 0.2	<45.1–50.7>
GRF	54.1 ± 11.2	16.7 ± 3.3	<46.4–54.3>	54.8 ± 16.2	15.2 ± 3.7	<45.1–50.7>
dCOP	0.6 ± 0.1	0.6 ± 0.2	<7.2–9.4>	0.5 ± 0.2	0.6 ± 0.2	<5.4–7.2>
Med-Lat COP	99.3 ± 6.9	110.2 ± 8.2	<3.9–5.2>	121.9 ± 9.1	133.4 ± 9.5	<5.7–7.6>
Ant-Post COP	30.9 ± 1.1	30 ± 1.2	<2.9–5.2>	33.1 ± 1.1	34 ± 1.3	<3.2–5.8>
Contact Area	84.2 ± 10.5	29.9 ± 6.3	<63.1–65.3>	74.2 ± 16.4	23.3 ± 6.2	<65.5–71>
	**42–43** **Mean ± SD**		**42–43** **RMSE %**	**44–45** **Mean ± SD**		**44–45** **RMSE %**
	**Pedar-X^®^**	**Simulated layout**		**Pedar-X^®^**	**Simulated layout**	
Peak Pressure	21.5 ± 8.7	16.3 ± 6	<13.3–17.8>	15.9 ± 4	10.7 ± 2	<15.6–31.1>
Mean Pressure	2.3 ± 0.4	0.6 ± 0.1	<46.2–58.4>	2.4 ± 0.2	0.5 ± 0.05	<62.2–72.2>
GRF	55.8 ± 9.9	15.9 ± 3.6	<46.2–58.3>	63.7 ± 5.4	14.6 ± 1.3	<62.1–72.1>
dCOP	0.6 ± 0.2	0.7 ± 0.2	<6.2–8.2>	0.6 ± 0.1	0.6 ± 0.1	<3.7–6.6>
Med-Lat COP	92.4 ± 8.4	100.9 ± 8.8	<4.5–5.9>	122.7 ± 9.3	127.7 ± 9.8	<8.7–9.9>
Ant-Post COP	47.9 ± 1.7	43.5 ± 1.4	<3.6–4.7>	48.5 ± 1.1	43.3 ± 1.3	<1.9–3.9>
Contact Area	82.2 ± 11.6	23.8 ± 4.4	<67–71.9>	82.8 ± 10.2	20.7 ± 3	<74.1–76.3>

**Table 7:** Comparison of each variable during unloaded ascending between Pedar-X^®^ and simulated layout for each insole size. Below, the units of measurement of each variable are provided: Peak Pressure [%BW/mm^2^], Mean Pressure [%BW/mm^2^], GRF [%BW], dCOP [%Surface Area], Medial-Lateral (Med-Lat) COP [mm], Anterior-Posterior (Ant-Post) COP [mm], and Contact Area [%Surface Area]. The RMSE was calculated in terms of percentage of the corresponding value in the gold standard (Pedar-X^®^).

	**38–39** **Mean ± SD**		**38–39** **RMSE %**	**40–41** **Mean ± SD**		**40–41** **RMSE %**
	**Pedar-X^®^**	**Simulated layout**		**Pedar-X^®^**	**Simulated layout**	
Peak Pressure	18.2 ± 3.2	13.7 ± 3	<20–23.1>	22.7 ± 6	16.1 ± 3.2	<23.2–25.7>
Mean Pressure	2.6 ± 0.5	0.8 ± 1.4	<45.8–54.4>	2.4 ± 0.6	0.7 ± 1.4	<47.4–52.7>
GRF	53.6 ± 10.3	16.2 ± 2.9	<45.6–54.3>	53.3 ± 12.8	14.7 ± 3.2	<47.3–52.6>
dCOP	0.6 ± 0.1	0.6 ± 0.1	<8.2–10.1>	0.5 ± 0.2	0.6 ± 0.2	<5.7–8>
Med-Lat COP	98.9 ± 5.1	109.4 ± 7	<4.5–5>	104.7 ± 9.5	114.4 ± 11	<4.4–5.8>
Ant-Post COP	31.1 ± 0.9	29.9 ± 1.2	<3.7–4.9>	32.2 ± 0.9	32.3 ± 1.3	<5.4–6.2>
Contact Area	85 ± 11.6	29.9 ± 6.6	<62.5–65.7>	75.3 ± 17.6	23.3 ± 6.4	<67.9–70.9>
	**42–43** **Mean ± SD**		**42–43** **RMSE %**	**44–45** **Mean ± SD**		**44–45** **RMSE %**
	**Pedar-X^®^**	**Simulated layout**		**Pedar-X^®^**	**Simulated layout**	
Peak Pressure	18.3 ± 5.3	14.5 ± 3.3	<12–17.1>	12.4 ± 2.8	9.6 ± 2.1	<14.6–23.9>
Mean Pressure	2.2 ± 0.4	0.6 ± 0.1	<52.1–59.5>	1.9 ± 0.2	0.4 ± 0.1	<59–70.4>
GRF	53.9 ± 9.8	14.3 ± 2.3	<52–59.5>	51.6 ± 6.3	11.7 ± 1.4	<58.9–70.4>
dCOP	0.5 ± 0.1	0.5 ± 0.1	<7.8–10>	0.5 ± 0.1	0.5 ± 0.1	<2.6–7.8>
Med-Lat COP	91 ± 5.1	100.1 ± 5.8	<4–4.6>	102.2 ± 13.4	108 ± 15.1	<8.2–9.5>
Ant-Post COP	48.1 ± 0.7	44.2 ± 0.7	<3.9–5.4>	48.9 ± 1.2	44.3 ± 1.4	<1–4.2>
Contact Area	80.7 ± 13.5	23.7 ± 5.2	<69.2–71.2>	82.4 ± 6	20.6 ± 2.5	<73.7–76.2>

**Table 8:** Details of studies in terms of: Instruments = type of Instruments used; Validation: done or not (Yes or Not); if Yes, which instruments were used to validate (details of instruments), or which procedures were applied for validation; Biomechanical Variables = which variables were extracted.

References	Instruments	Validation			Biomechanical Variables
		Yes or No	Details of Instruments	Details of Procedures	
Lavery, L.A. et al., 1991	Novel Pedar X	No			Peak plantar pressure
Mueller, M.J. et al., 2003	F-Scan, Tekscan	No			Peak plantar pressure
Chen, M. et al., 2008	Shoe-Integrated System	No			No
Mueller, M.J. et al., 1999	//	No			Peak pressure and contact area
Hodgson, B. et al., 2006	EMED Pedar in-shoe plantar-pressure system	No			Plantar pressure
Morris Bamberg, S.J. et al., 2008	Gaitshoe	Yes		Massachusetts general hospital, biomotion laboratory	Gait parameters
Price, C. et al., 2016	Shoe-Integrated Wireless Sensor System	Yes	Medilogic, Tekscan and Pedar		Plantar pressure and contact area
Preece, S.J. et al., 2011	Instrumented Sock Wearable Textile Sensor Socks	No			Sensor output and ankle angle
Tirosh, O. et al., 2013	Sensor Socks	Yes	Tekscan, Inc.		Temporal parameters
Oks, A. et al., 2016	Daid^®^ Pressure Sock System	Yes		Made by comparative gait analysis of different running and walking modes of asymptomatic and flat foot	Temporal gait analysis, plantar pressure detection
Stöggl, T. et al., 2017	Opengo Sensor Insole	Yes		Pedarx sensor insole and AMTI force-plate systems	Ground contact and flight times
Zizoua, C. et al., 2014	Wireless Sensor System Equipped with Force Sensing Resistors (Fsr)	No			Pressure distributions
De Rossi, S. et al., 2011	In-Shoe Device	Yes	Validation on a healthy subject		Plantar pressure
Aqueveque, P. et al., 2020	Sensorized Insoles	Yes		Two instrumented insoles were implemented in order to perform experimental walking pressure validation tests	Plantar pressure
Lin, F. et al., 2016	Smart Insole	Yes		Collecting complete gait parameters and further extracting useful features	Gait parameters and features
Shu, L. et al., 2010	In-Shoe Plantar Pressure Measurement and Analysis	Yes		To verify if the integrationof the measured force on the feet surface gives a value that is close to the body weight of the subject	Mean pressure, peak pressure, center of pressure (cop), and shift speed of cop
DeBerardinis, J. et al., 2018	Systmedilogic^®^ Pressure-Measuring Insoles (Schönefeld, Germany)	Yes	Force platform measurements		Stance time and support-phase
Mokhlespour et al., 2019	Smart Socks	No			No

**Table 9:** Minimum and maximum values of RMSE% using the Novel Pedar-X^®^ system for estimates from GRF during self-selected speed and drop landing trials in our study, in comparison to the ones assessed by Stöggl and Martiner [16] during fast walking, slow walking, and the drop jump test.

GRF–RMSE%	Fast Walking	Slow Walking	Walking	Drop Jump Test	Drop Landing Test
Stöggl and Martiner [20]	<47.9–74.9> %	<55.1–76.4> %	/	<35.1–47.5> %	/
Simulated Layout	/	/	<32–82> %	/	<41.3–72.2> %

## Data Availability

Not applicable.

## Supplementary Materials

The following are available online at https://www.mdpi.com/1424-8220/21/4/1450/s1, From Figures S1–S3: Peak and Mean Pressure of the left foot, Mean Pressure of the right foot are displayed for each insole size in %BW/mm^2^ during gait analysis. Experimental (Pedar-X^®^ system), simulated (prototype layout), and loss of data (experimental-simulated) footprints are represented. In yellow/white the higher pressure, in blue/black the lower pressure. From Figures S4–S14: the temporal distribution of Peak Pressure, Mean Pressure, GRF, dCOP, Medial-Lateral and Anterior-Posterior COP, Contact Area, and RMSE (calculated on each variable in terms of percentage of the corresponding value in the gold standard Pedar-X^®^) were reported for each insole size during gait. From Figures S15–S17: Peak and Mean Pressure of the left foot and Mean Pressure of the right foot are displayed for each insole size in %BW/mm^2^ during drop. Experimental (Pedar-X^®^ system), simulated (prototype layout), and loss of data (experimental-simulated) footprints are represented. In yellow/white the higher pressure, in blue/black the lower pressure. From Figures S18–S28: the temporal distribution of Peak Pressure, Mean Pressure, GRF, dCOP, Medial-Lateral and Anterior-Posterior COP, Contact Area, and RMSE (calculated on each variable in terms of percentage of the corresponding value in the gold standard Pedar-X^®^) were reported for each insole size during drop. From Figures S29–S31: Peak and Mean Pressure of the left foot, and Mean Pressure of the right foot are displayed for each insole size in %BW/mm^2^ during unloaded descending (UD). Experimental (Pedar-X^®^ system), simulated (prototype layout), and loss of data (experimental-simulated) footprints are represented. In yellow/white the higher pressure, in blue/black the lower pressure. From Figures S32–S42: the temporal distribution of Peak Pressure, Mean Pressure, GRF, dCOP, Medial-Lateral and Anterior-Posterior COP, Contact Area, and RMSE (calculated on each variable in terms of percentage of the corresponding value in the gold standard Pedar-X^®^) were reported for each insole size during UD. From Figures S43–S45: Peak and Mean Pressure of the left foot, and Mean Pressure of the right foot are displayed for each insole size in %BW/mm^2^ during loaded ascending (LA). Experimental (Pedar-X^®^ system), simulated (prototype layout), and loss of data (experimental-simulated) footprints are represented. In yellow/white the higher pressure, in blue/black the lower pressure. From Figures S46–S56: the temporal distribution of Peak Pressure, Mean Pressure, GRF, dCOP, Medial-Lateral and Anterior-Posterior COP, Contact Area, and RMSE (calculated on each variable in terms of percentage of the corresponding value in the gold standard Pedar-X^®^) were reported for each insole size during LA. From Figures S57–S59: Peak and Mean Pressure of the left foot, and Mean Pressure of the right foot are displayed for each insole size in %BW/mm^2^ during loaded descending (LD). Experimental (Pedar-X^®^ system), simulated (prototype layout), and loss of data (experimental-simulated) footprints are represented. In yellow/white the higher pressure, in blue/black the lower pressure. From Figures S60–S70: the temporal distribution of Peak Pressure, Mean Pressure, GRF, dCOP, Medial-Lateral and Anterior-Posterior COP, Contact Area, and RMSE (calculated on each variable in percentage of the corresponding value in the gold standard Pedar-X^®^) were reported for each insole size during LD. From Figures S71–S73: Peak and Mean Pressure of the left foot, and Mean Pressure of the right foot are displayed for each insole size in %BW/mm^2^ during unloaded ascending (UA). Experimental (Pedar-X^®^ system), simulated (prototype layout), and loss of data (experimental-simulated) footprints are represented. In yellow/white the higher pressure, in blue/black the lower pressure. From Figures S74–S84: the temporal distribution of Peak Pressure, Mean Pressure, GRF, dCOP, Medial-Lateral and Anterior-Posterior COP, Contact Area, and RMSE (calculated on each variable in terms of percentage of the corresponding value in the gold standard Pedar-X^®^) were reported for each insole size during UA.
