# Supplementary material for: The Design and Simulation of a 16-Sensors Plantar Pressure Insole Layout for Different Applications: From Sports to Clinics, a Pilot Study
Source: Sensors (Basel). 2021 Feb 19;21(4):1450. doi: 10.3390/s21041450 (PMC7922081; doi:10.3390/s21041450)
Supplement: Supplementary file 1 [file sensors-21-01450-s001.pdf]

Supplementary Material

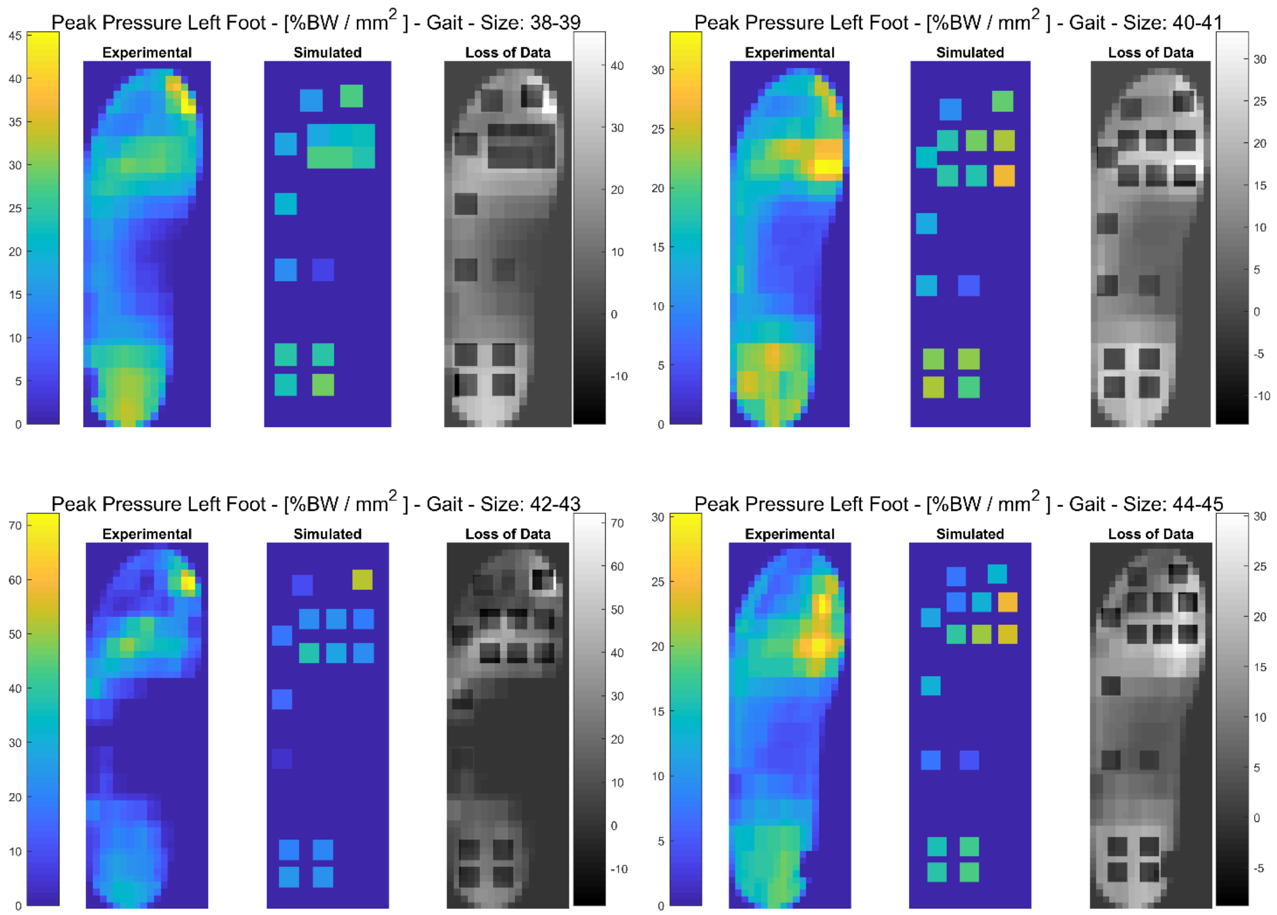

Figure S1: Peak Pressure of left foot for each insole size in %BW/mm^2 during gait. Experimental (Pedar -X® system), simulated (prototype layout) and loss of data (experimental - simulated) footprints were reported. In yellow/white the higher pressure, in blue/black the lower pressure.

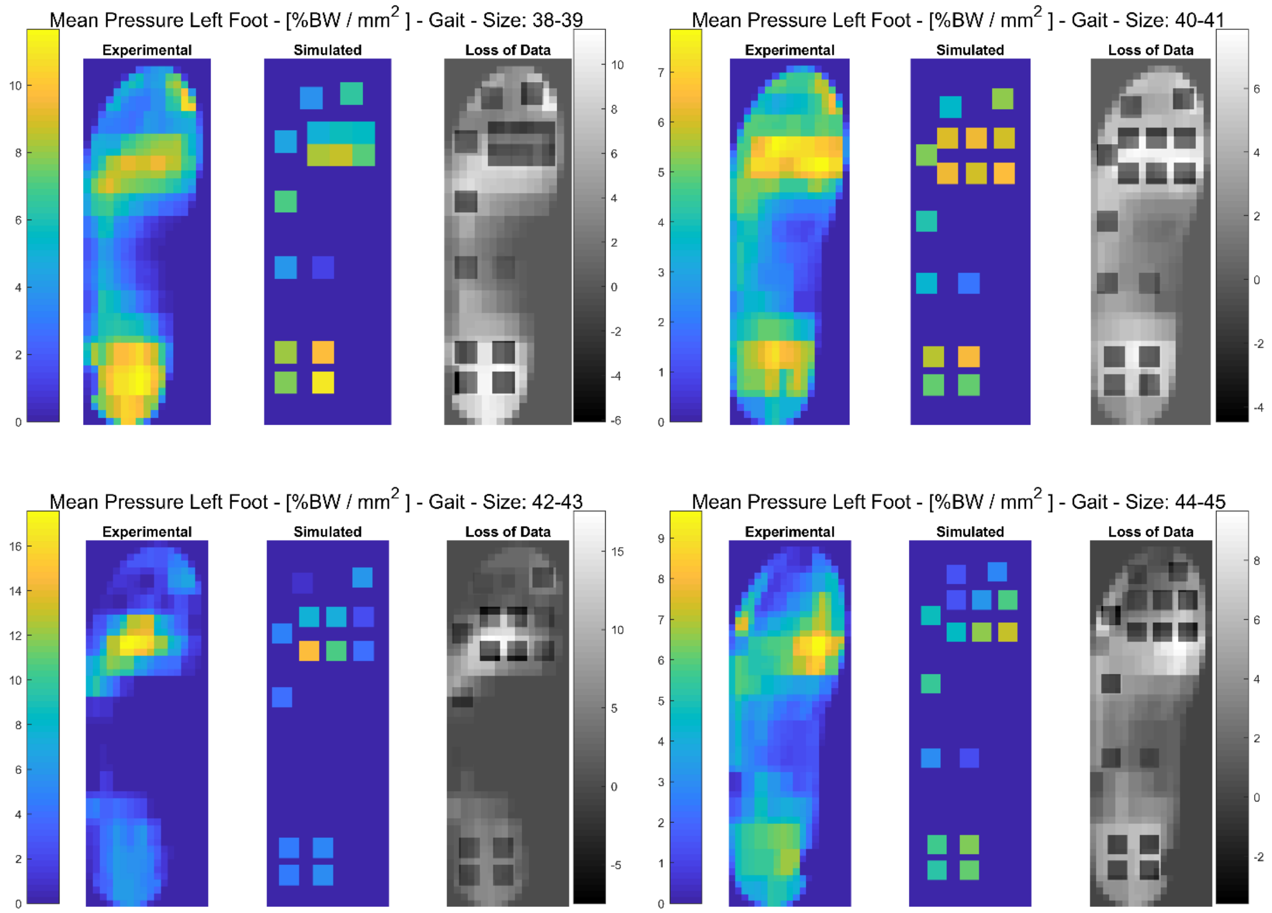

Figure S2: Mean Pressure of left foot for each insole size in %BW/mm^2 during gait. Experimental (Pedar -X® system), simulated (prototype layout) and loss of data (experimental - simulated) footprints were reported. In yellow/white the higher pressure, in blue/black the lower pressure.

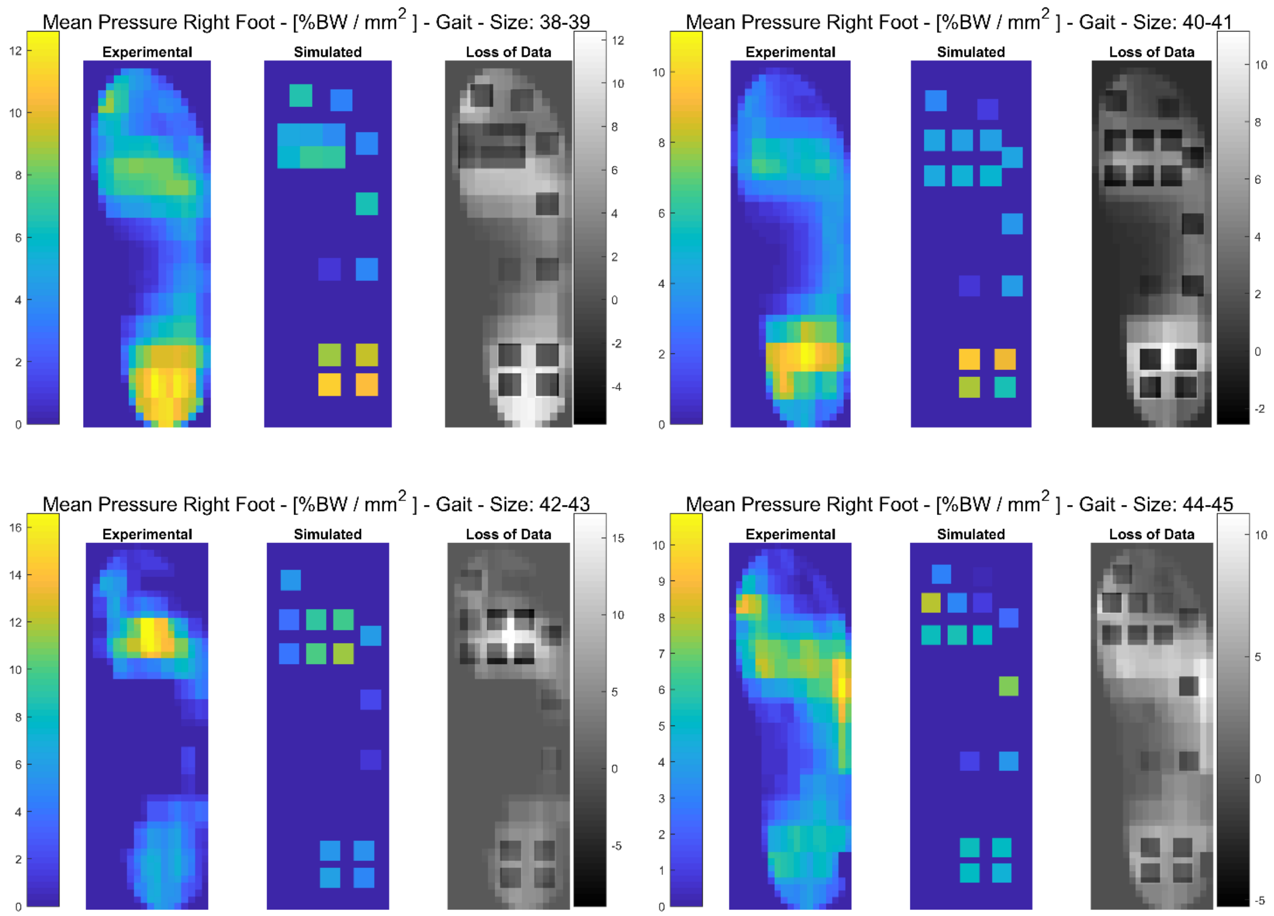

Figure S3: Mean Pressure of right foot for each insole size in %BW/mm^2 during gait. Experimental (Pedar -X® system), simulated (prototype layout) and loss of data (experimental - simulated) footprints were reported. In yellow/white the higher pressure, in blue/black the lower pressure.

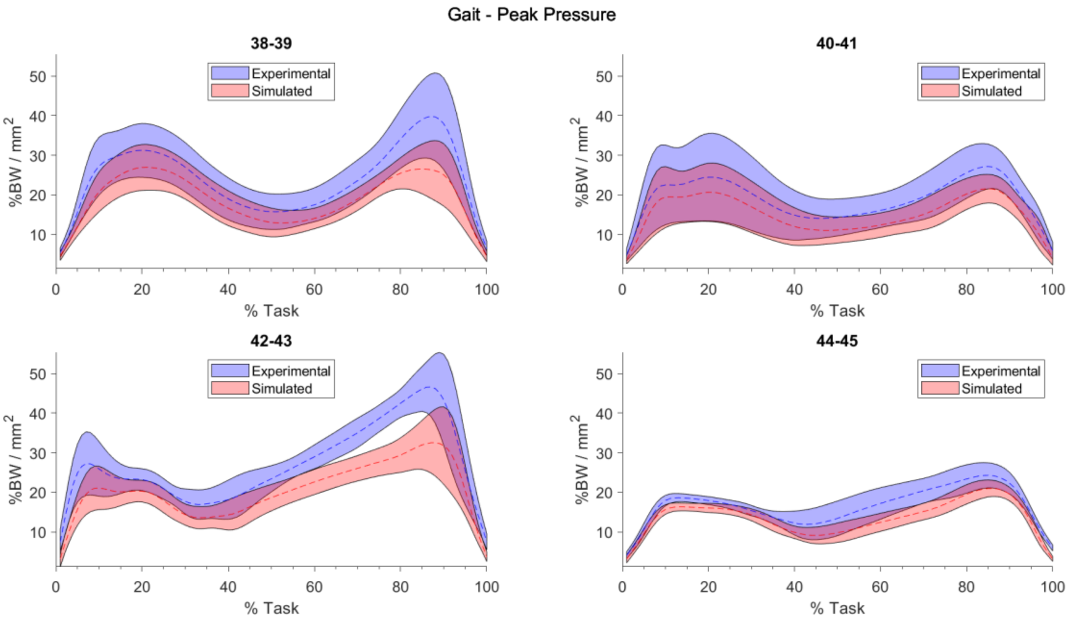

Figure S4: Peak Pressure for each insole size %BW/mm^2 during gait, expressed in terms of mean and standard deviation. Experimental (Pedar -X® system) in blue and Simulated (prototype layout) in red. Left and right insoles data were averaged.Figure B5: Peak Pressure for each insole size of body weight during

gait, expressed in terms of mean and standard deviation. Experimental (Pedar -X® system) in blue and Simulated (prototype layout) in red. Left and right insoles data were averaged.

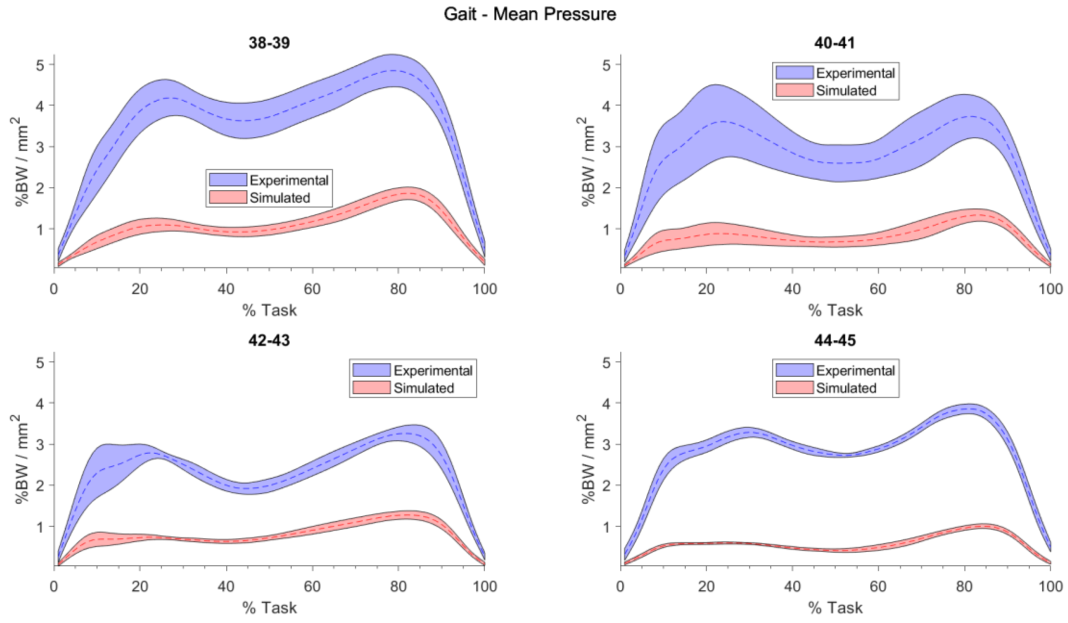

Figure S5: Mean Pressure for each insole size in %BW/mm² during gait, expressed in terms of mean and standard deviation. Experimental (Pedar -X® system) in blue and Simulated (prototype layout) in red. Left and right insoles data were averaged.

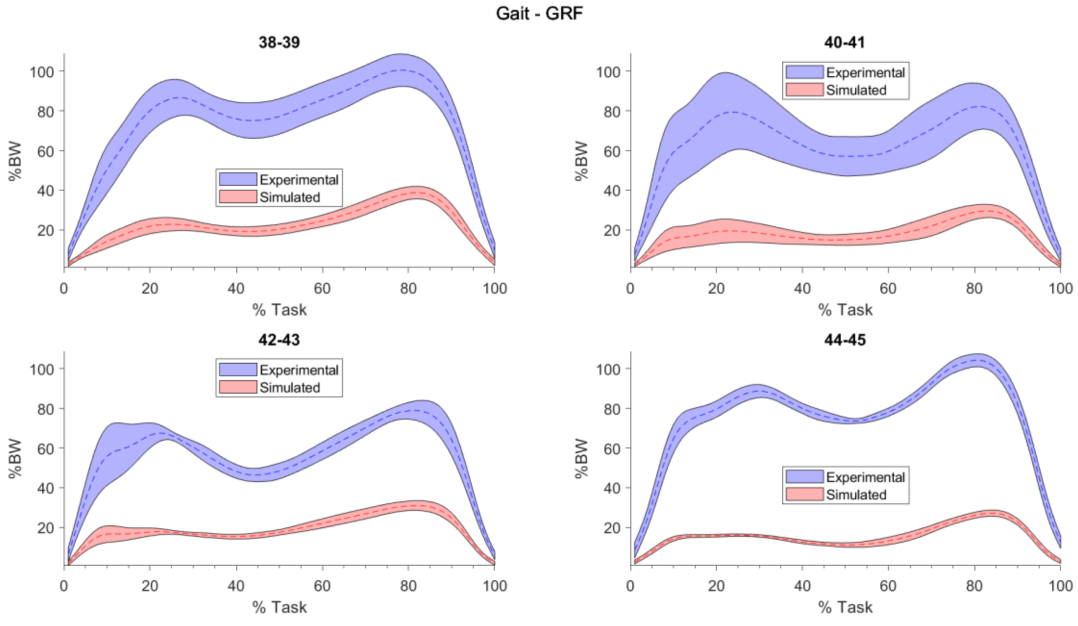

Figure S6: Ground Reaction Force (GRF) for each insole size in percentage of body weight during gait, expressed in terms of mean and standard deviation. Experimental (Pedar -X® system) in blue and Simulated (prototype layout) in red. Left and right insoles data were averaged.

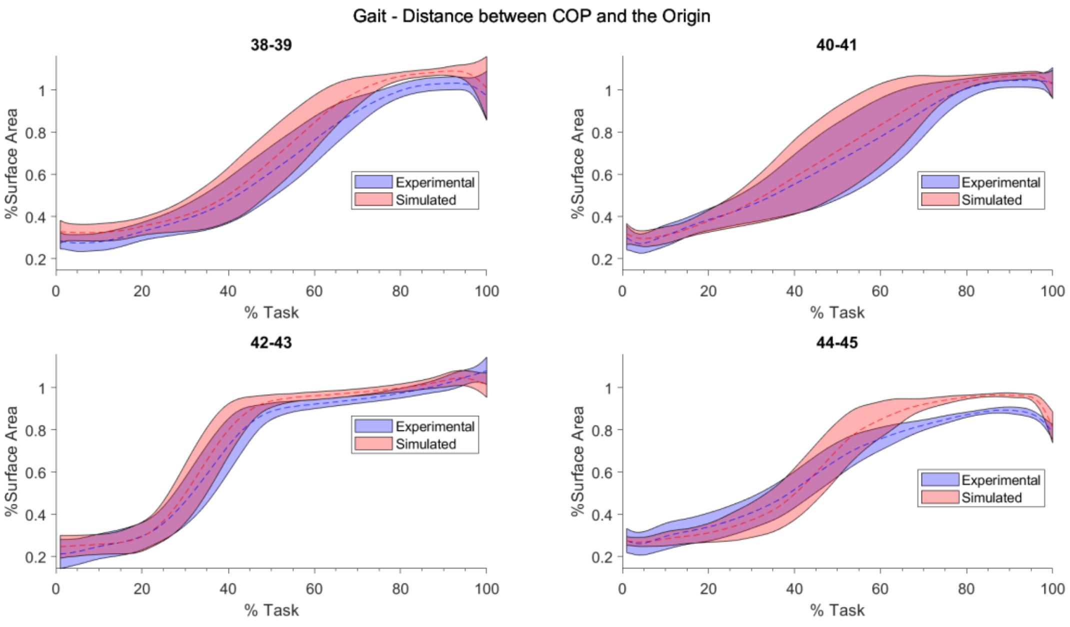

Figure S7: Distance between Center of Pressure (COP) and the Origin for each insole size in percentage of surface area during gait, expressed in terms of mean and standard deviation. Experimental (Pedar -X® system) in blue and Simulated (prototype layout) in red. Left and right insoles data were averaged.

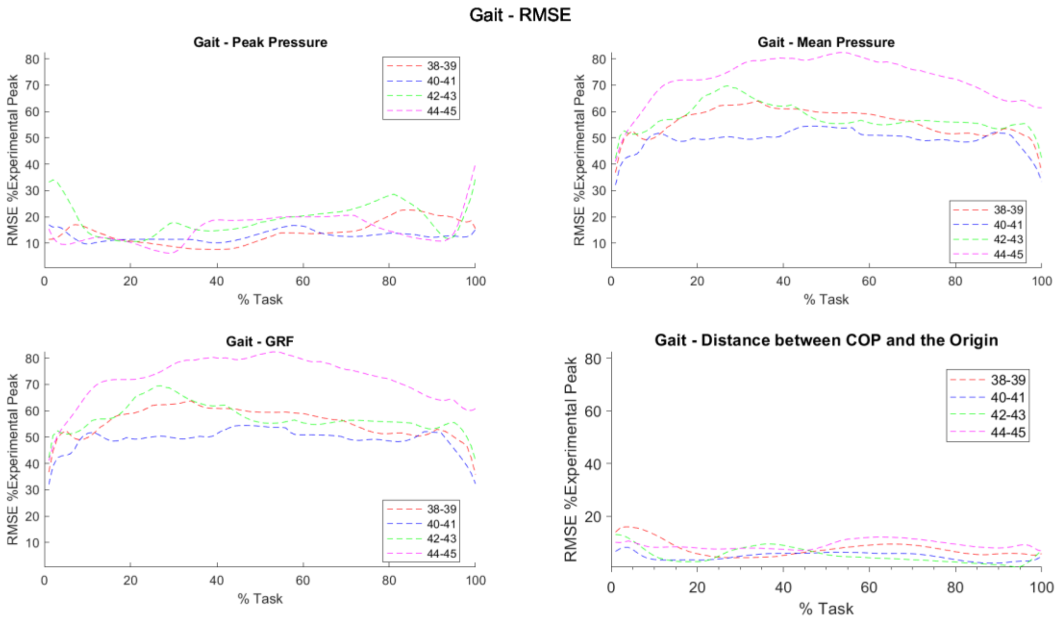

Figure S8: Root Mean Square Error (RMSE) in percentage of Experimental Peak (Pedar -X® system) for each variable and for each insole size during gait. The insoles were represented respectively: 38-39 in red, 40-41 in blue, 42-43 in green and 44-45 in purple.

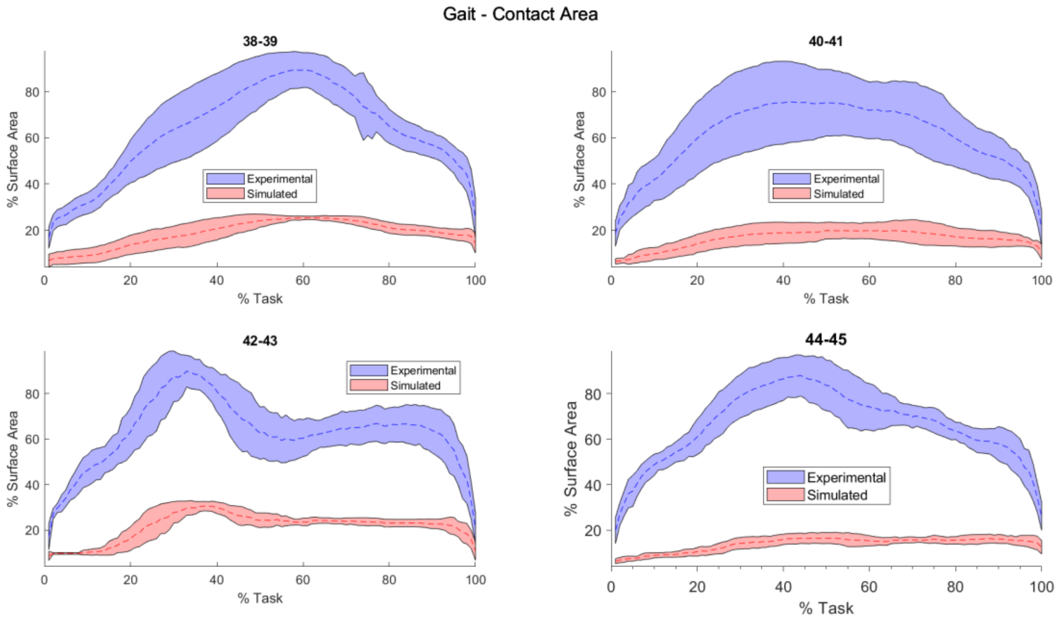

Figure S9: Contact Area for each insole size in percentage of surface area during gait, expressed in terms of mean and standard deviation. Experimental (Pedar -X® system) in blue and Simulated (prototype layout) in red. Left and right insoles data were averaged.

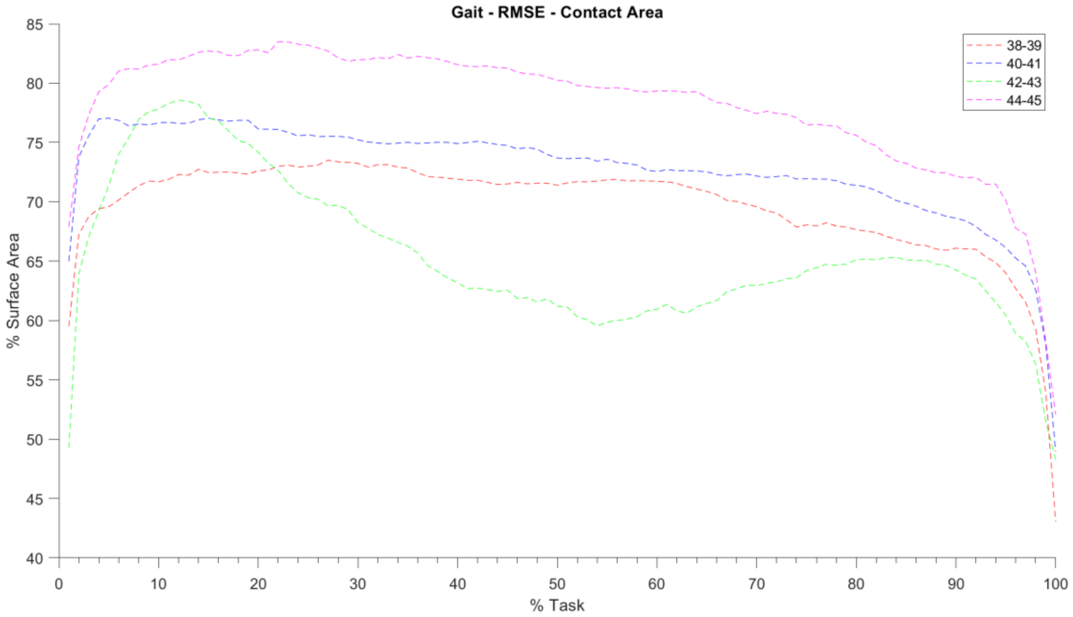

Figure S10: Contact Area Root Mean Square Error (RMSE) in percentage of Surface area for each insole size during gait. The insoles were represented respectively: 38-39 in red, 40-41 in blue, 42-43 in green and 44-45 in purple.

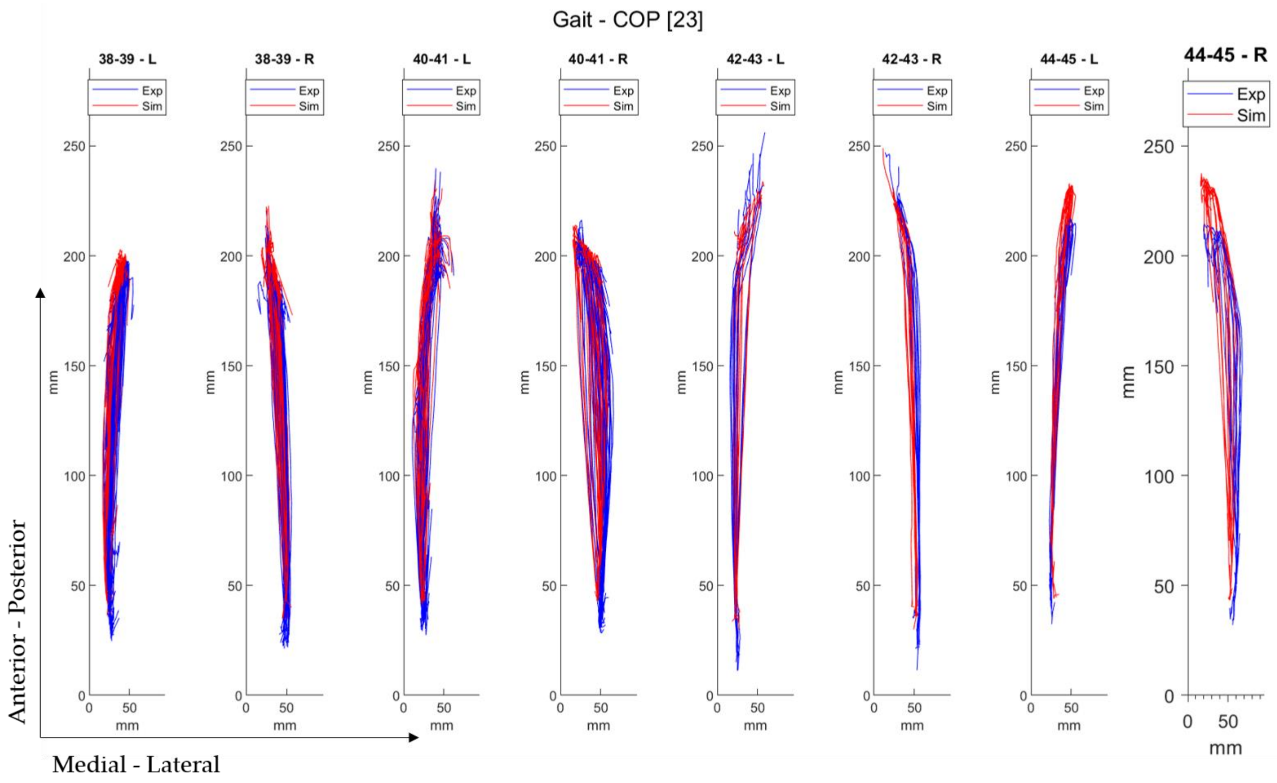

Figure S11: Anterior - Posterior and Medial - Lateral COP, calculated as in [23], Root Mean Square Error (RMSE), in percentage respectively of length insole and of width insole, of each insole size during gait. The insoles were represented respectively: 38-39 in red, 40-41 in blue, 42-43 in green and 44-45 in purple.

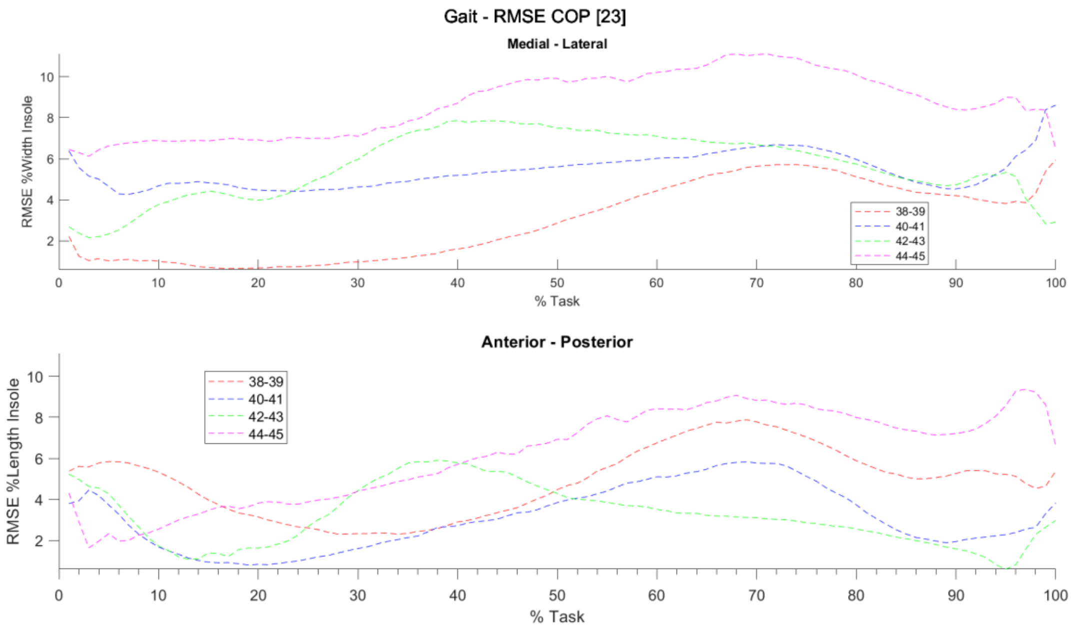

Figure S12: Medial - Lateral and Anterior - Posterior COP, calculated as in [23], Root Mean Square Error (RMSE), in percentage respectively of length insole and of width insole, of each insole size during gait. The insoles were represented respectively: 38-39 in red, 40-41 in blue, 42-43 in green and 44-45 in purple.

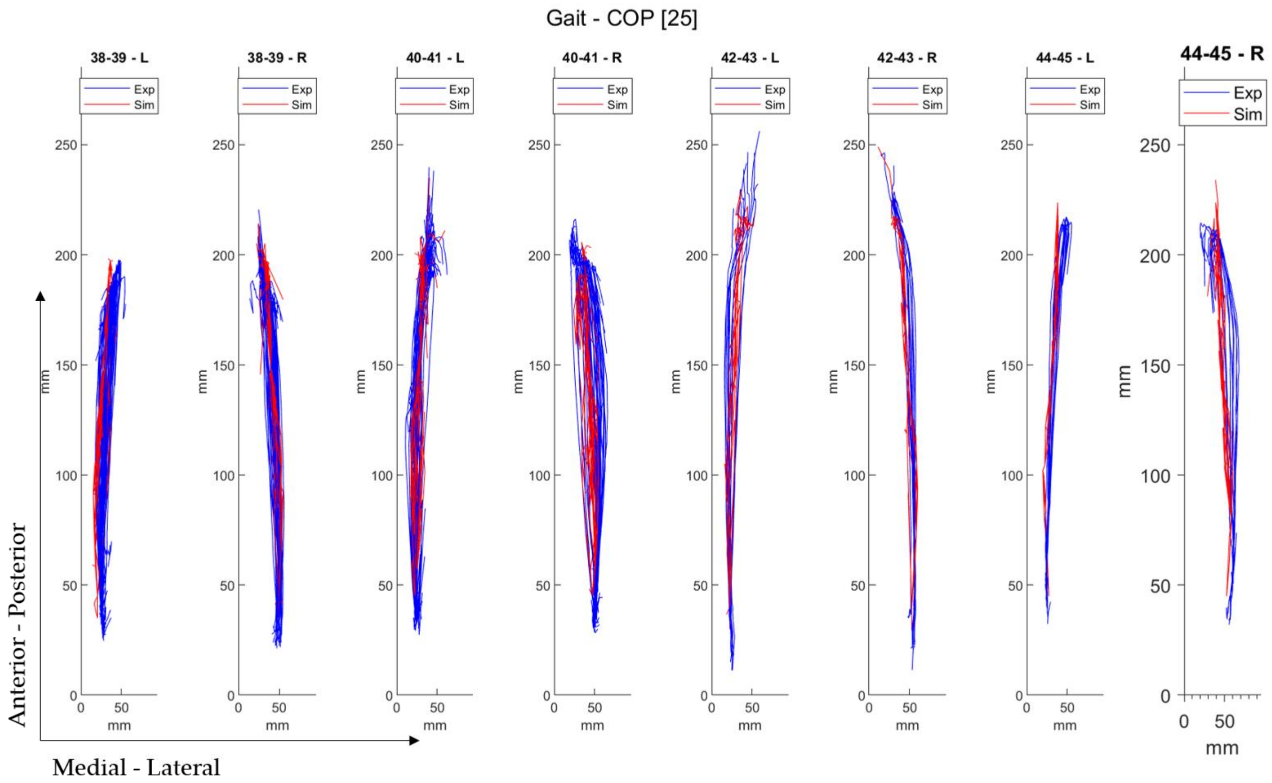

Figure S13: Center of Pressure (COP) of each insole size during gait, calculated as in [25]. Experimental (Exp - Pedar -X® system) in blue and Simulated (Sim - prototype layout) in red. On the x axis the medial lateral axis is represented, while on the y axis the longitudinal (anterior - posterior) axis of the insole is represented.

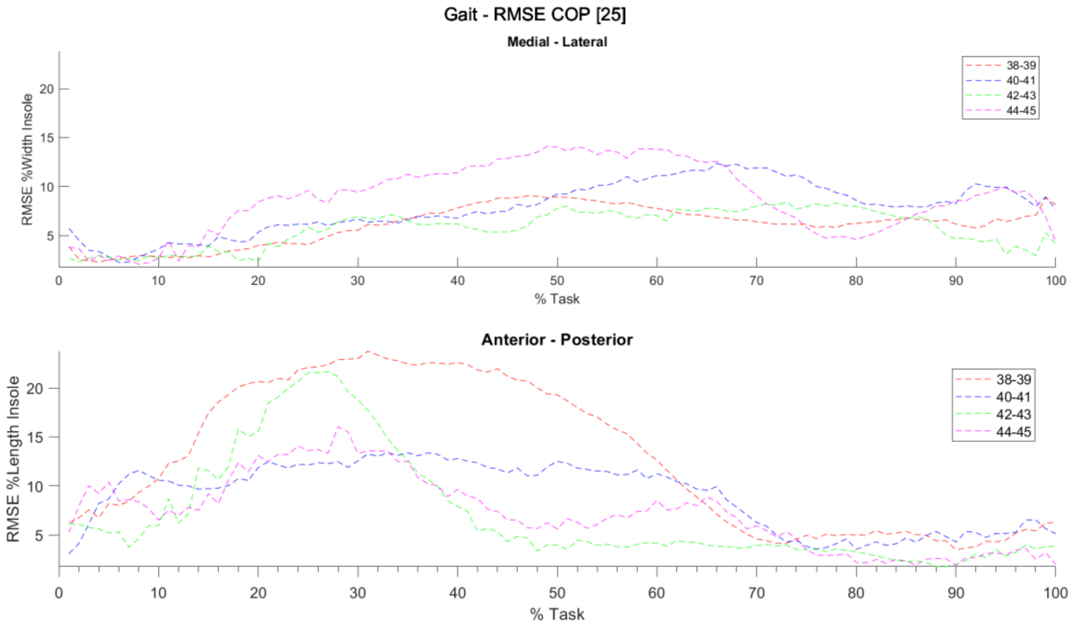

Figure S14: Anterior - Posterior and Medial - Lateral COP, calculated as in [25], Root Mean Square Error (RMSE), in percentage respectively of length insole and of width insole, of each insole size during gait. The insoles were represented respectively: 38-39 in red, 40-41 in blue, 42-43 in green and 44-45 in purple.

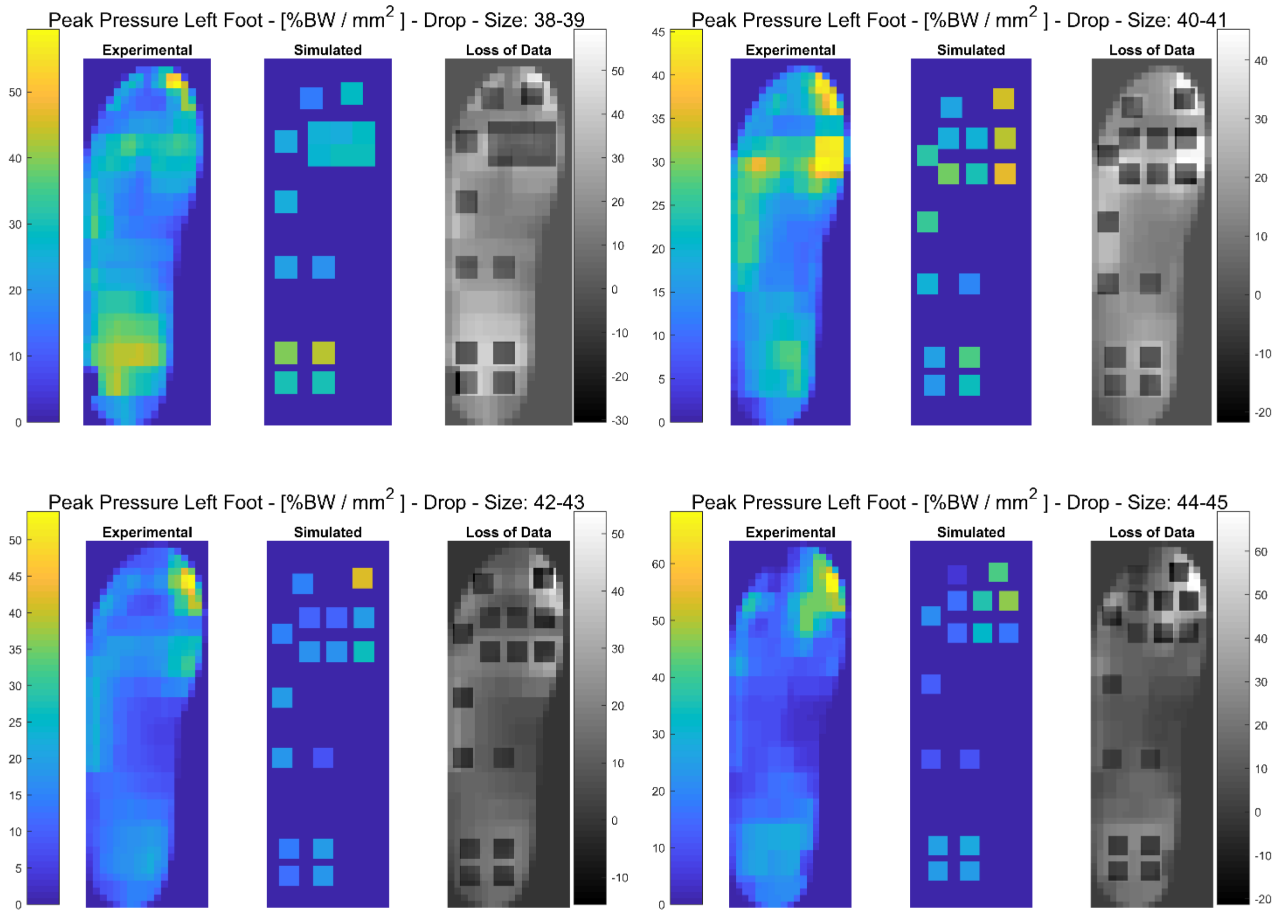

Figure S15: Peak Pressure of left foot for each insole size in %BW/mm² during drop landing. Experimental (Pedar -X® system), simulated (prototype layout) and loss of data (experimental - simulated) footprints were reported. In yellow/white the higher pressure, in blue/black the lower pressure.

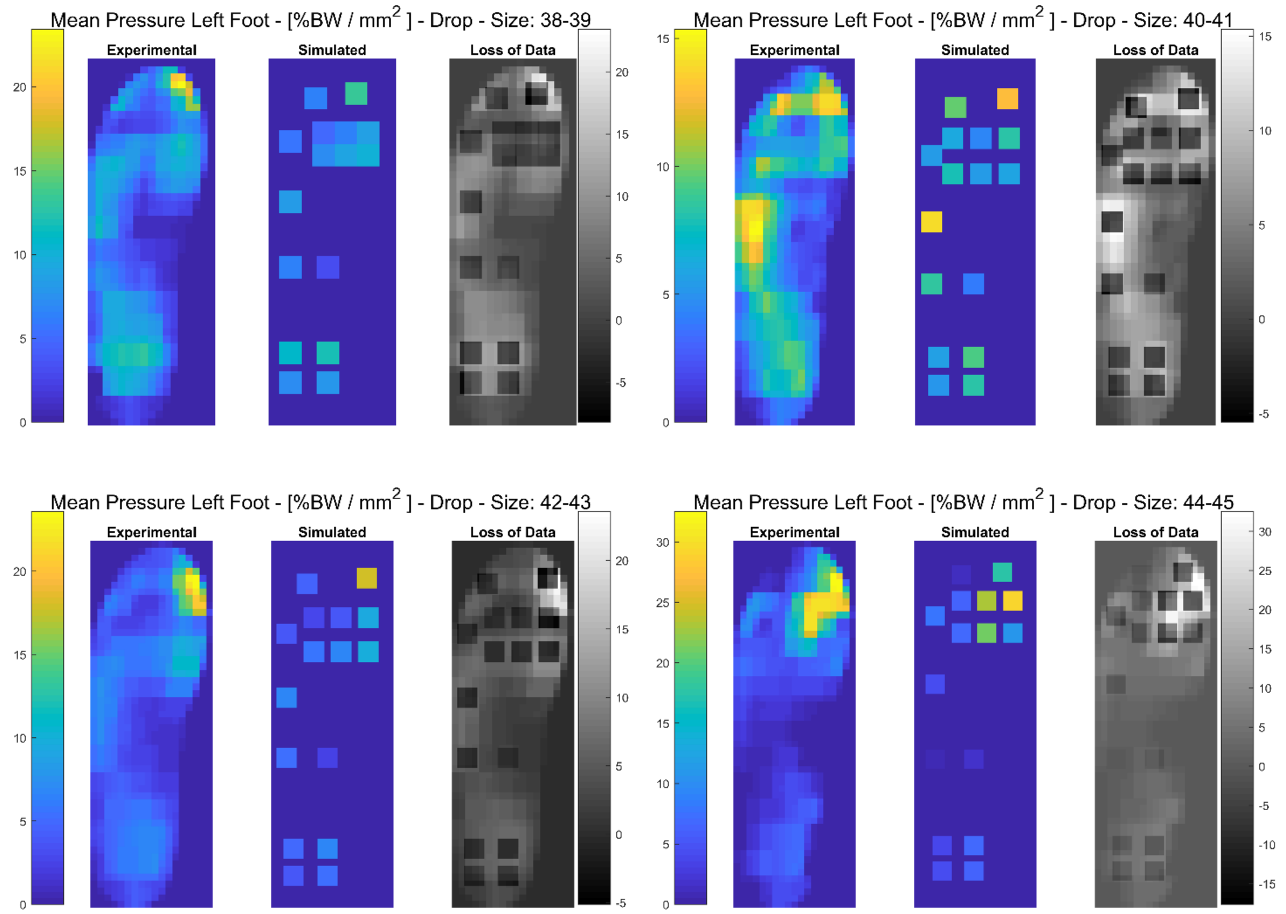

Figure S16: Mean Pressure of left foot for each insole size in %BW/mm^2 during drop landing. Experimental (Pedar -X® system), simulated (prototype layout) and loss of data (experimental - simulated) footprints were reported. In yellow/white the higher pressure, in blue/black the lower pressure.

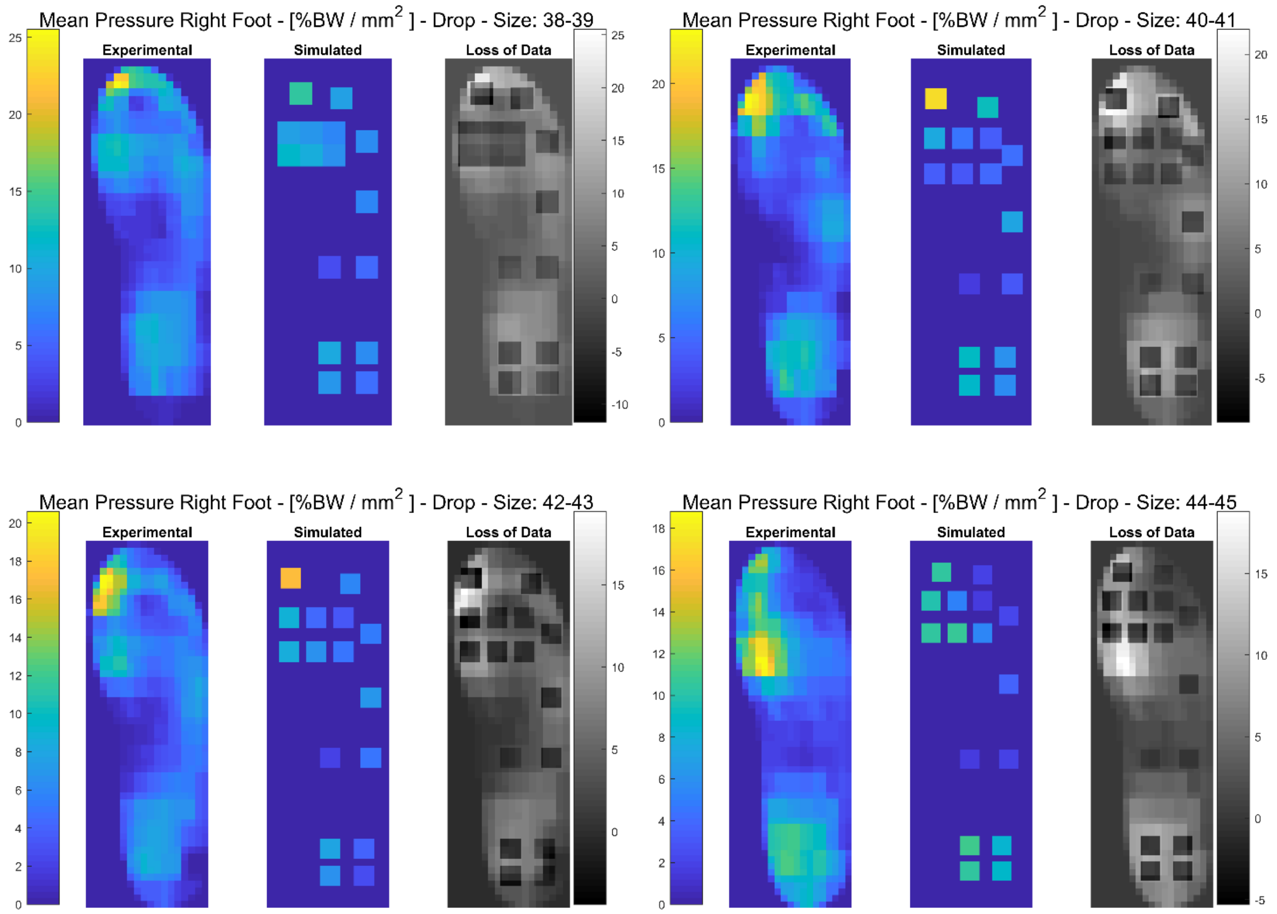

Figure S17: Mean Pressure of right foot for each insole size in %BW/mm² during drop landing. Experimental (Pedar -X® system), simulated (prototype layout) and loss of data (experimental - simulated) footprints were reported. In yellow/white the higher pressure, in blue/black the lower pressure.

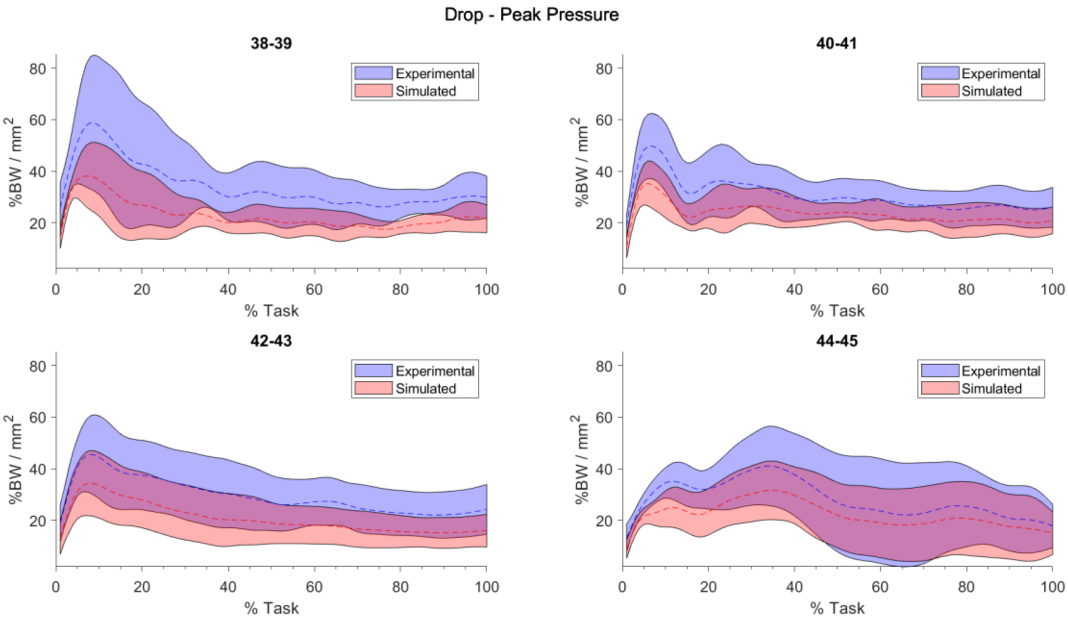

Figure S18: Peak Pressure for each insole size in %BW/mm² during drop landing, expressed in terms of mean and standard deviation. Experimental (Pedar -X® system) in blue and Simulated (prototype layout) in red. Left and right insoles data were averaged.

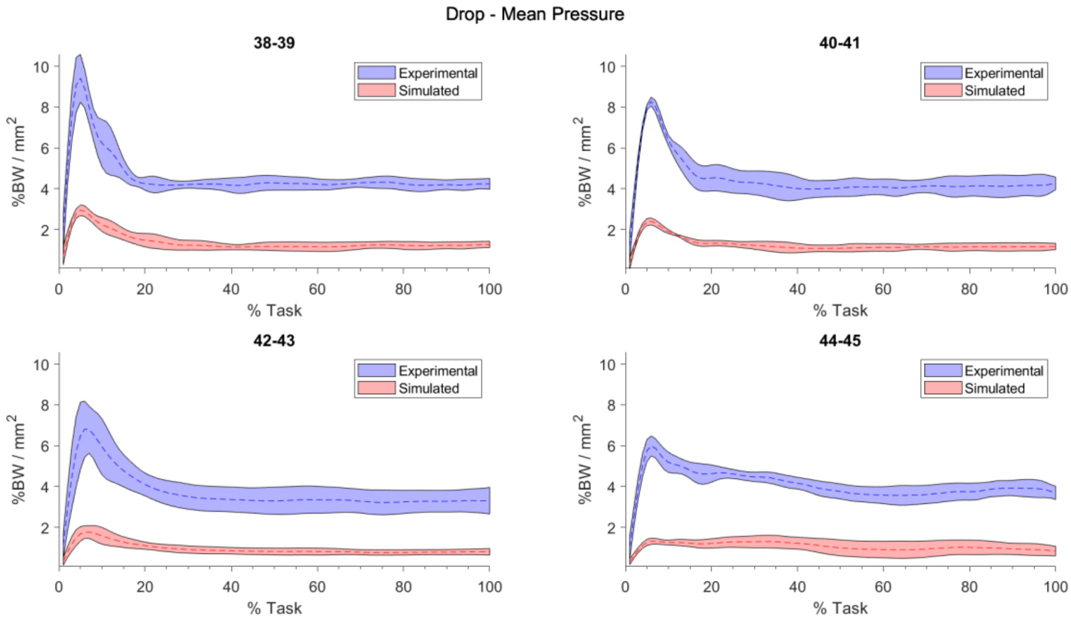

Figure S19: Mean Pressure for each insole size in %BW/mm^2 during drop landing, expressed in terms of mean and standard deviation. Experimental (Pedar -X® system) in blue and Simulated (prototype layout) in red. Left and right insoles data were averaged.

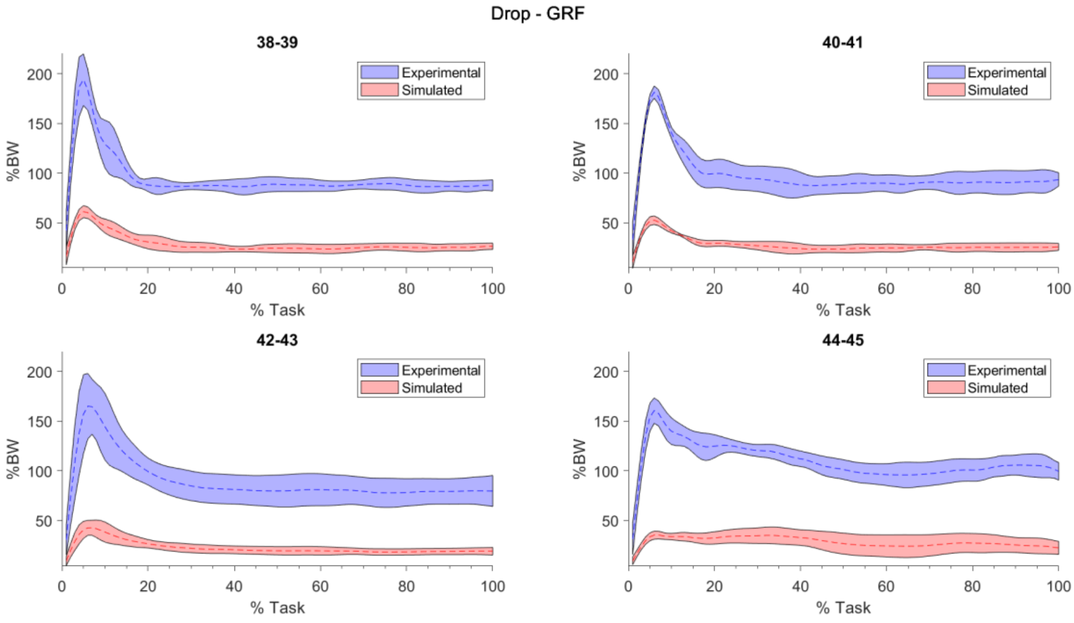

Figure S20: Ground Reaction Force (GRF) for each insole size in percentage of body weight during drop landing, expressed in terms of mean and standard deviation. Experimental (Pedar -X® system) in blue and Simulated (prototype layout) in red. Left and right insoles data were averaged.

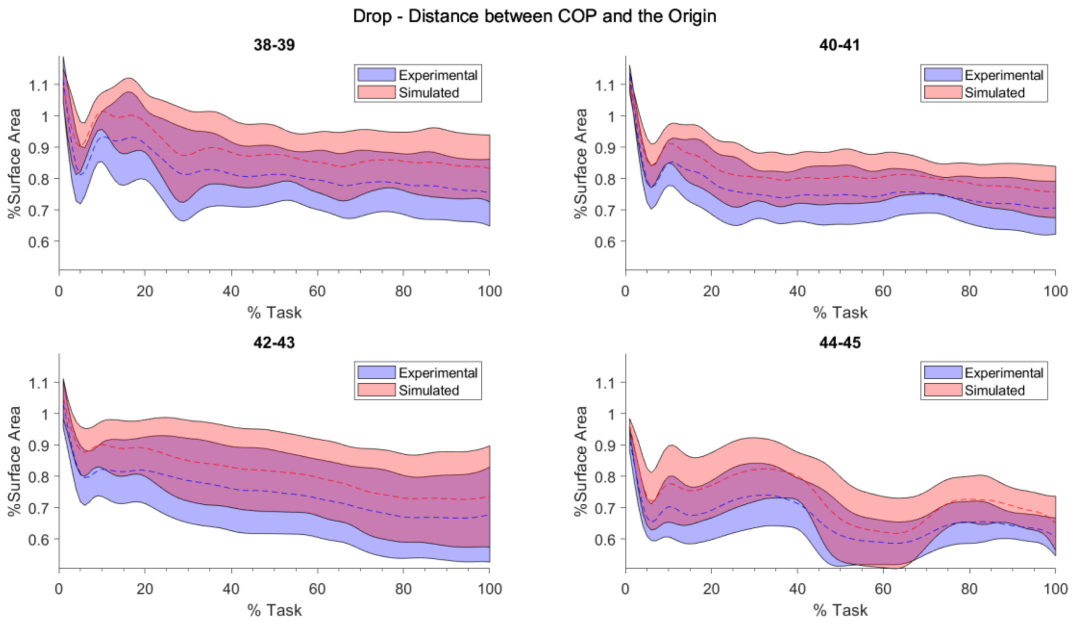

Figure S21: Distance between Center of Pressure (COP) and the Origin for each insole size in percentage of surface area during drop landing, expressed in terms of mean and standard deviation. Experimental (Pedar - X® system) in blue and Simulated (prototype layout) in red. Left and right insoles data were averaged.

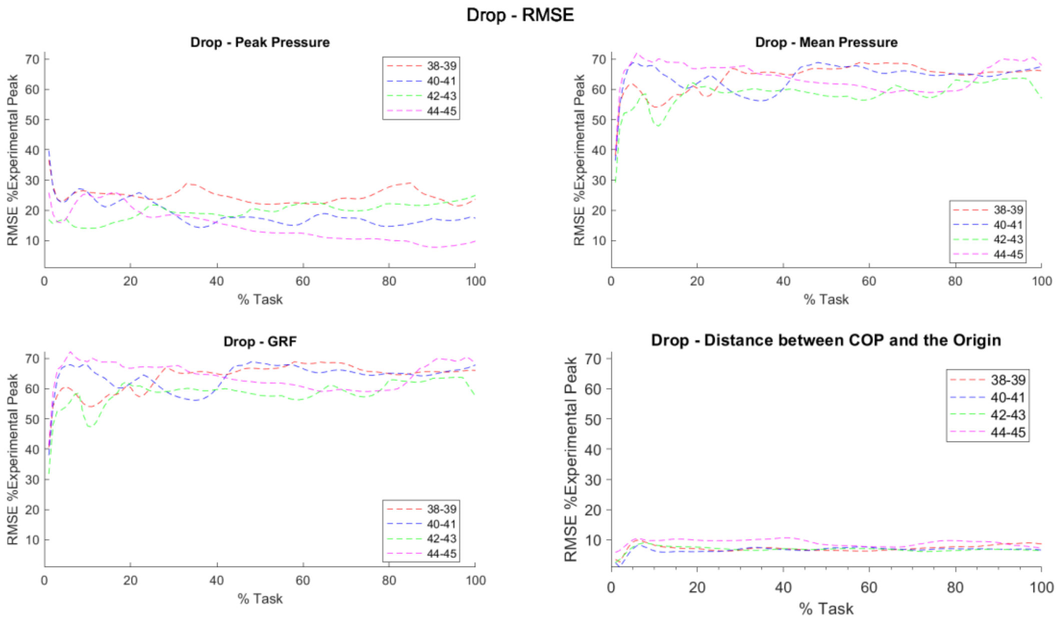

Figure S22: Root Mean Square Error (RMSE) in percentage of Experimental Peak (Pedar -X® system) for each variable and for each insole size during drop landing. The insoles size were represented respectively: 38-39 in red, 40-41 in blue, 42-43 in green and 44-45 in purple.

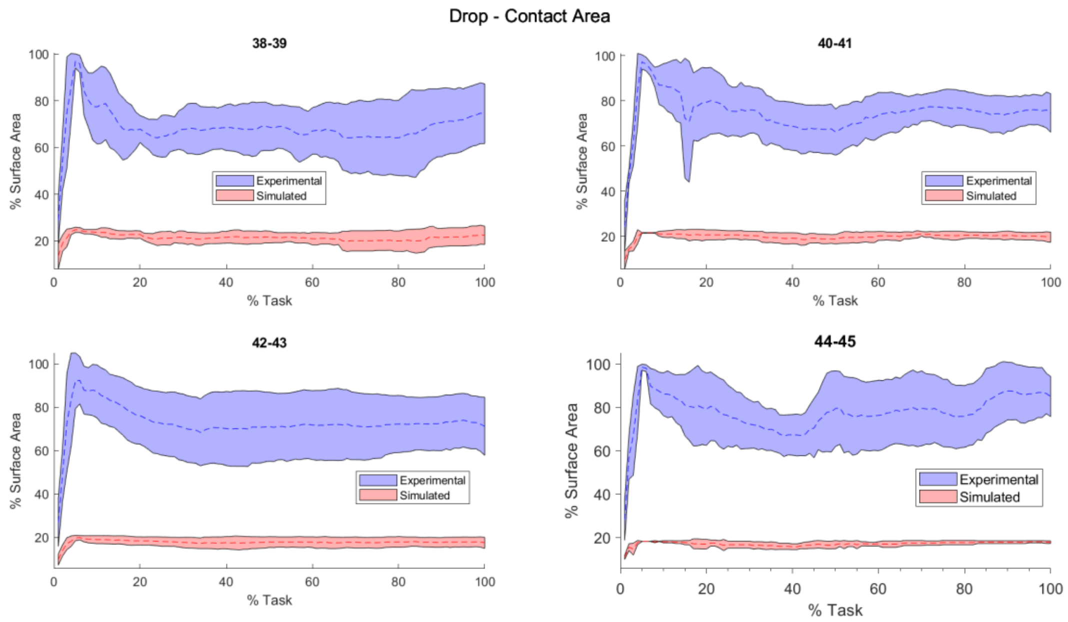

Figure S23: Contact Area for each insole size in percentage of surface area during drop landing, expressed in terms of mean and standard deviation. Experimental (Pedar -X® system) in blue and Simulated (prototype layout) in red. Left and right insoles data were averaged.

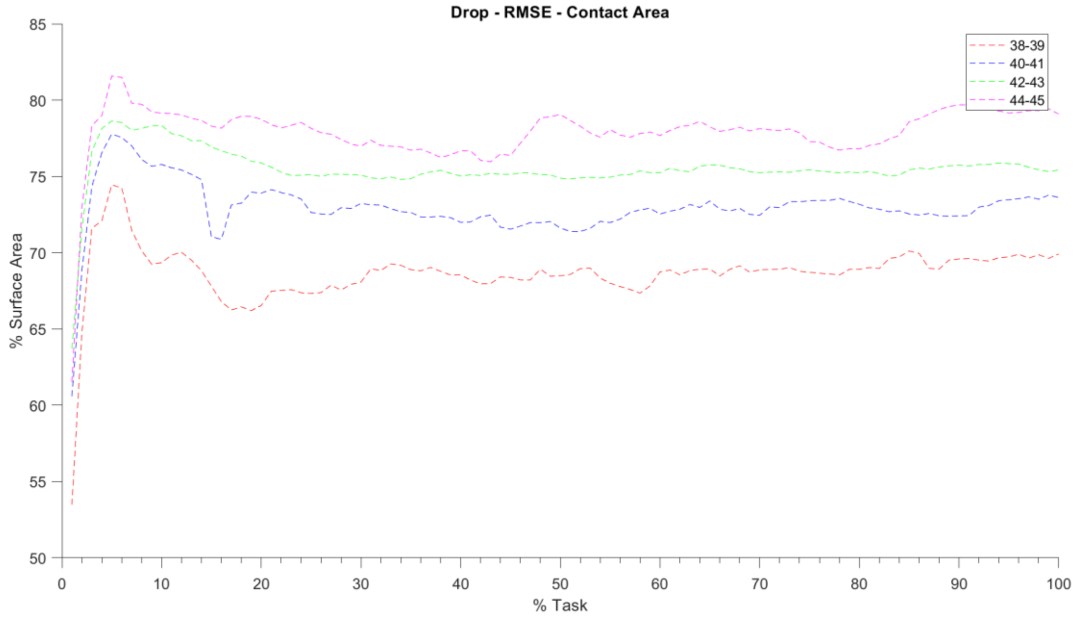

Figure S24: Contact Area Root Mean Square Error (RMSE) in percentage of Surface area for each insole size during drop landing. The insoles were represented respectively: 38-39 in red, 40-41 in blue, 42-43 in green and 44-45 in purple.

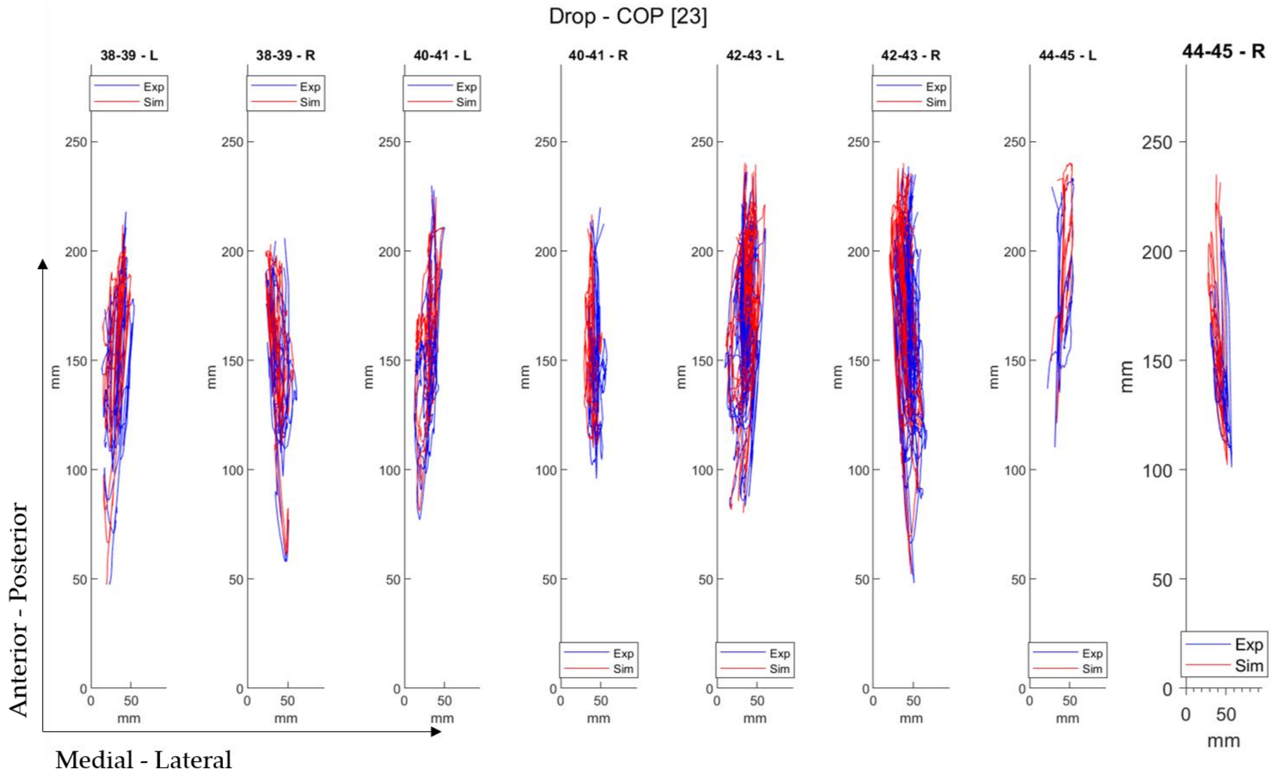

Figure S25: Anterior - Posterior and Medial - Lateral COP, calculated as in [23], Root Mean Square Error (RMSE), in percentage respectively of length insole and of width insole, of each insole size during drop landing. The insoles were represented respectively: 38-39 in red, 40-41 in blue, 42-43 in green and 44-45 in purple.

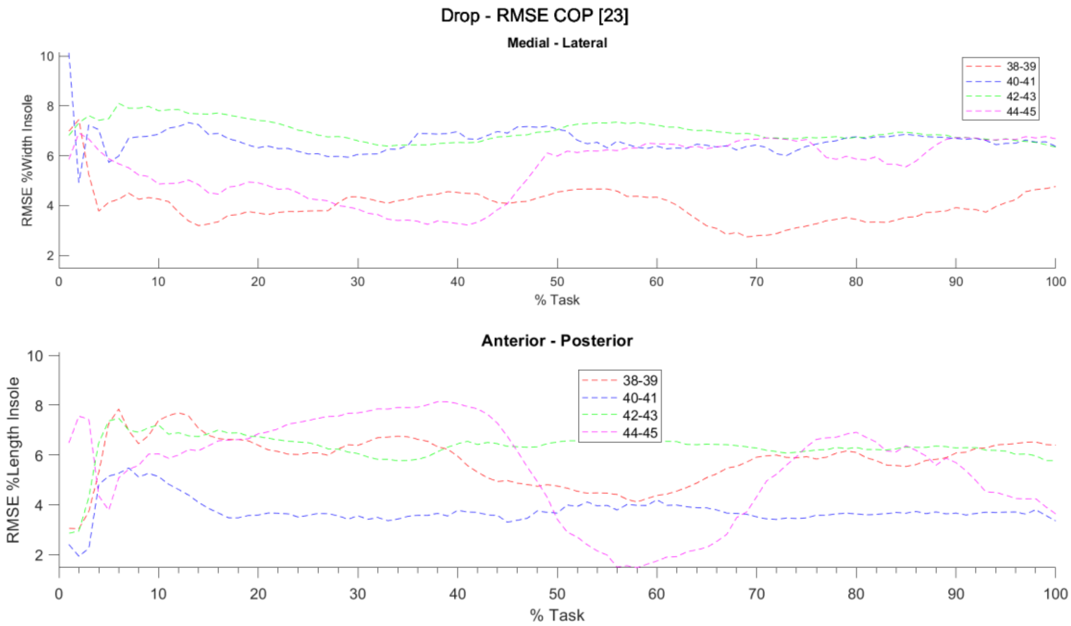

Figure S26: Medial - Lateral and Anterior - Posterior COP, calculated as in [23], Root Mean Square Error (RMSE), in percentage respectively of length insole and of width insole, of each insole size during drop landing. The insoles were represented respectively: 38-39 in red, 40-41 in blue, 42-43 in green and 44-45 in purple.

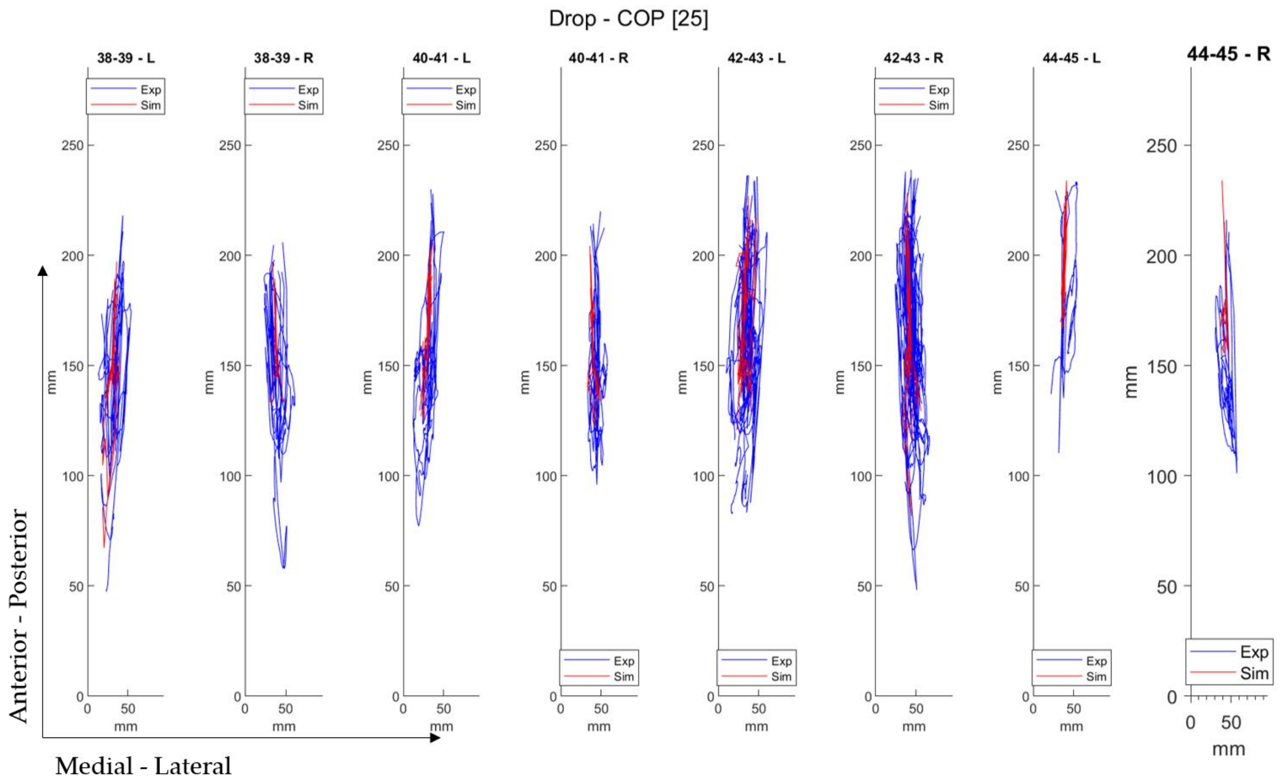

Figure S27: Center of Pressure (COP) of each insole size during drop landing, calculated as in [25]. Experimental (Exp - Pedar -X® system) in blue and Simulated (Sim - prototype layout) in red. On the x axis the medial lateral axis is represented, while on the y axis the longitudinal (anterior - posterior) axis of the insole is represented.

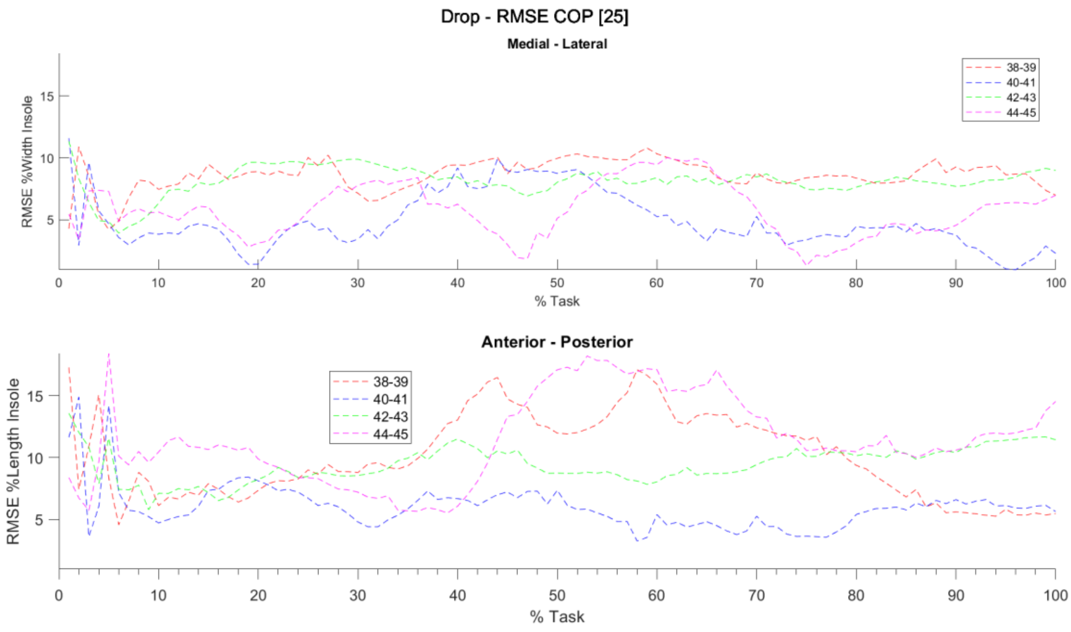

Figure S28: Anterior - Posterior and Medial - Lateral COP, calculated as in [25], Root Mean Square Error (RMSE), in percentage respectively of length insole and of width insole, of each insole size during drop landing. The insoles were represented respectively: 38-39 in red, 40-41 in blue, 42-43 in green and 44-45 in purple.

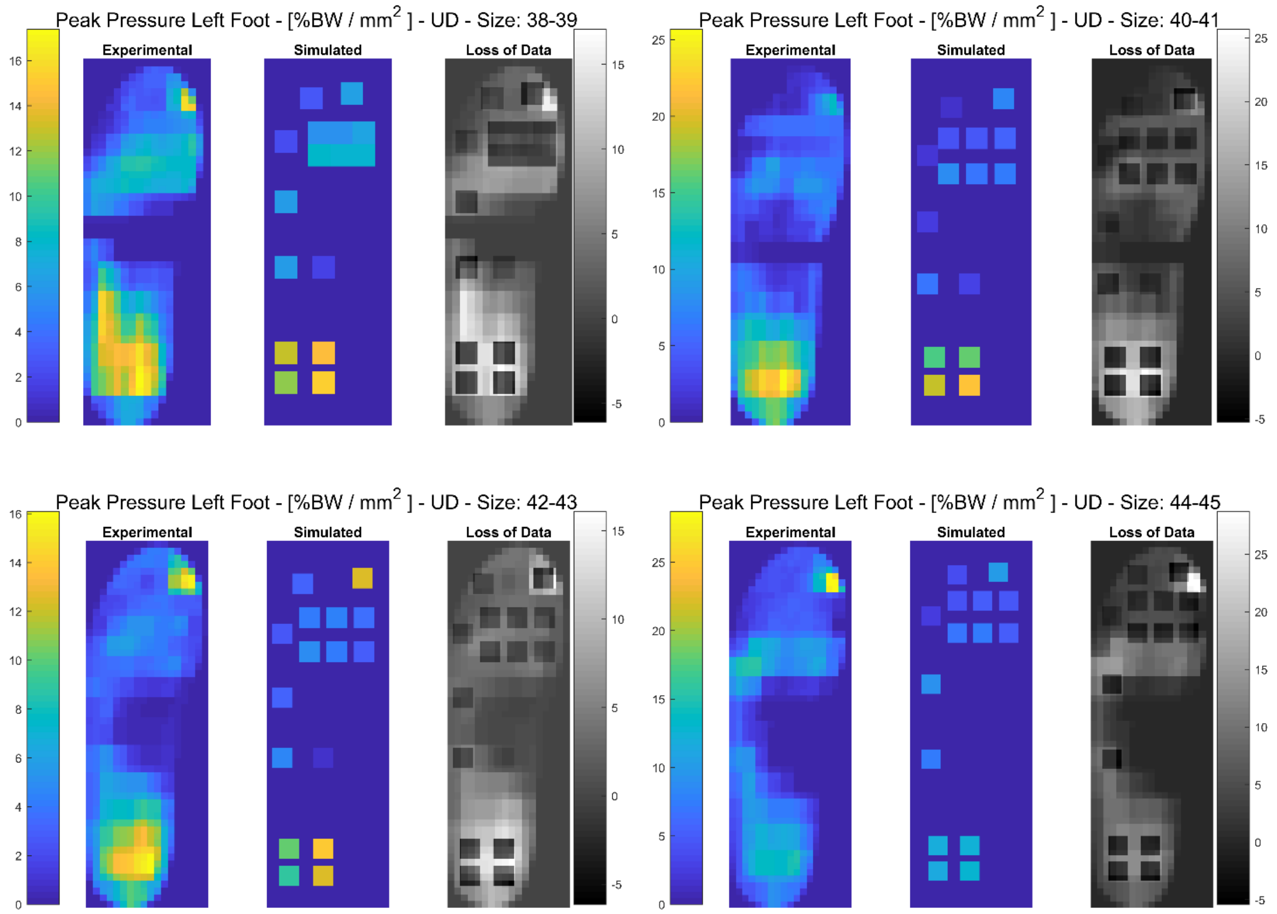

Figure S29: Peak Pressure of left foot for each insole size in %BW/mm² during unloaded descending. Experimental (Pedar -X® system), simulated (prototype layout) and loss of data (experimental - simulated) footprints were reported. In yellow/white the higher pressure, in blue/black the lower pressure.

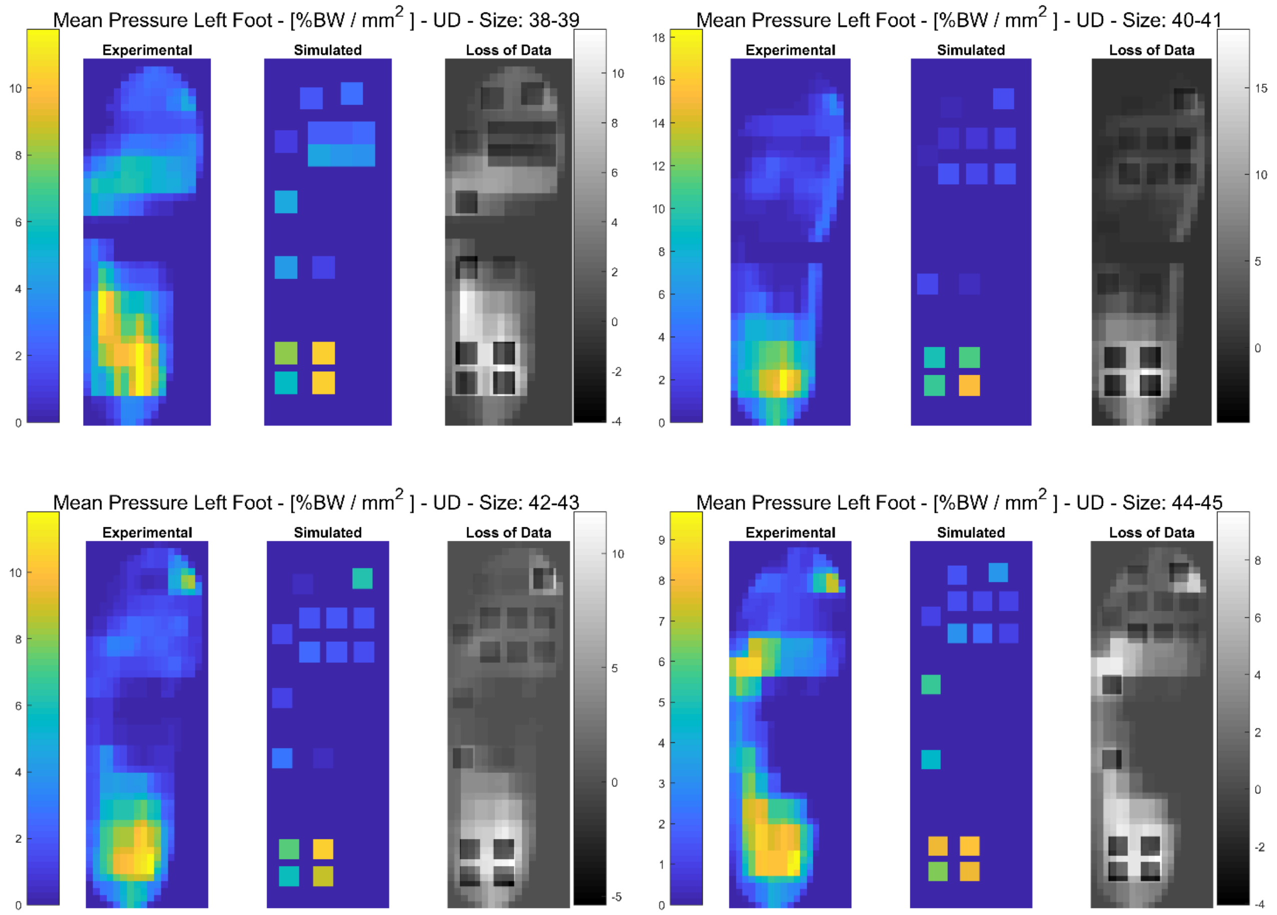

Figure S30: Mean Pressure of left foot for each insole size in %BW/mm^2 during unloaded descending. Experimental (Pedar -X® system), simulated (prototype layout) and loss of data (experimental - simulated) footprints were reported. In yellow/white the higher pressure, in blue/black the lower pressure.

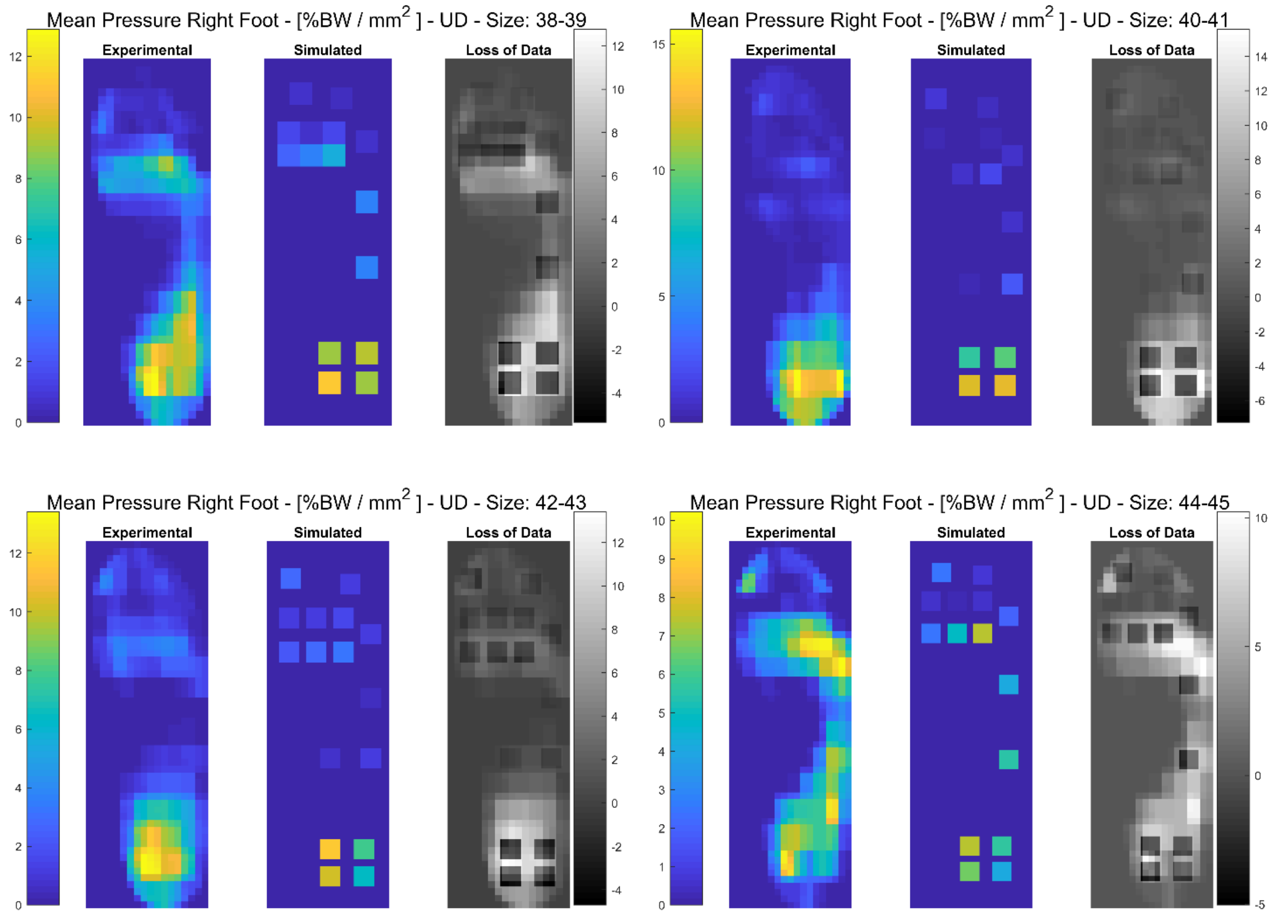

Figure S31: Mean Pressure of right foot for each insole size in %BW/mm² during unloaded descending. Experimental (Pedar -X® system), simulated (prototype layout) and loss of data (experimental - simulated) footprints were reported. In yellow/white the higher pressure, in blue/black the lower pressure.

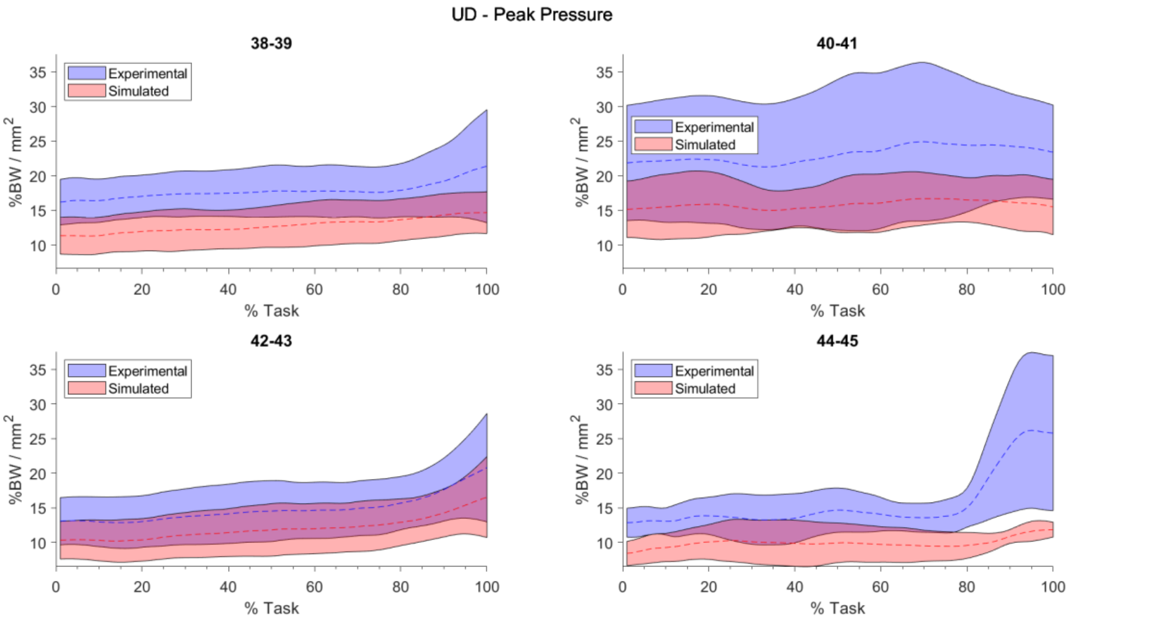

Figure S32: Peak Pressure for each insole size in %BW/mm² during unloaded descending, expressed in terms of mean and standard deviation. Experimental (Pedar -X® system) in blue and Simulated (prototype layout) in red. Left and right insoles data were averaged.

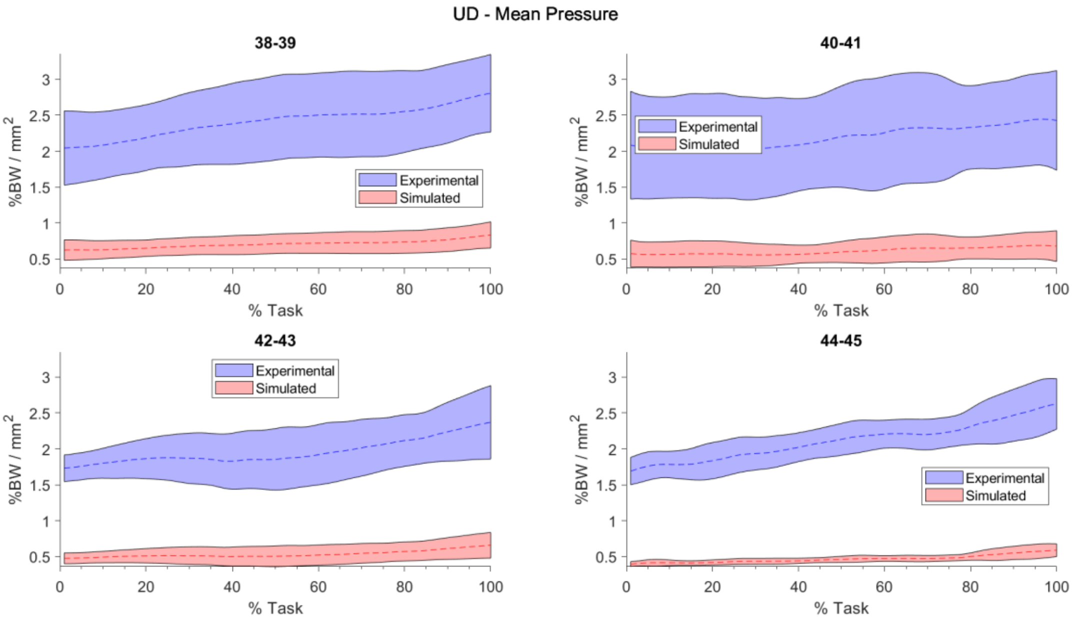

Figure S33: Mean Pressure for each insole size in %BW/mm^2 during unloaded descending, expressed in terms of mean and standard deviation. Experimental (Pedar -X® system) in blue and Simulated (prototype layout) in red. Left and right insoles data were averaged.

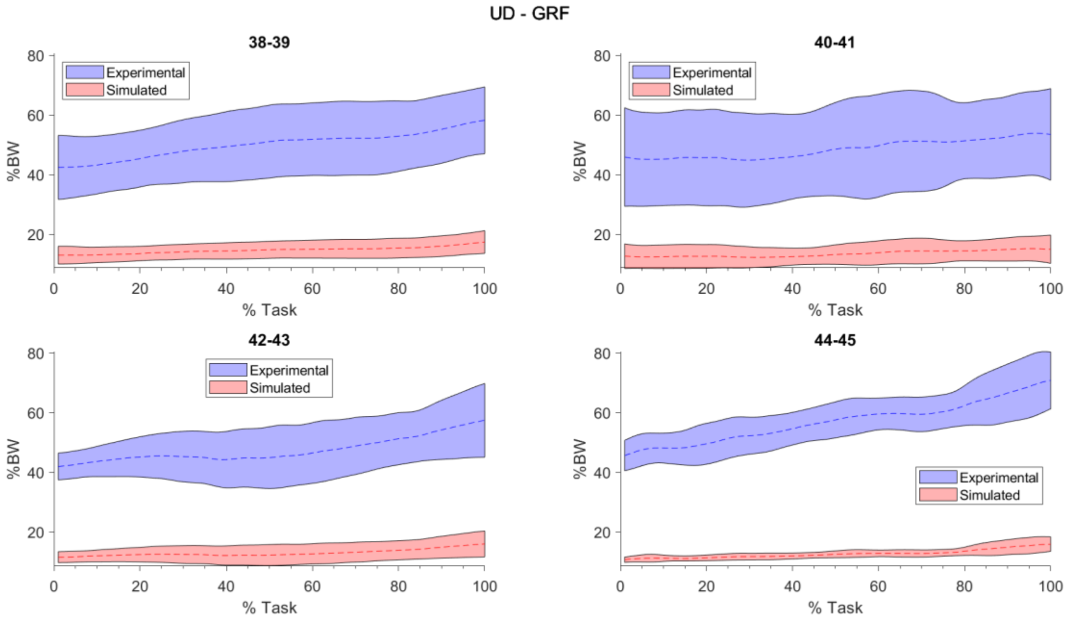

Figure S34: Ground Reaction Force (GRF) for each insole size in percentage of body weight during unloaded descending, expressed in terms of mean and standard deviation. Experimental (Pedar -X® system) in blue and Simulated (prototype layout) in red. Left and right insoles data were averaged.

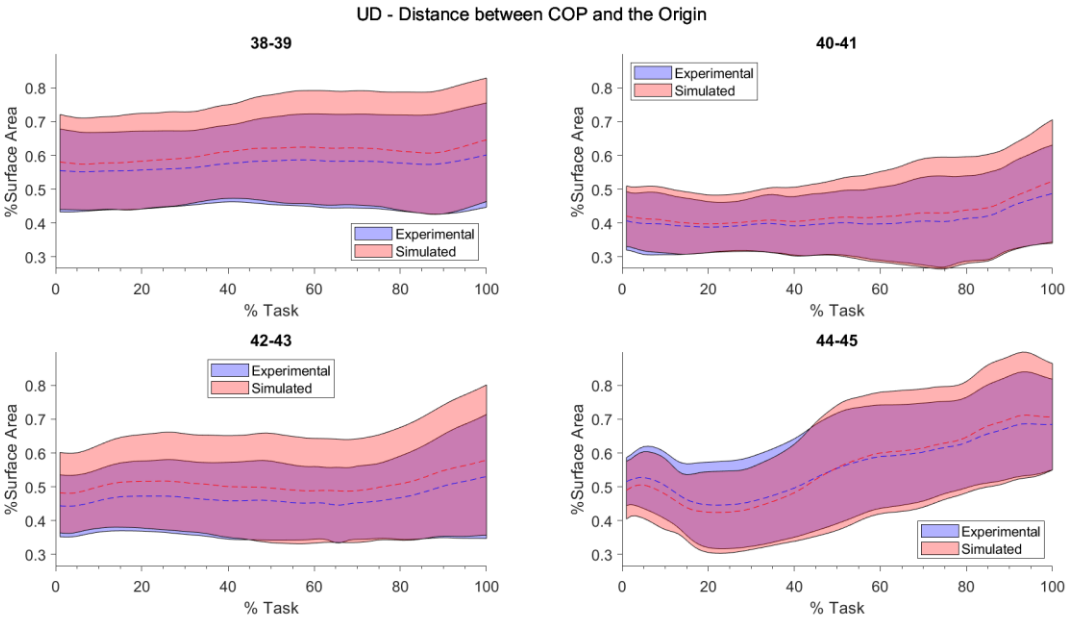

Figure S35: Distance between Center of Pressure (COP) and the Origin for each insole size in percentage of surface area during unloaded descending, expressed in terms of mean and standard deviation. Experimental (Pedar -X® system) in blue and Simulated (prototype layout) in red. Left and right insoles data were averaged.

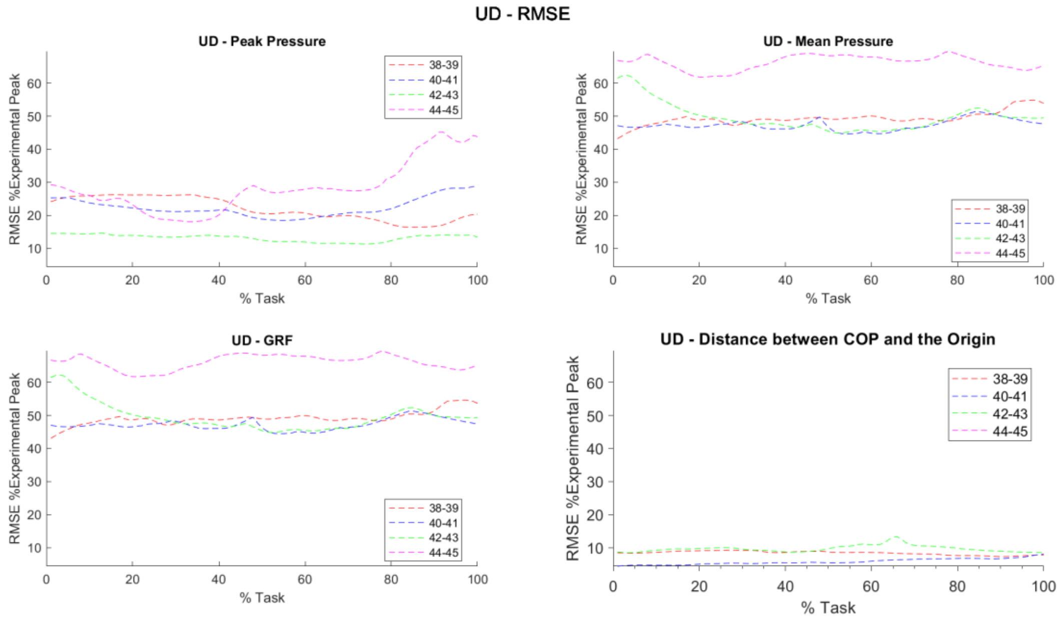

Figure S36: Root Mean Square Error (RMSE) in percentage of Experimental Peak (Pedar -X® system) for each variable and for each insole size during unloaded descending. The insoles were represented respectively: 38-39 in red, 40-41 in blue, 42-43 in green and 44-45 in purple.

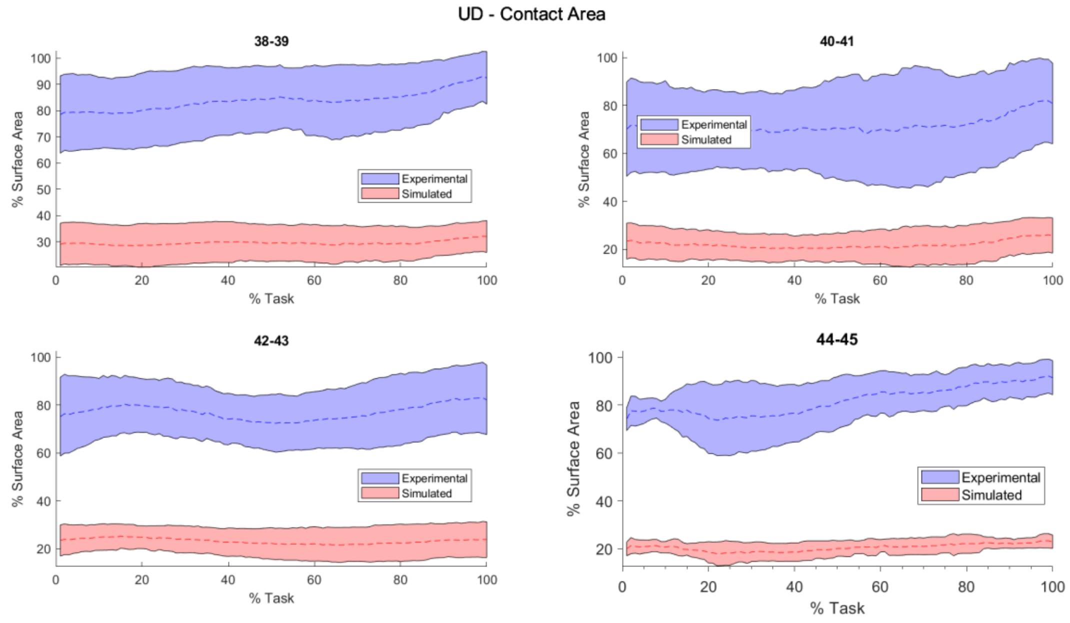

Figure S37: Contact Area for each insole size in percentage of surface area during unloaded descending, expressed in terms of mean and standard deviation. Experimental (Pedar -X® system) in blue and Simulated (prototype layout) in red. Left and right insoles data were averaged.

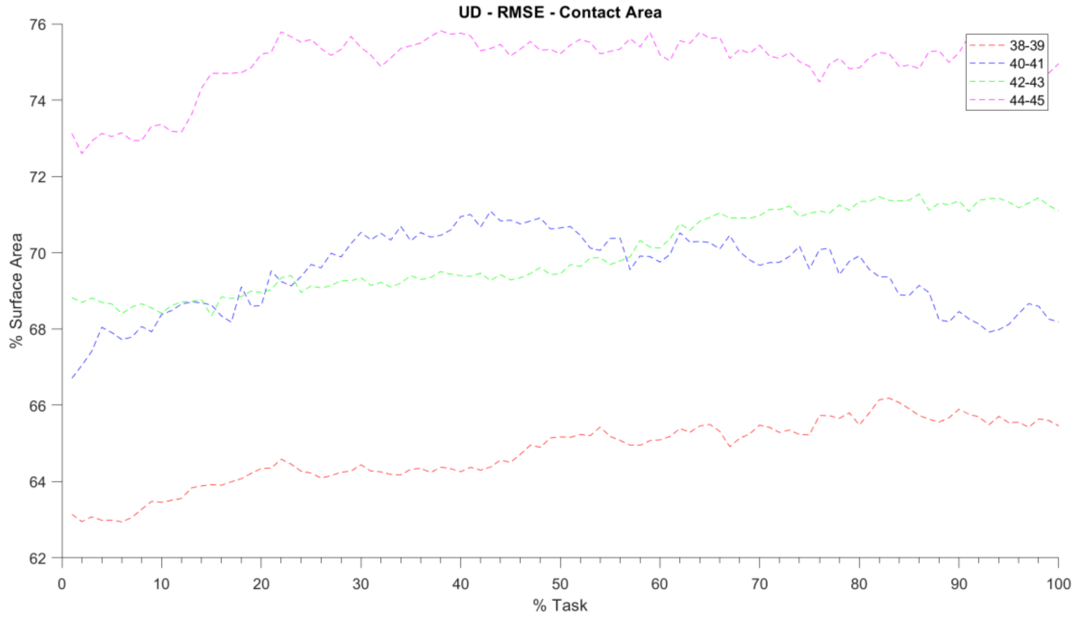

Figure S38: Contact Area Root Mean Square Error (RMSE) in percentage of Surface area for each insole size during unloaded descending. The insoles were represented respectively: 38-39 in red, 40-41 in blue, 42-43 in green and 44-45 in purple.

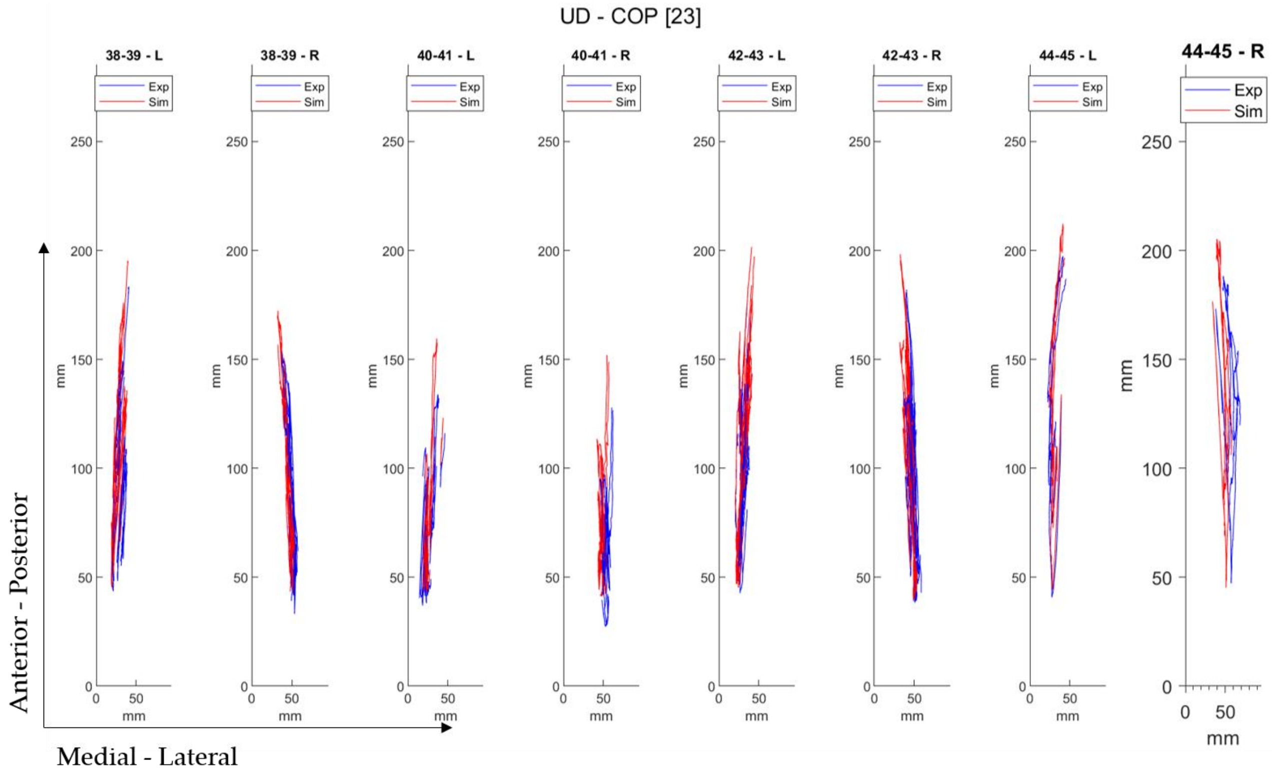

Figure S39: Anterior - Posterior and Medial - Lateral COP, calculated as in [23], Root Mean Square Error (RMSE), in percentage respectively of length insole and of width insole, of each insole size during unloaded descending. The insoles were represented respectively: 38-39 in red, 40-41 in blue, 42-43 in green and 44-45 in purple.

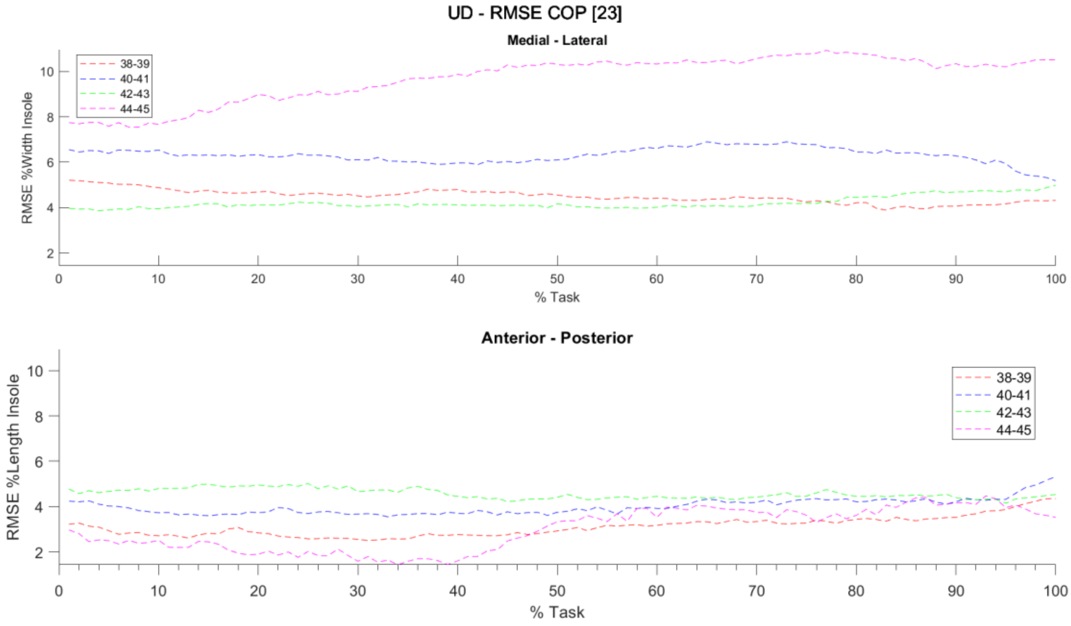

Figure S40: Medial - Lateral and Anterior - Posterior COP, calculated as in [23], Root Mean Square Error (RMSE), in percentage respectively of length insole and of width insole, of each insole size during unloaded descending. The insoles were represented respectively: 38-39 in red, 40-41 in blue, 42-43 in green and 44-45 in purple.

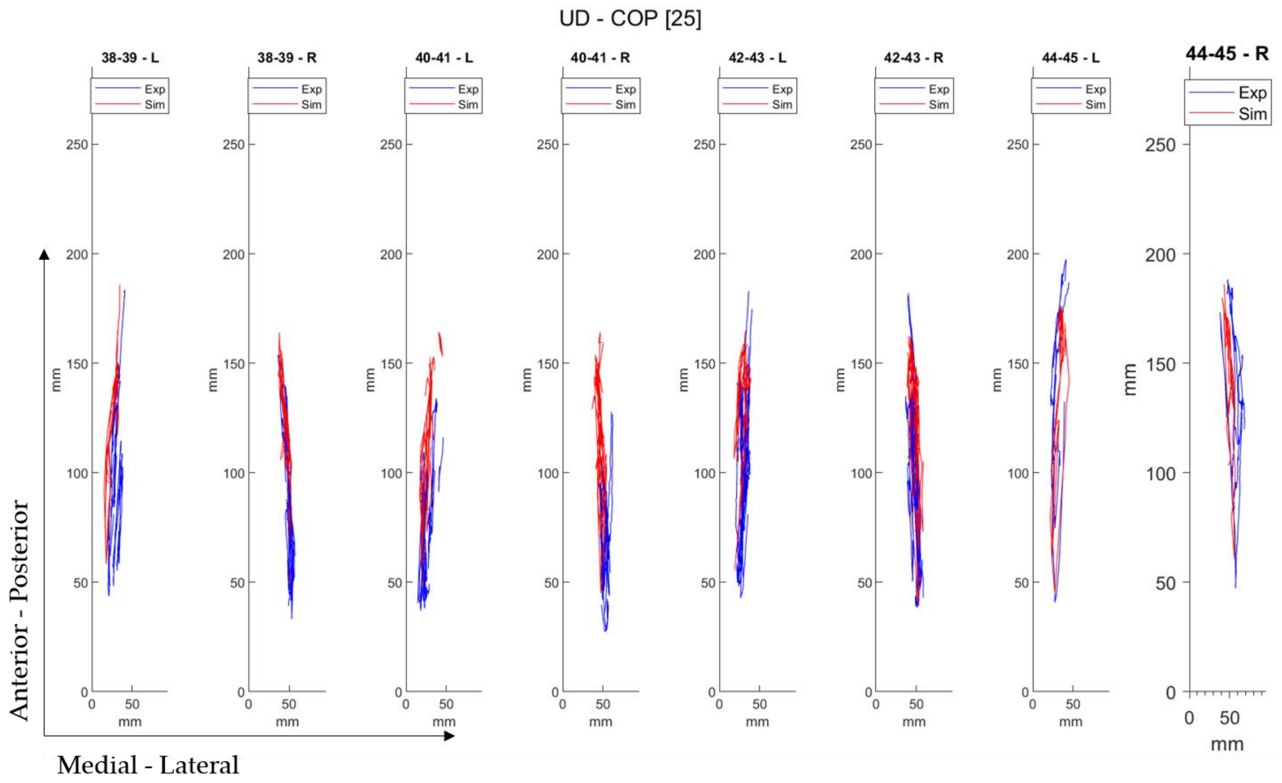

Figure S41: Center of Pressure (COP) of each insole size during unloaded descending, calculated as in [25]. Experimental (Exp - Pedar -X® system) in blue and Simulated (Sim - prototype layout) in red. On the x axis the medial lateral axis is represented, while on the y axis the longitudinal (anterior - posterior) axis of the insole is represented.

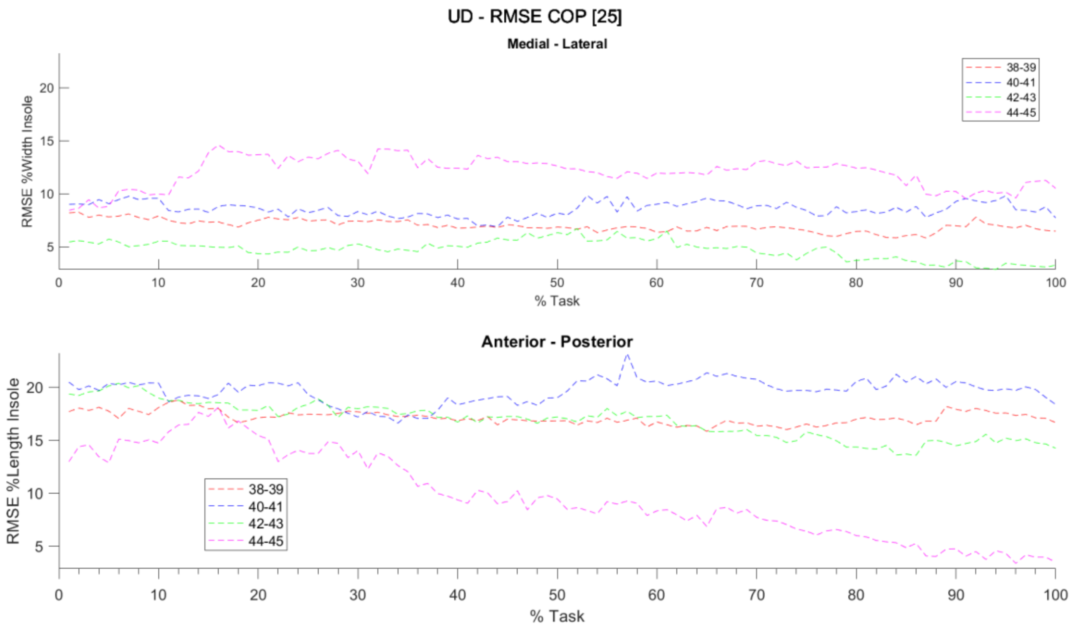

Figure S42: Anterior - Posterior and Medial - Lateral COP, calculated as in [25], Root Mean Square Error (RMSE), in percentage respectively of length insole and of width insole, of each insole size during unloaded descending. The insoles were represented respectively: 38-39 in red, 40-41 in blue, 42-43 in green and 44-45 in purple.

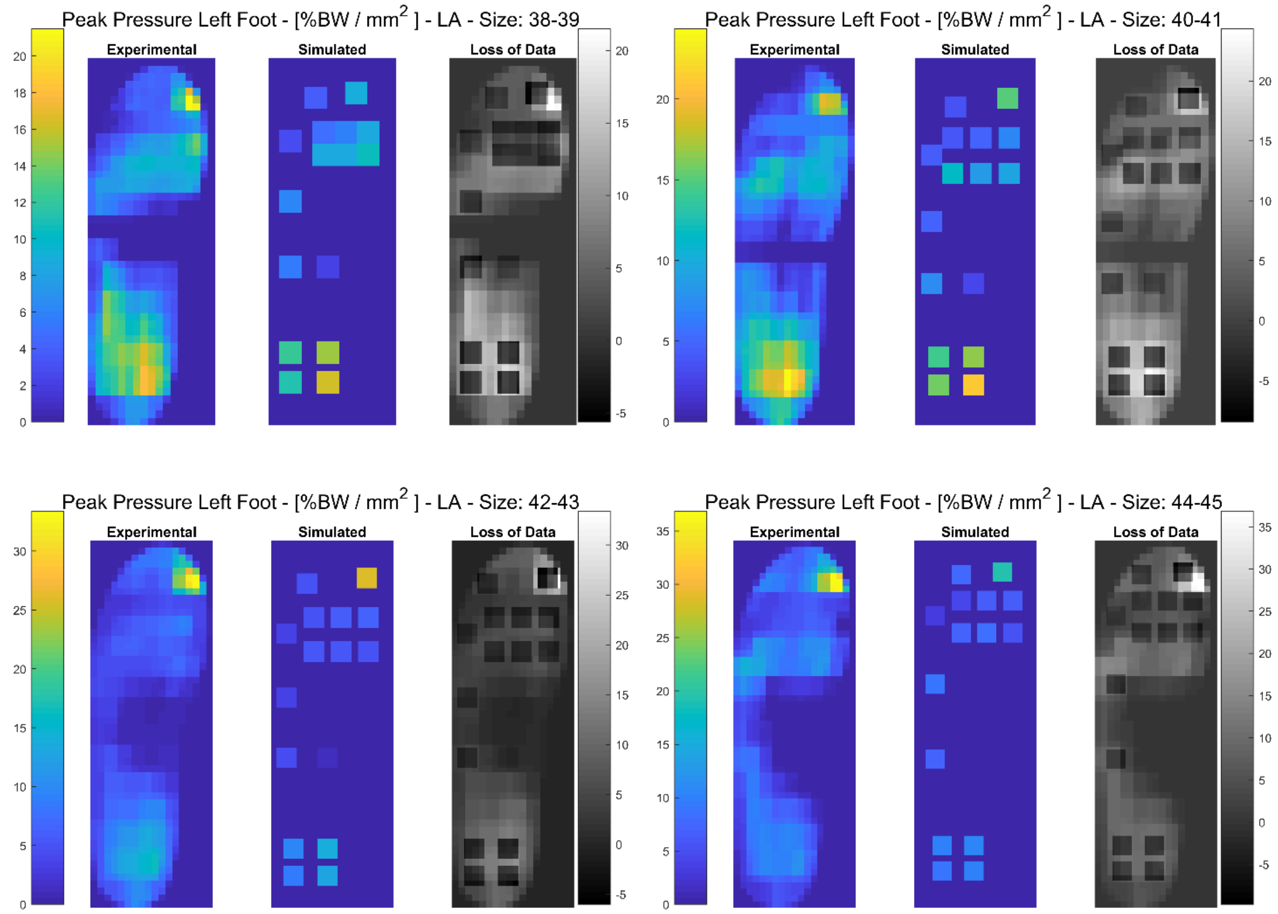

Figure S43: Peak Pressure of left foot for each insole size in %BW/mm² during loaded ascending. Experimental (Pedar -X® system), simulated (prototype layout) and loss of data (experimental - simulated) footprints were reported. In yellow/white the higher pressure, in blue/black the lower pressure.

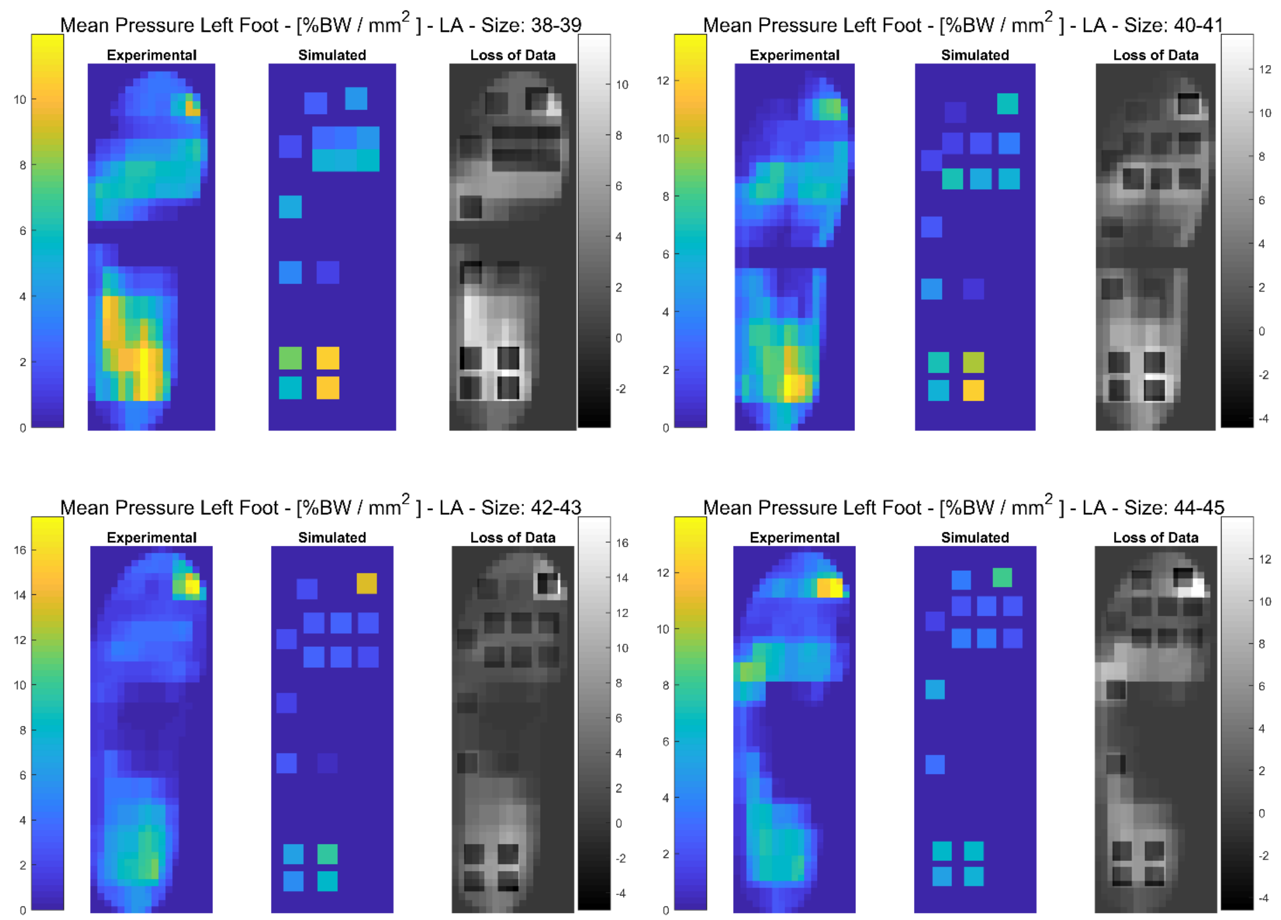

Figure S44: Mean Pressure of left foot for each insole size in %BW/mm² during loaded ascending. Experimental (Pedar -X® system), simulated (prototype layout) and loss of data (experimental - simulated) footprints were reported. In yellow/white the higher pressure, in blue/black the lower pressure.

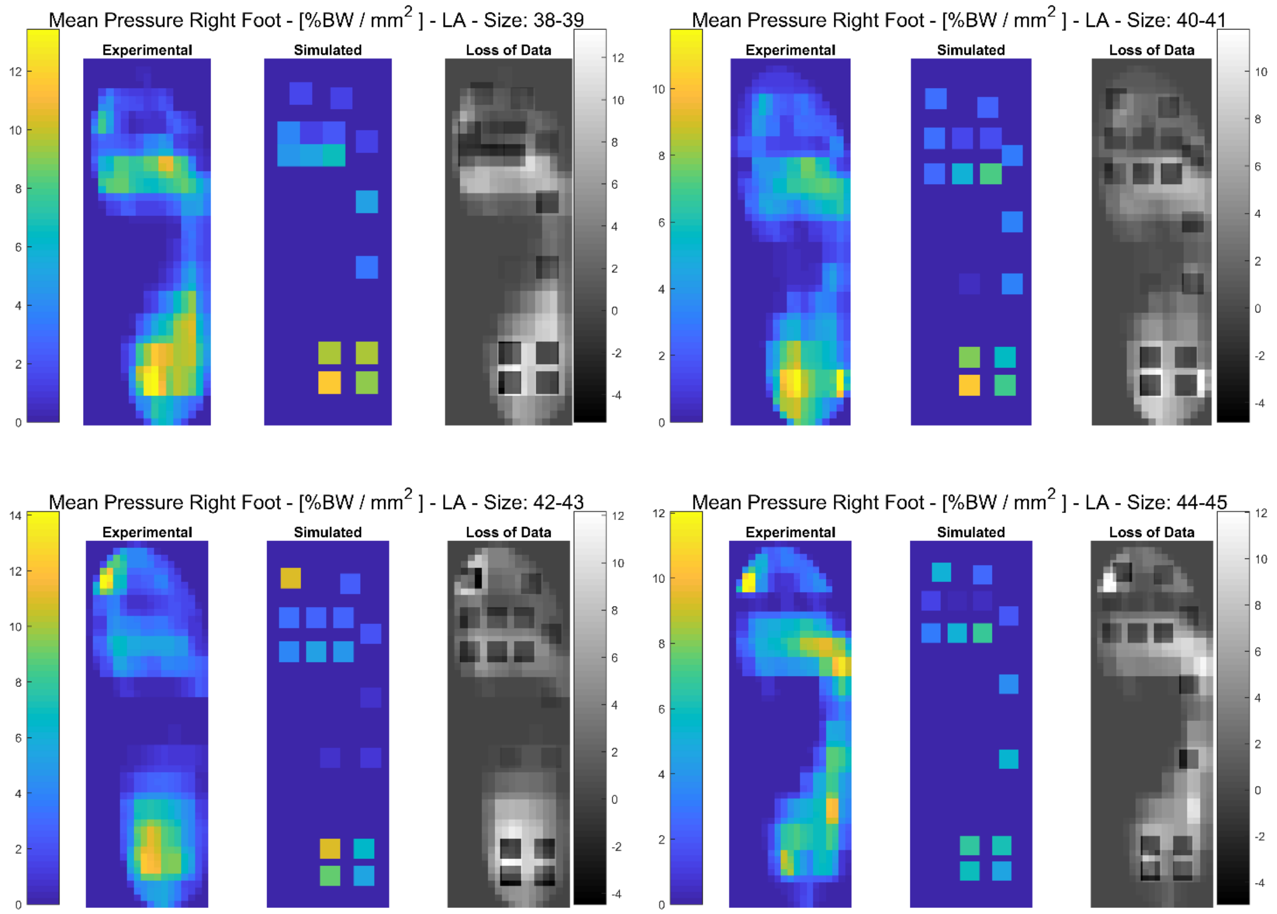

Figure S45: Mean Pressure of right foot for each insole size in %BW/mm² during loaded ascending. Experimental (Pedar -X® system), simulated (prototype layout) and loss of data (experimental - simulated) footprints were reported. In yellow/white the higher pressure, in blue/black the lower pressure.

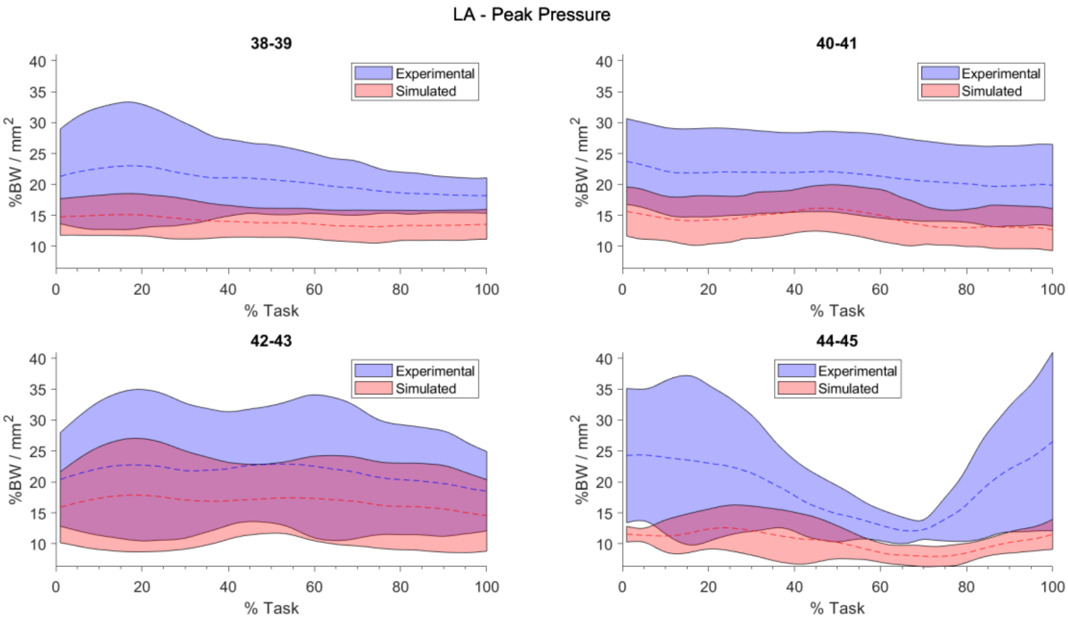

Figure S46: Peak Pressure for each insole size in %BW/mm² during loaded ascending, expressed in terms of mean and standard deviation. Experimental (Pedar -X® system) in blue and Simulated (prototype layout) in red. Left and right insoles data were averaged.

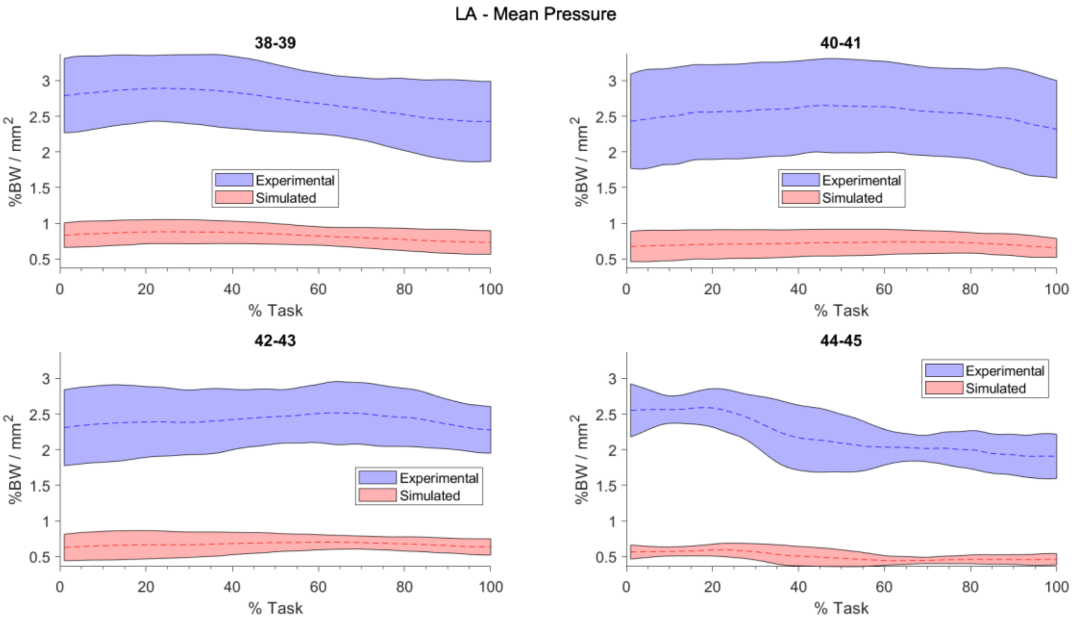

Figure S47: Mean Pressure for each insole size in %BW/mm² during loaded ascending, expressed in terms of mean and standard deviation. Experimental (Pedar -X® system) in blue and Simulated (prototype layout) in red. Left and right insoles data were averaged.

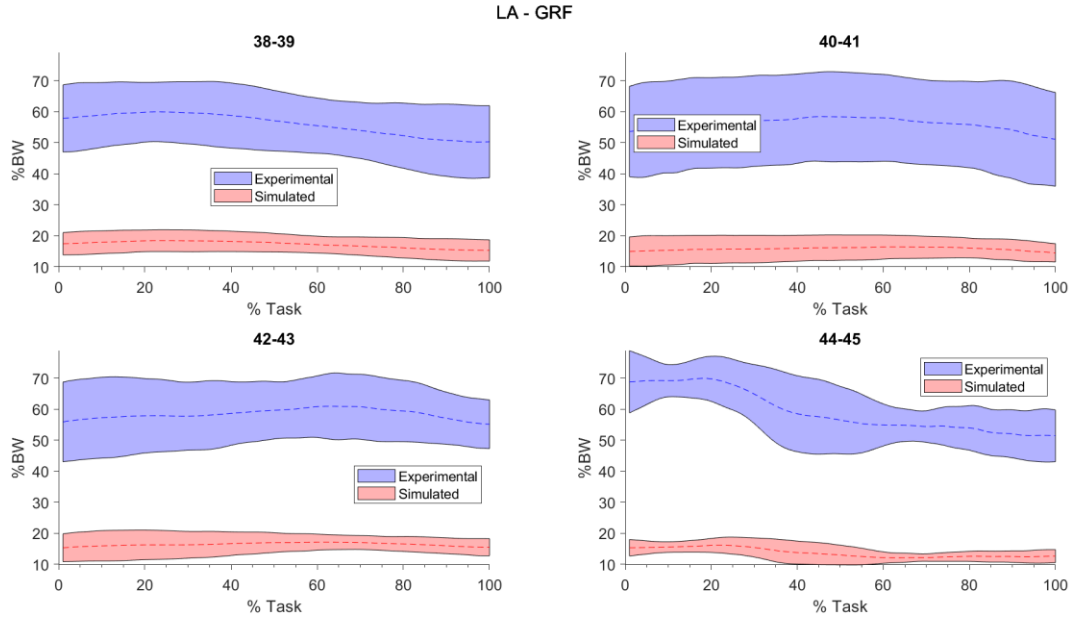

Figure S48: Ground Reaction Force (GRF) for each insole size in percentage of body weight during loaded ascending, expressed in terms of mean and standard deviation. Experimental (Pedar -X® system) in blue and Simulated (prototype layout) in red. Left and right insoles data were averaged.

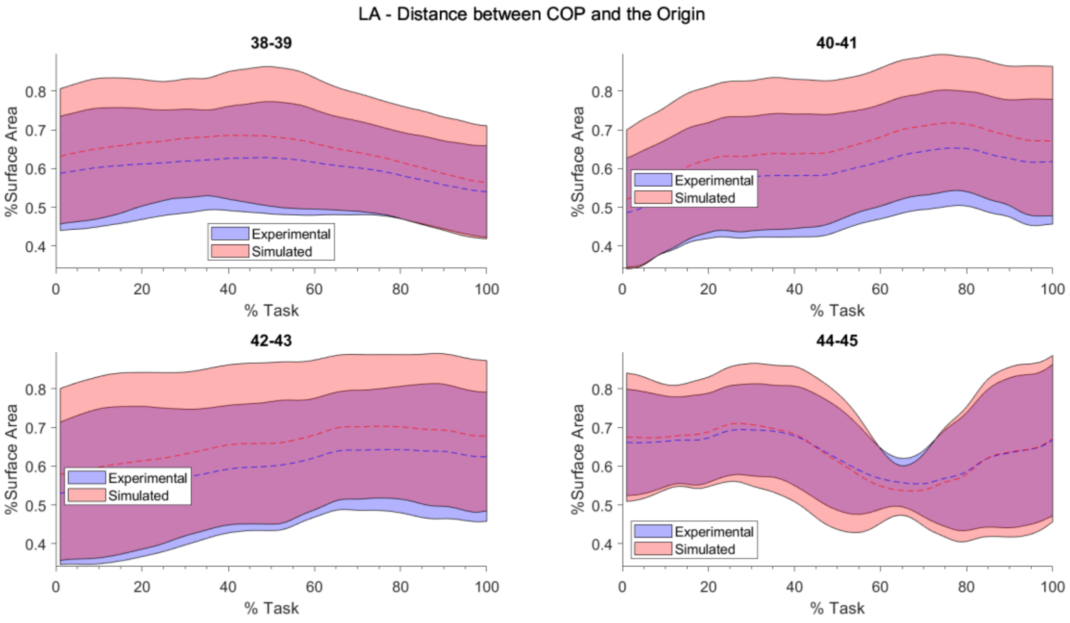

Figure S49: Distance between Center of Pressure (COP) and the Origin for each insole size in percentage of surface area during loaded ascending, expressed in terms of mean and standard deviation. Experimental (Pedar -X® system) in blue and Simulated (prototype layout) in red. Left and right insoles data were averaged.

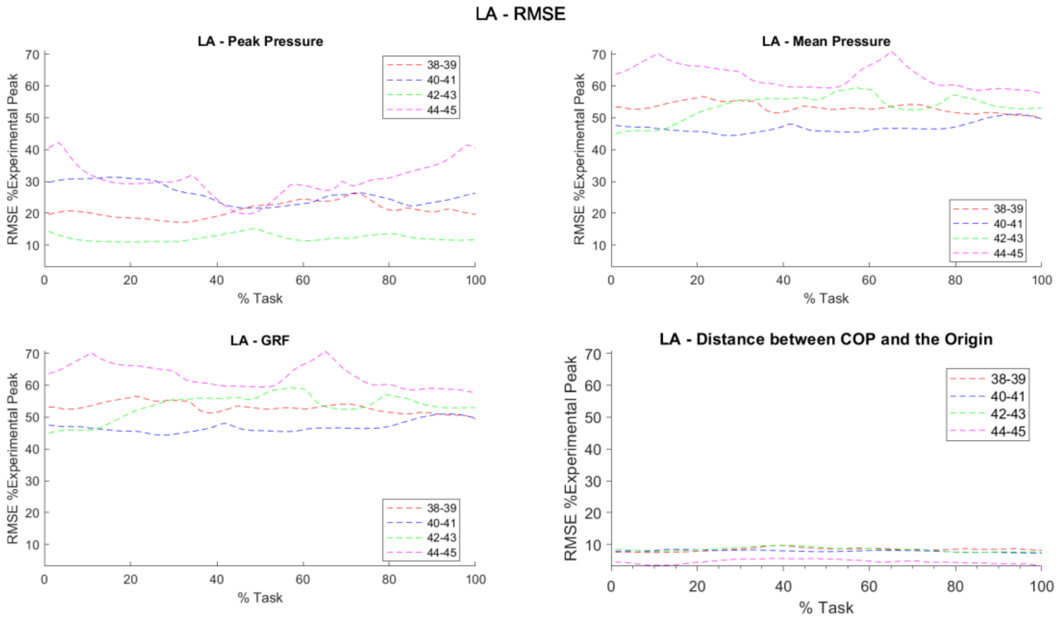

Figure S50: Root Mean Square Error (RMSE) in percentage of Experimental Peak (Pedar -X® system) for each variable and for each insole size during loaded ascending. The insoles were represented respectively: 38-39 in red, 40-41 in blue, 42-43 in green and 44-45 in purple.

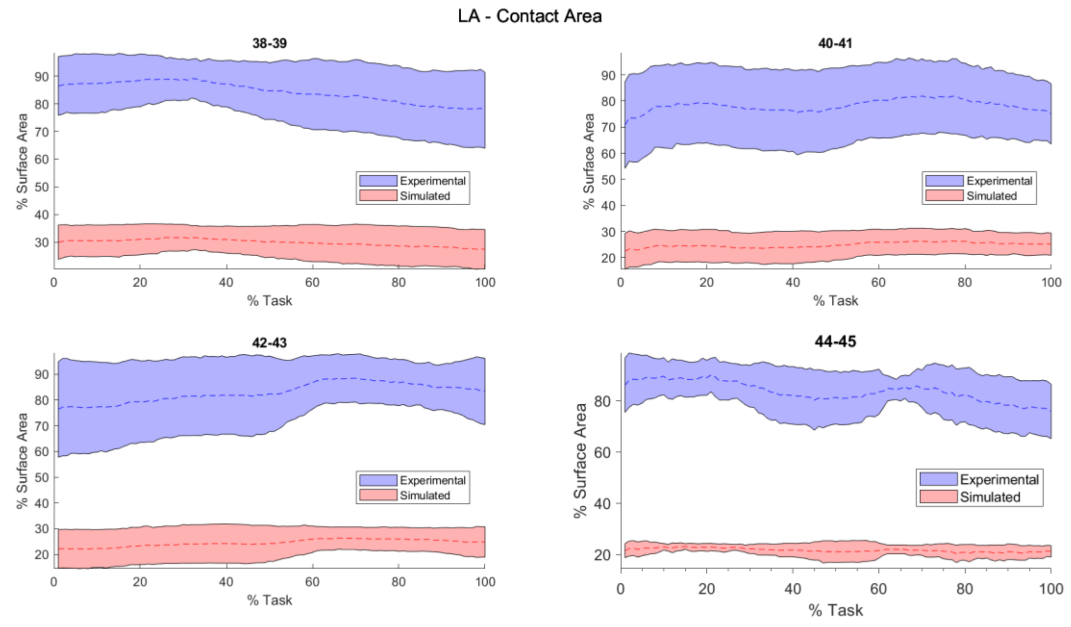

Figure S51: Contact Area for each insole size in percentage of surface area during loaded ascending, expressed in terms of mean and standard deviation. Experimental (Pedar -X® system) in blue and Simulated (prototype layout) in red. Left and right insoles data were averaged.

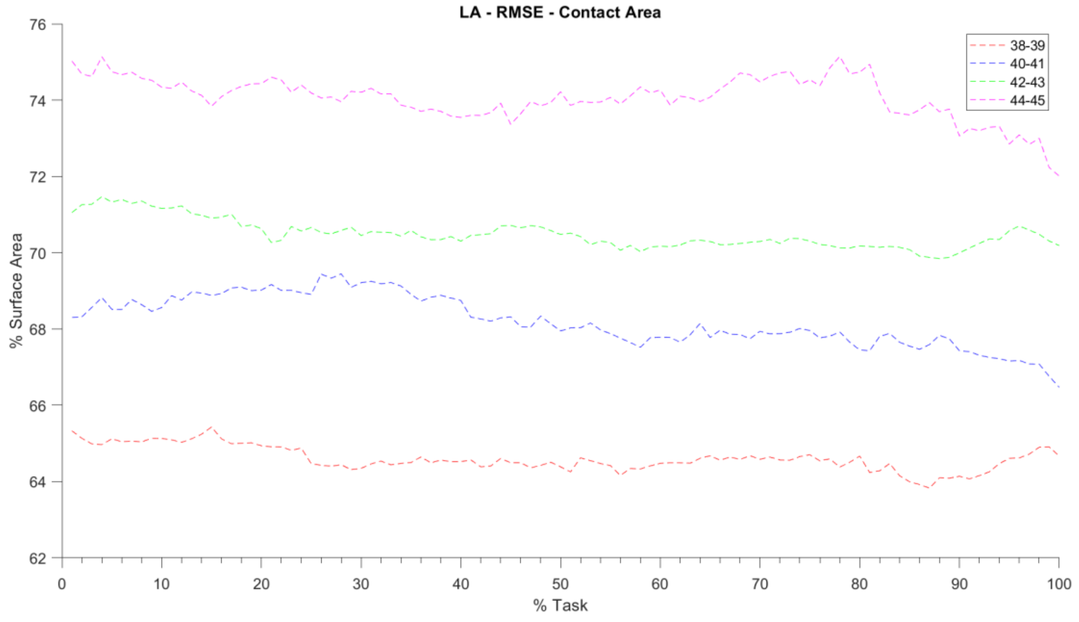

Figure S52: Contact Area Root Mean Square Error (RMSE) in percentage of Surface area for each insole size during loaded ascending. The insoles were represented respectively: 38-39 in red, 40-41 in blue, 42-43 in green and 44-45 in purple.

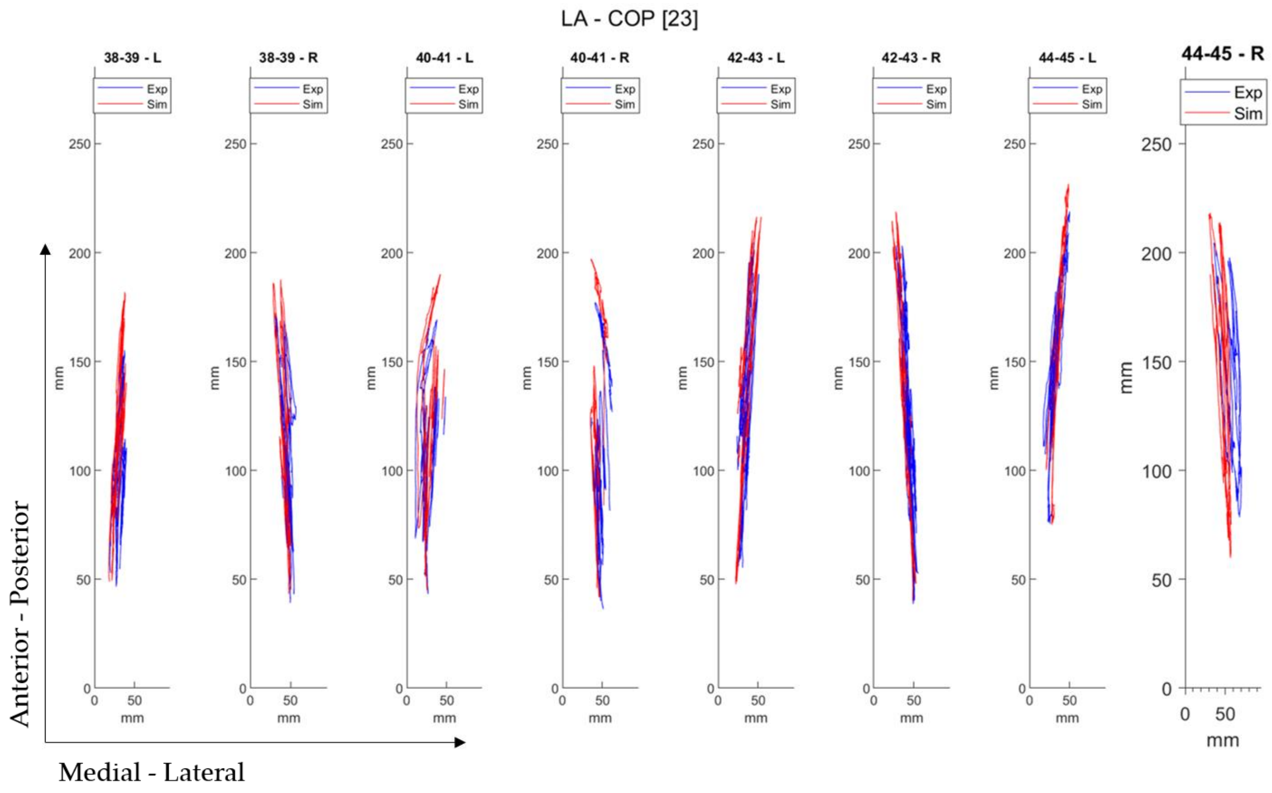

Figure S53: Anterior - Posterior and Medial - Lateral COP, calculated as in [23], Root Mean Square Error (RMSE), in percentage respectively of length insole and of width insole, of each insole size during loaded ascending. The insoles were represented respectively: 38-39 in red, 40-41 in blue, 42-43 in green and 44-45 in purple.

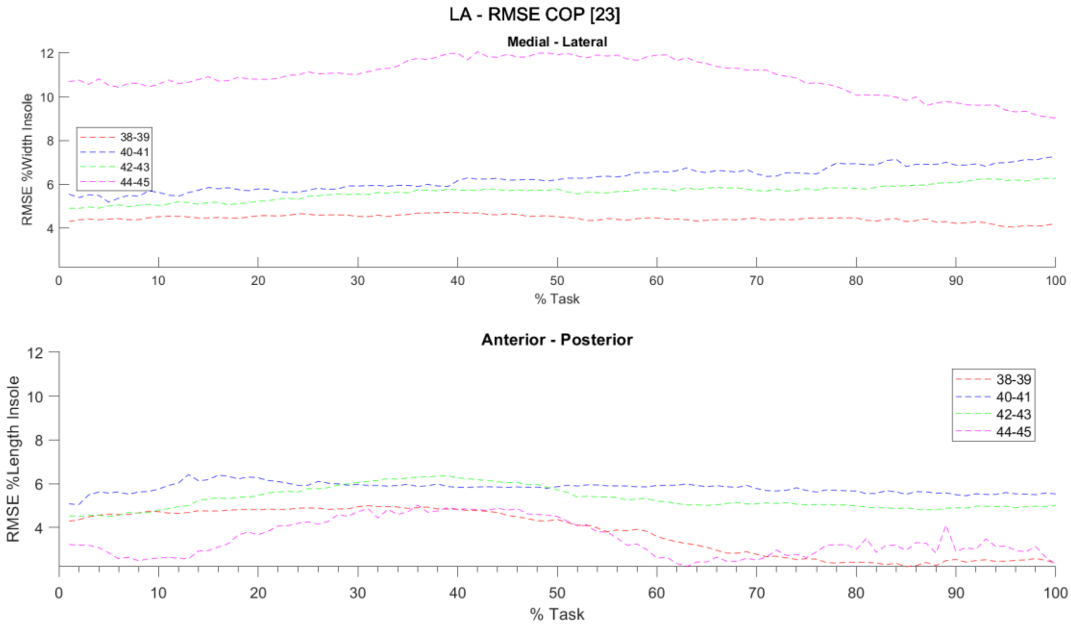

Figure S54: Medial - Lateral and Anterior - Posterior COP, calculated as in [23], Root Mean Square Error (RMSE), in percentage respectively of length insole and of width insole, of each insole size during loaded ascending. The insoles were represented respectively: 38-39 in red, 40-41 in blue, 42-43 in green and 44-45 in purple.

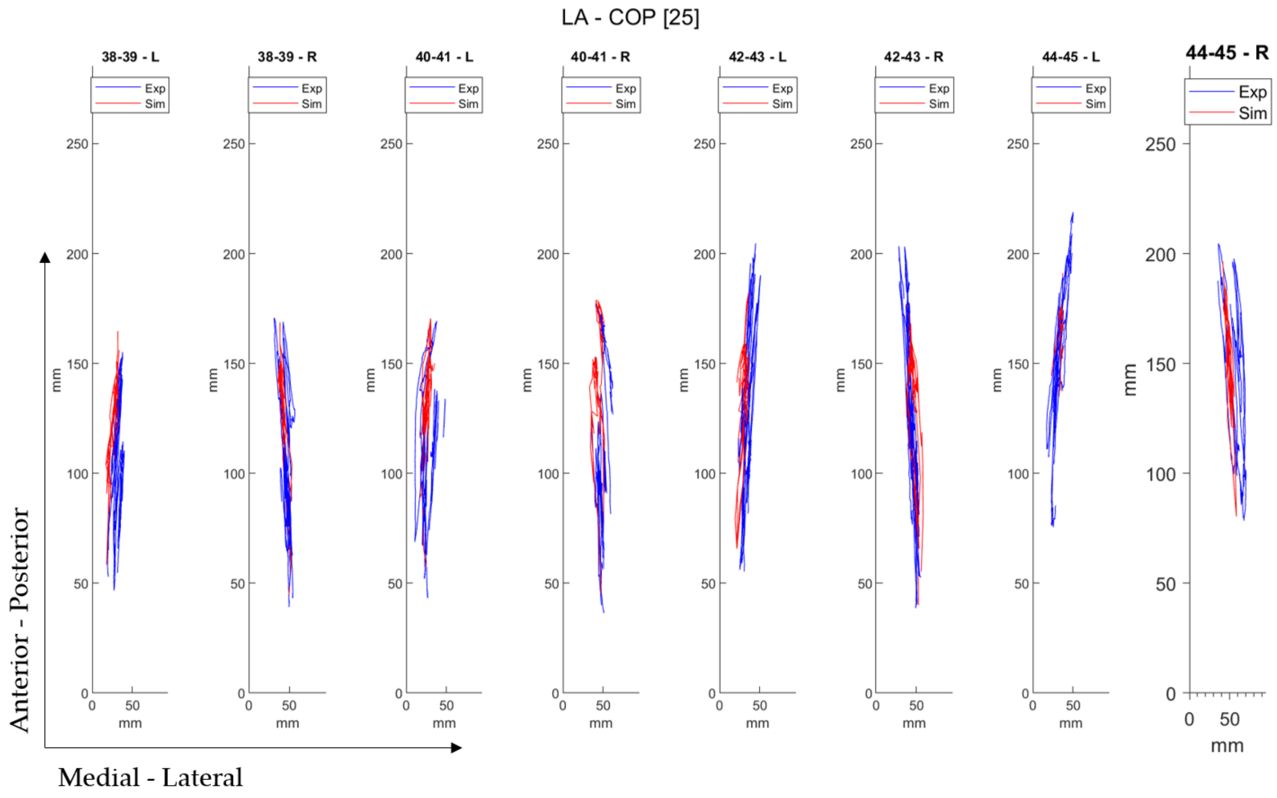

Figure S55: Anterior - Posterior and Medial - Lateral COP, calculated as in [25], Root Mean Square Error (RMSE), in percentage respectively of length insole and of width insole, of each insole size during loaded ascending. The insoles were represented respectively: 38-39 in red, 40-41 in blue, 42-43 in green and 44-45 in purple.

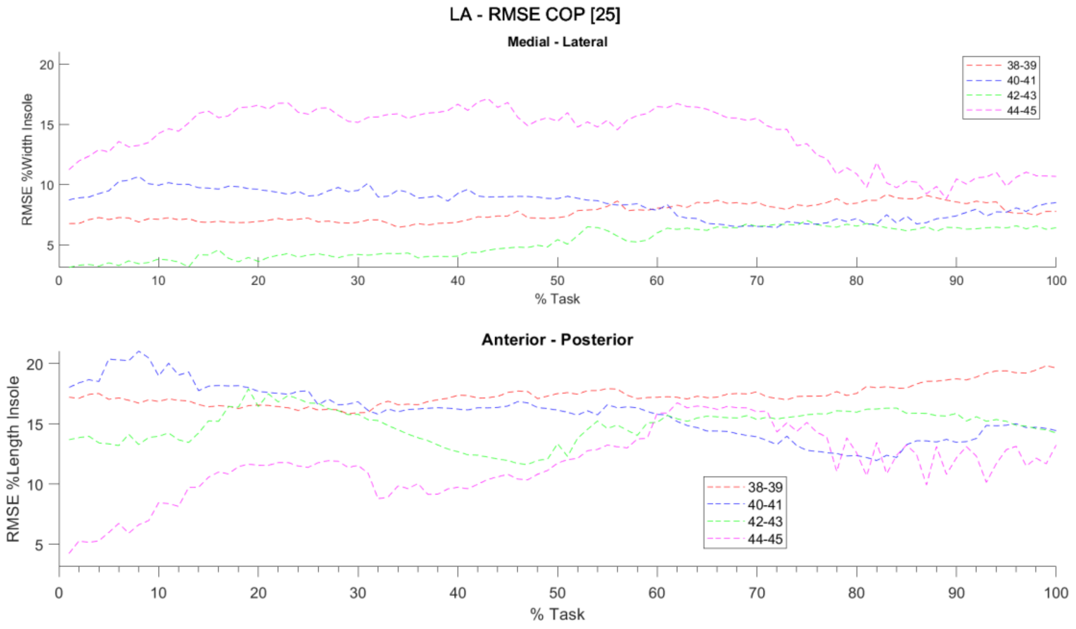

Figure S56: Medial - Lateral and Anterior - Posterior COP, calculated as in [25], Root Mean Square Error (RMSE), in percentage respectively of length insole and of width insole, of each insole size during loaded ascending. The insoles were represented respectively: 38-39 in red, 40-41 in blue, 42-43 in green and 44-45 in purple.

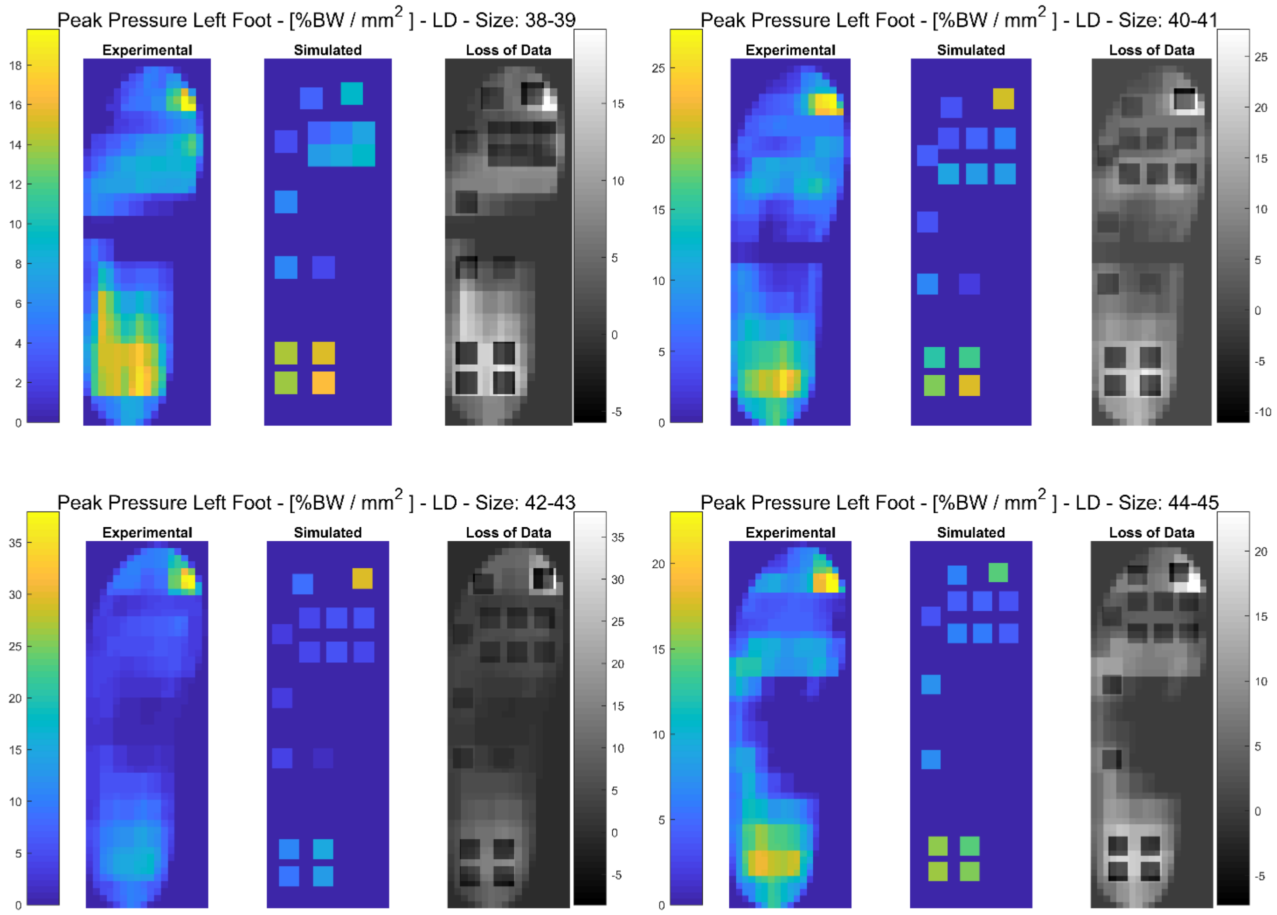

Figure S57: Peak Pressure of left foot for each insole size in %BW/mm^2 during loaded descending. Experimental (Pedar -X® system), simulated (prototype layout) and loss of data (experimental - simulated) footprints were reported. In yellow/white the higher pressure, in blue/black the lower pressure.

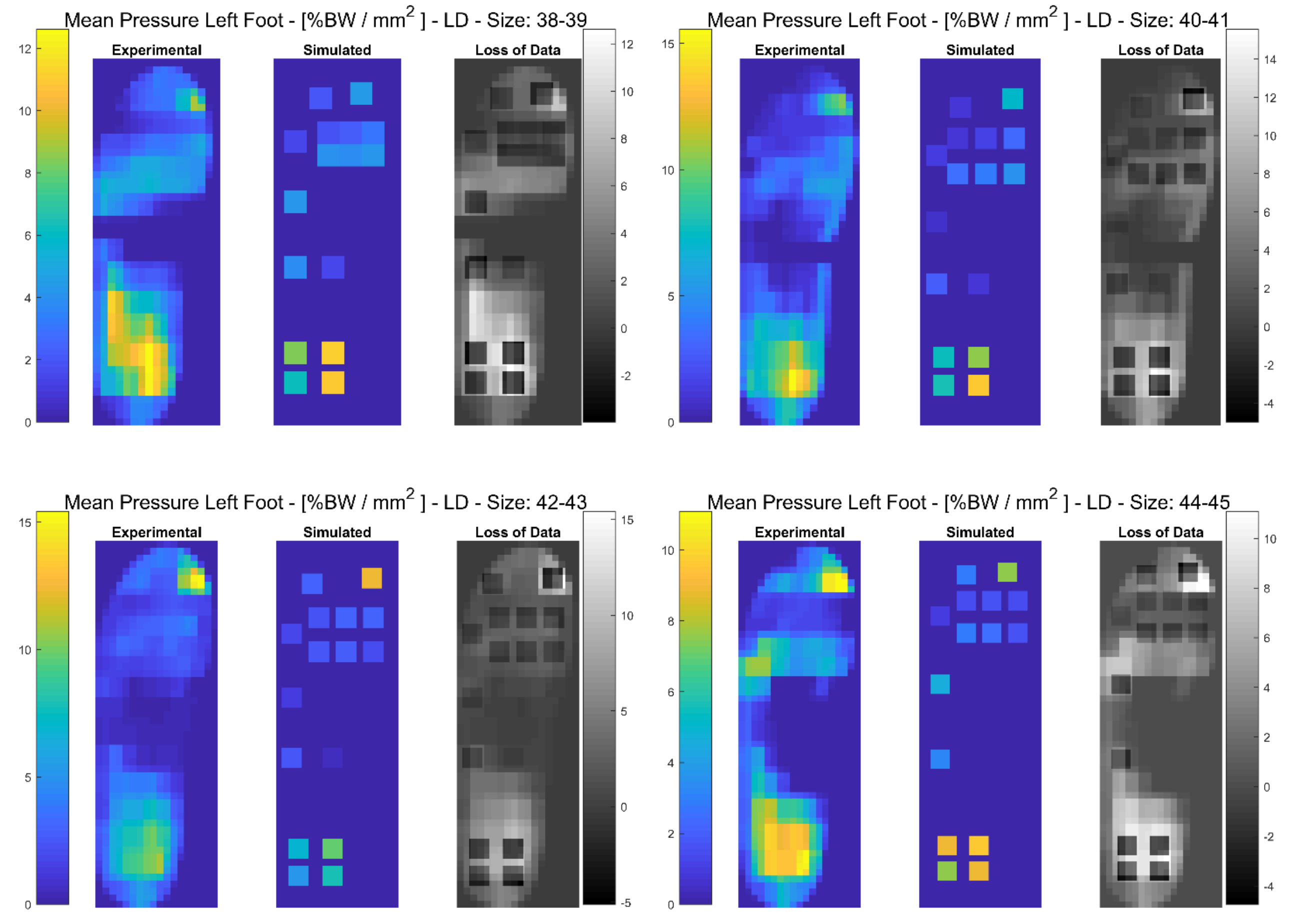

Figure S58: Mean Pressure of left foot for each insole size in %BW/mm^2 during loaded descending. Experimental (Pedar -X® system), simulated (prototype layout) and loss of data (experimental - simulated) footprints were reported. In yellow/white the higher pressure, in blue/black the lower pressure.

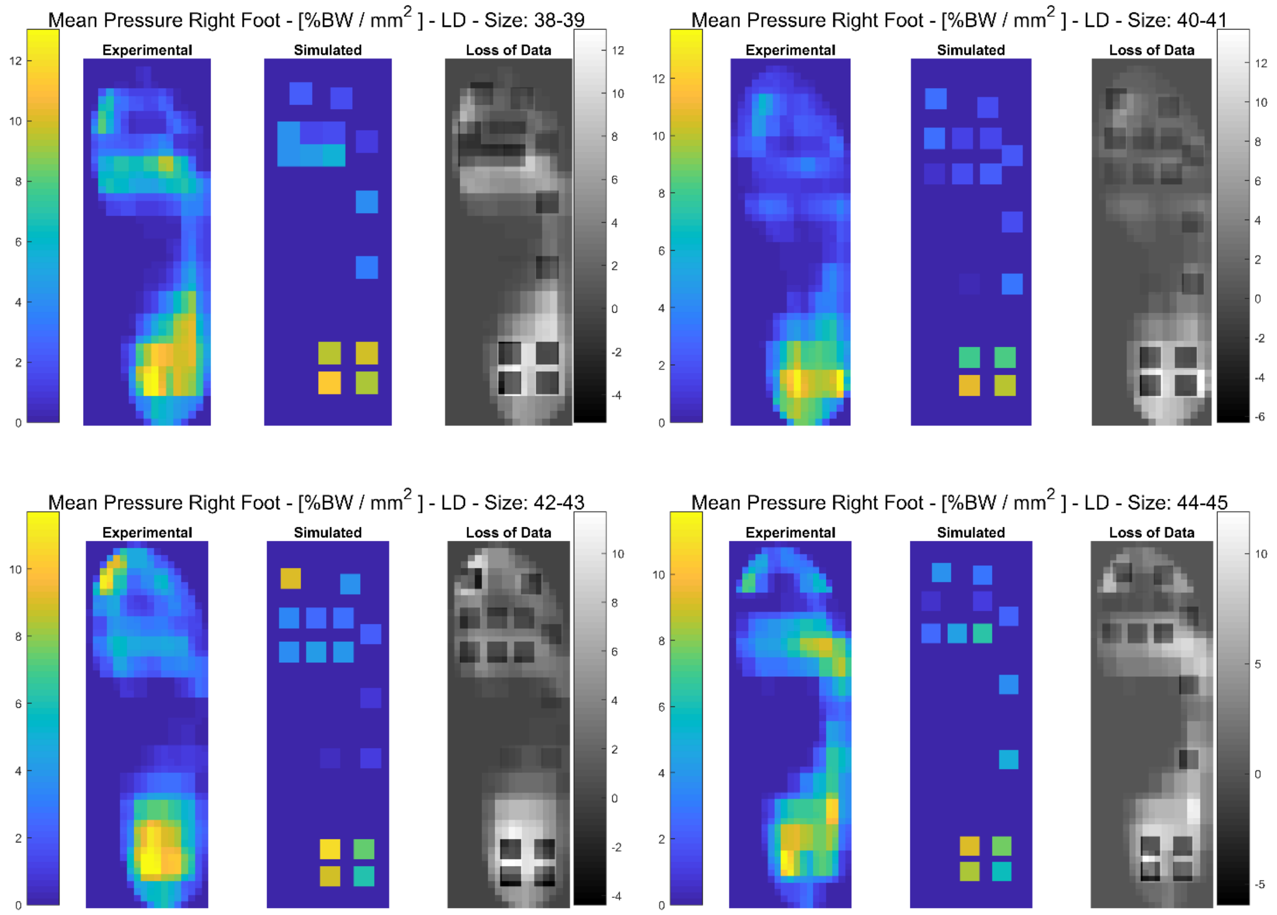

Figure S59: Mean Pressure of right foot for each insole size in %BW/mm² during loaded descending. Experimental (Pedar -X® system), simulated (prototype layout) and loss of data (experimental - simulated) footprints were reported. In yellow/white the higher pressure, in blue/black the lower pressure.

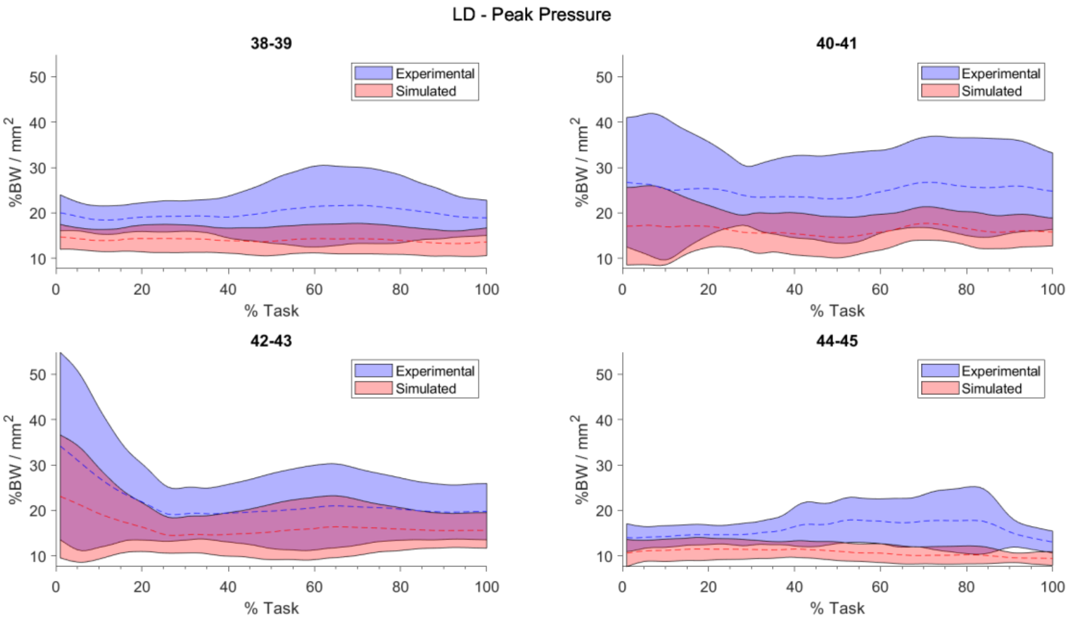

Figure S60: Peak Pressure for each insole size in %BW/mm² during loaded descending, expressed in terms of mean and standard deviation. Experimental (Pedar -X® system) in blue and Simulated (prototype layout) in red. Left and right insoles data were averaged.

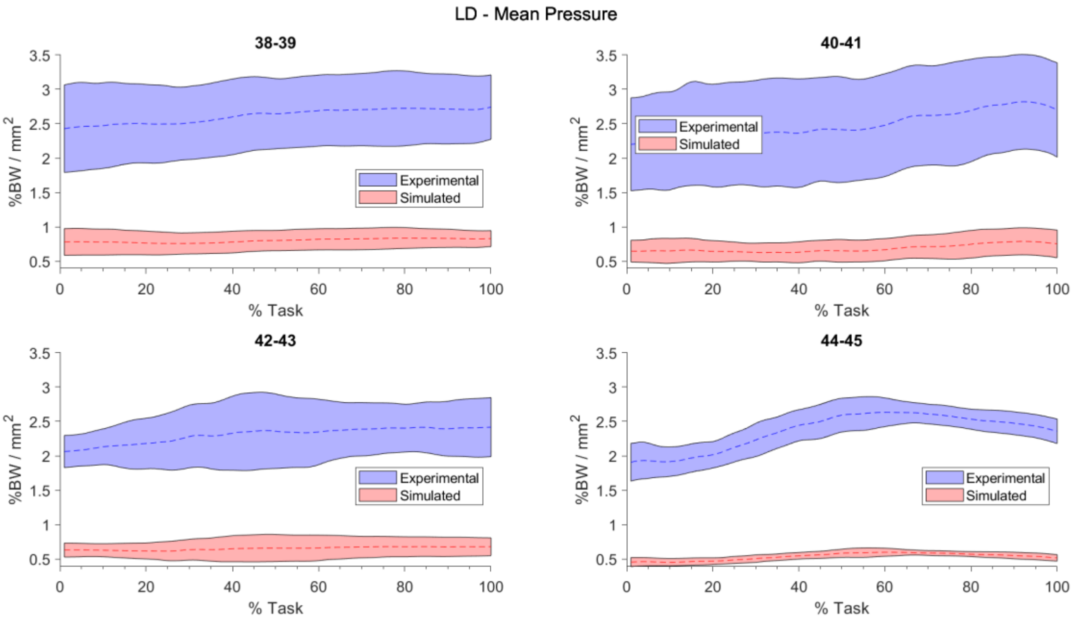

Figure S61: Mean Pressure for each insole size in %BW/mm^2 during loaded descending, expressed in terms of mean and standard deviation. Experimental (Pedar -X® system) in blue and Simulated (prototype layout) in red. Left and right insoles data were averaged.

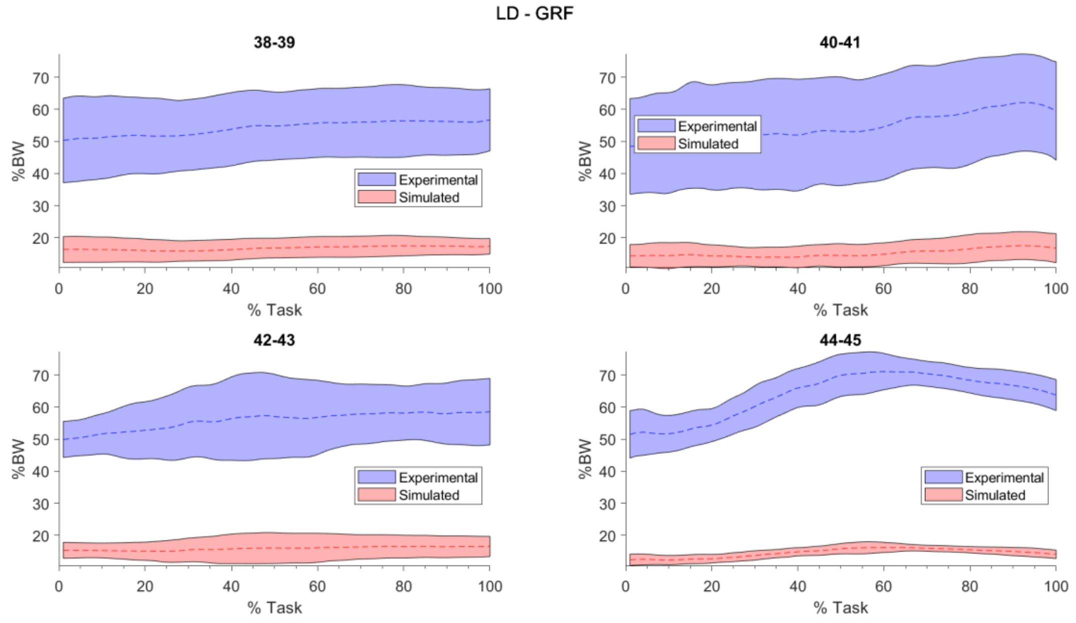

Figure S62: Ground Reaction Force (GRF) for each insole size in percentage of body weight during loaded descending, expressed in terms of mean and standard deviation. Experimental (Pedar -X® system) in blue and Simulated (prototype layout) in red. Left and right insoles data were averaged.

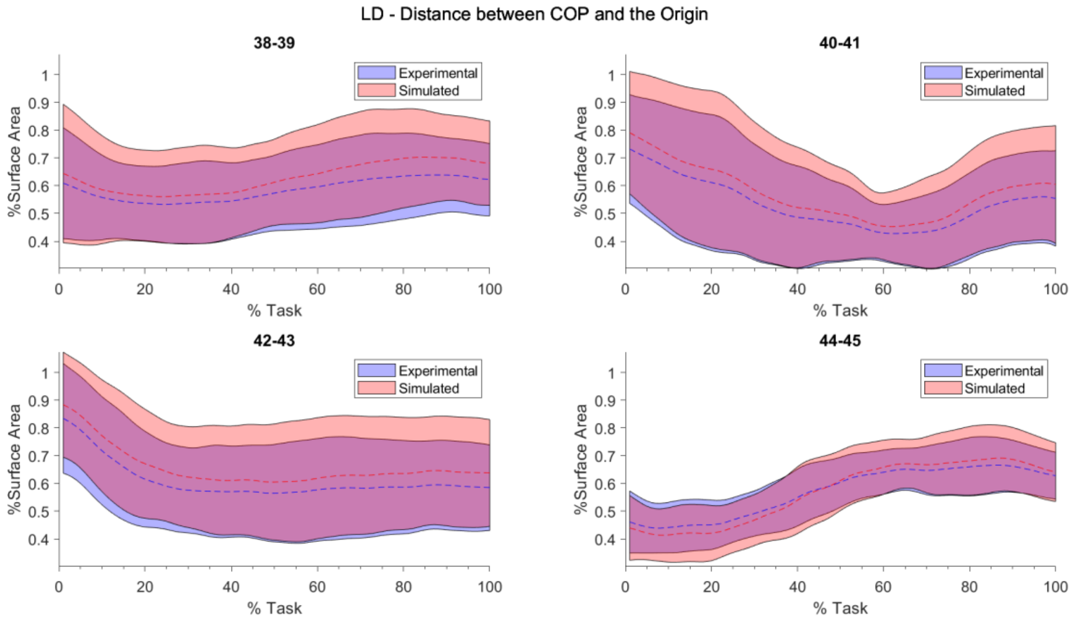

Figure S63: Distance between Center of Pressure (COP) and the Origin for each insole size in percentage of surface area during loaded descending, expressed in terms of mean and standard deviation. Experimental (Pedar -X® system) in blue and Simulated (prototype layout) in red. Left and right insoles data were averaged.

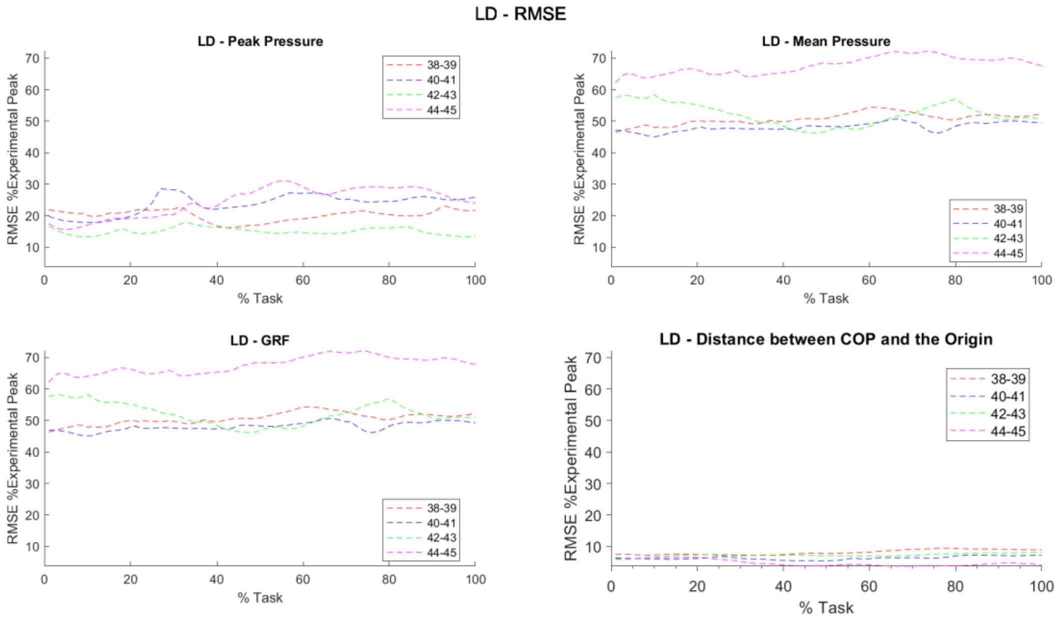

Figure S64: Root Mean Square Error (RMSE) in percentage of Experimental Peak (Pedar -X® system) for each variable and for each insole size during loaded descending. The insoles size were represented respectively: 38-39 in red, 40-41 in blue, 42-43 in green and 44-45 in purple.

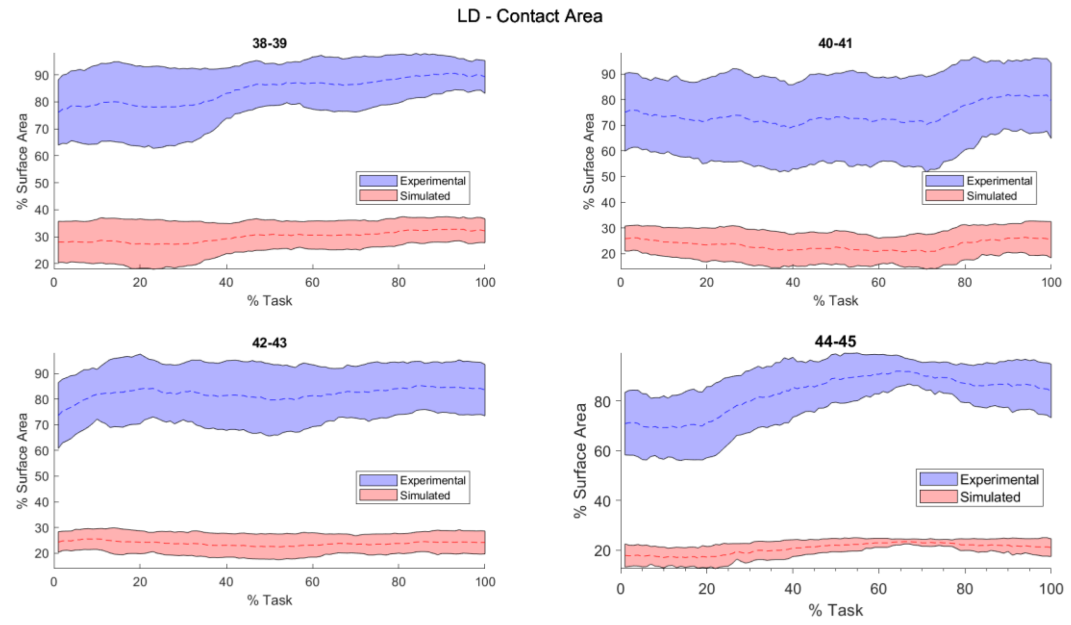

Figure S65: Contact Area for each insole size in percentage of surface area during loaded descending, expressed in terms of mean and standard deviation. Experimental (Pedar -X® system) in blue and Simulated (prototype layout) in red. Left and right insoles data were averaged.

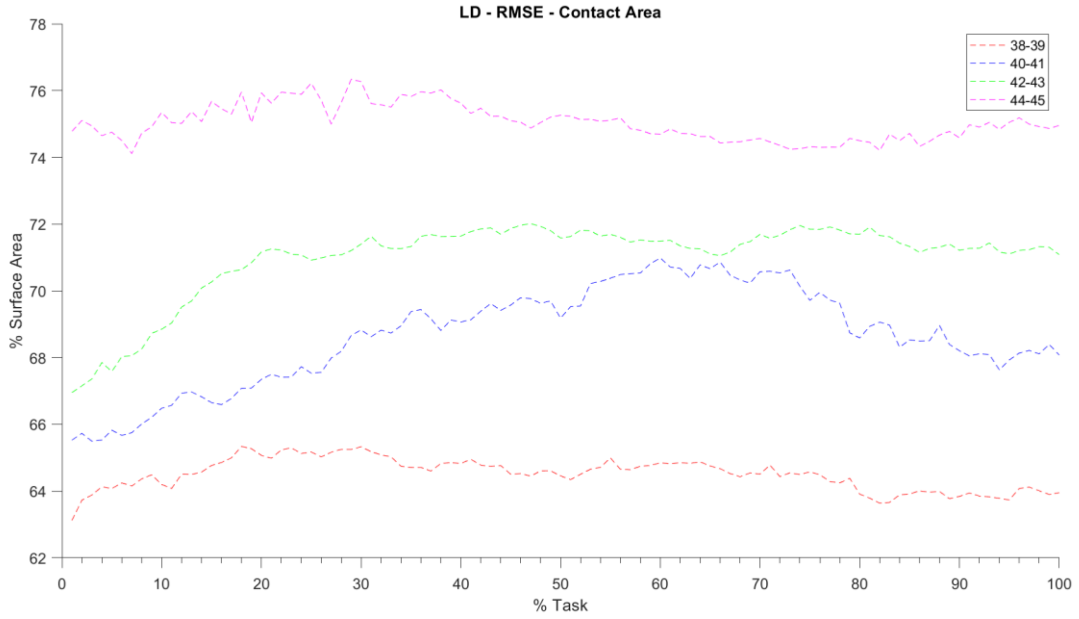

Figure S66: Contact Area Root Mean Square Error (RMSE) in percentage of Surface area for each insole size during loaded descending. The insoles were represented respectively: 38-39 in red, 40-41 in blue, 42-43 in green and 44-45 in purple.

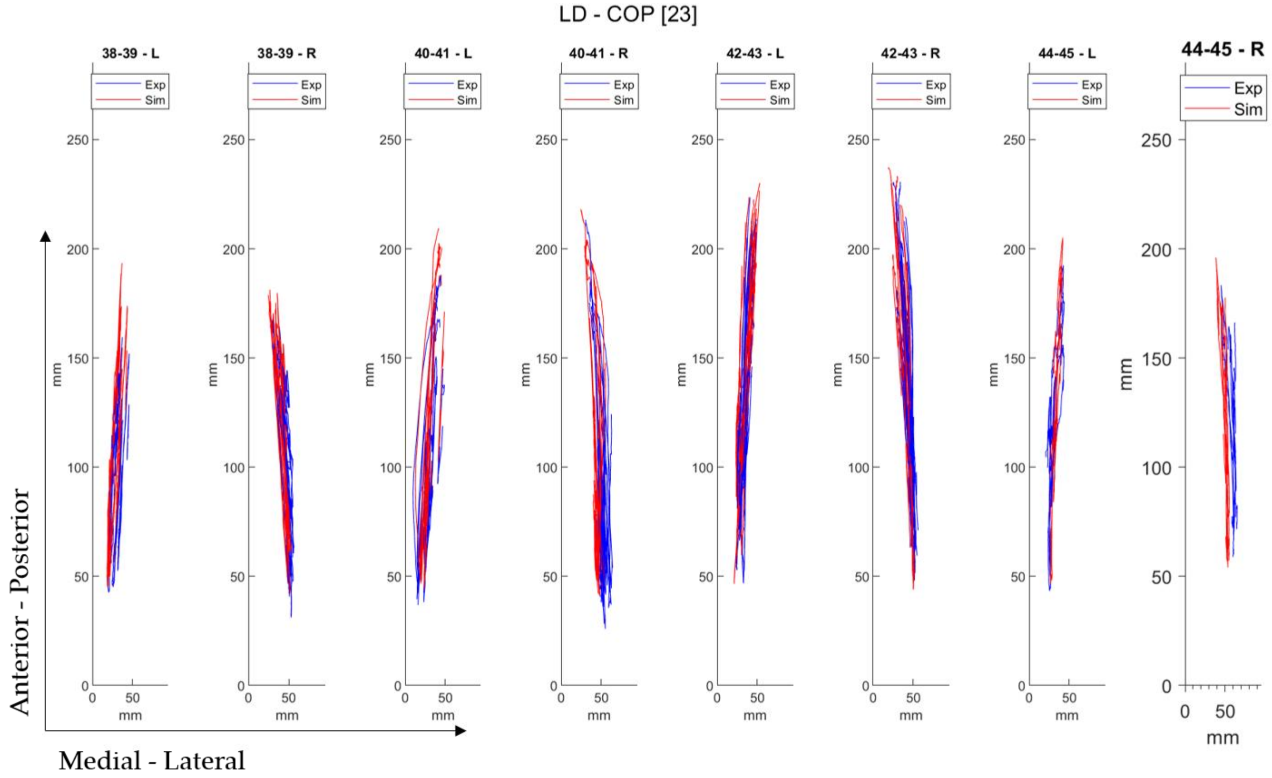

Figure S67: Anterior - Posterior and Medial - Lateral COP, calculated as in [23], Root Mean Square Error (RMSE), in percentage respectively of length insole and of width insole, of each insole size during loaded descending. The insoles were represented respectively: 38-39 in red, 40-41 in blue, 42-43 in green and 44-45 in purple.

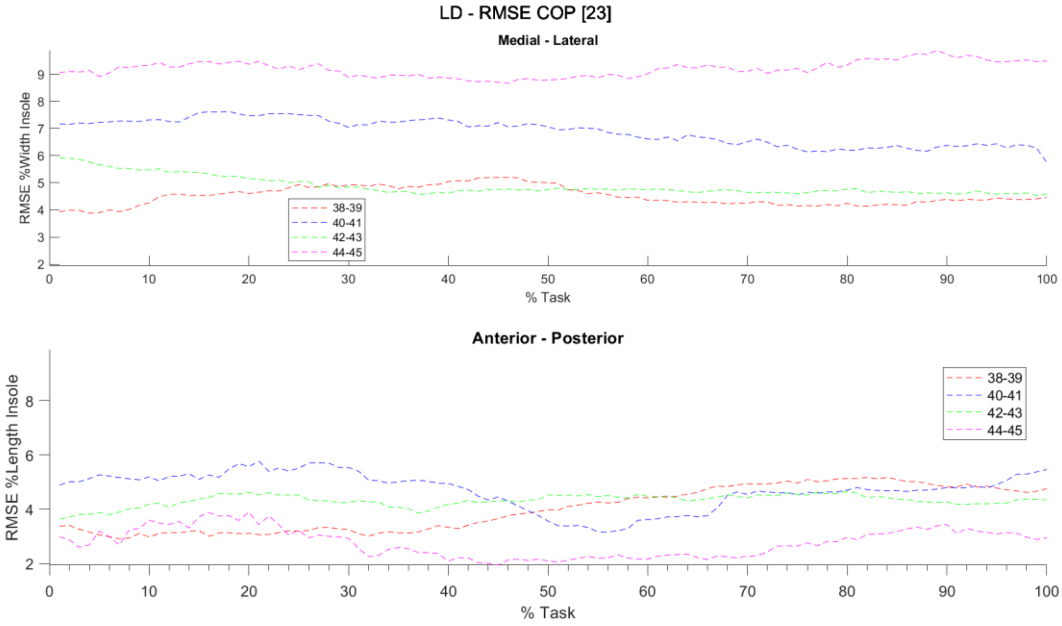

Figure S68: Medial - Lateral and Anterior - Posterior COP, calculated as in [23], Root Mean Square Error (RMSE), in percentage respectively of length insole and of width insole, of each insole size during loaded descending. The insoles were represented respectively: 38-39 in red, 40-41 in blue, 42-43 in green and 44-45 in purple.

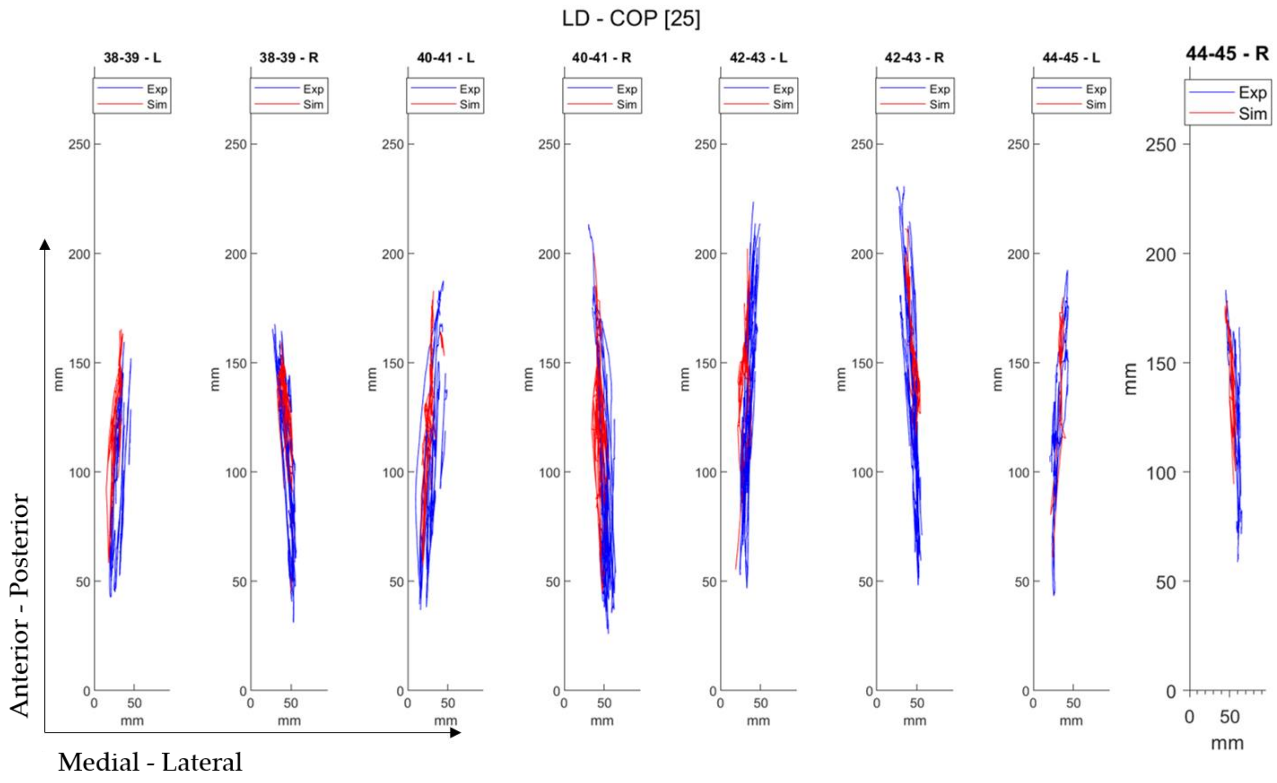

Figure S69: Anterior - Posterior and Medial - Lateral COP, calculated as in [25], Root Mean Square Error (RMSE), in percentage respectively of length insole and of width insole, of each insole size during loaded descending. The insoles were represented respectively: 38-39 in red, 40-41 in blue, 42-43 in green and 44-45 in purple.

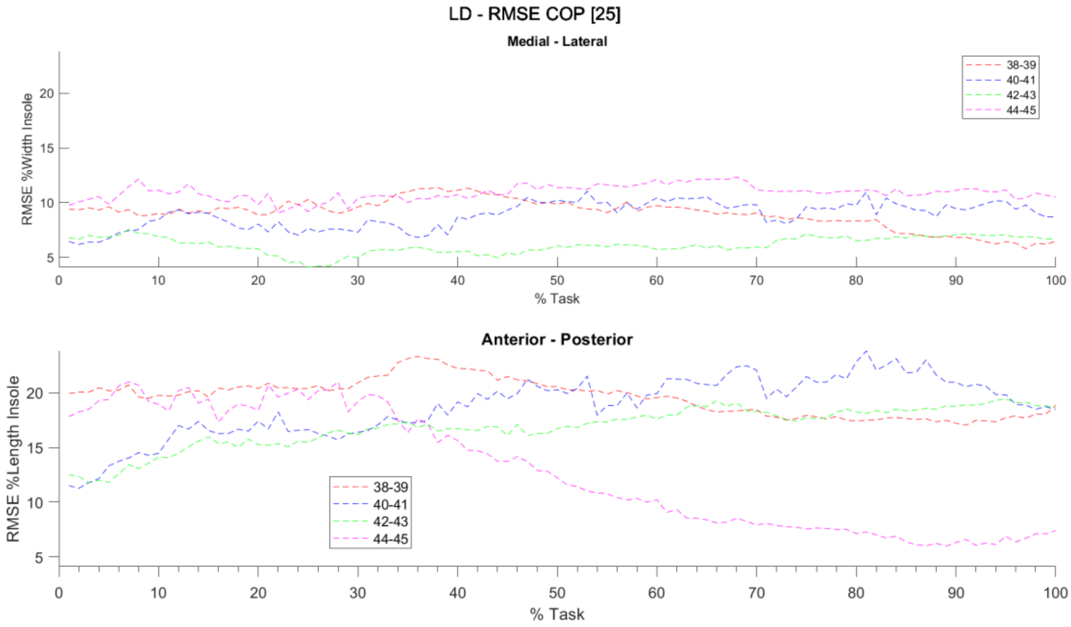

Figure S70: Medial - Lateral and Anterior - Posterior COP, calculated as in [25], Root Mean Square Error (RMSE), in percentage respectively of length insole and of width insole, of each insole size during loaded descending. The insoles were represented respectively: 38-39 in red, 40-41 in blue, 42-43 in green and 44-45 in purple.

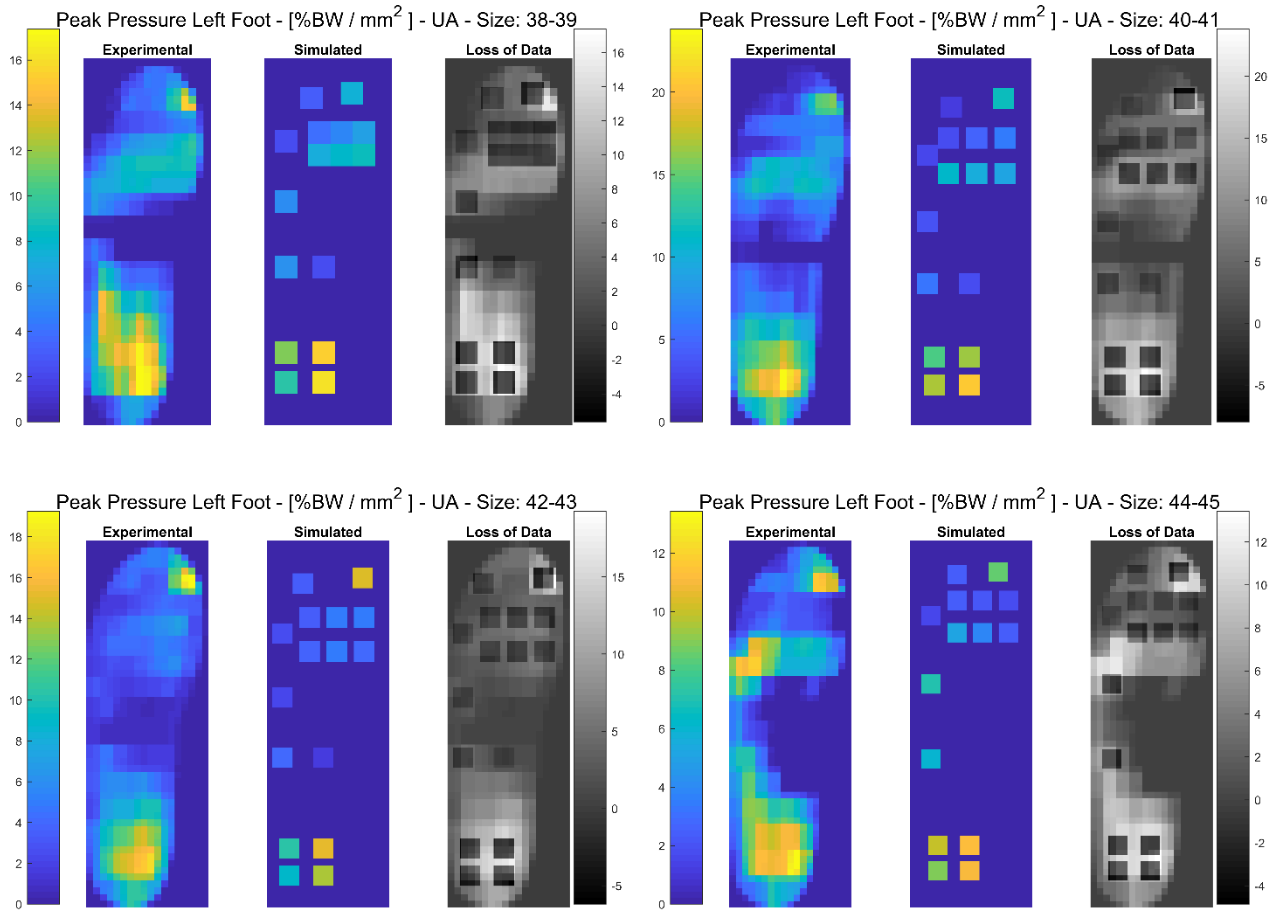

Figure S71: Peak Pressure of left foot for each insole size in %BW/mm² during unloaded ascending. Experimental (Pedar -X® system), simulated (prototype layout) and loss of data (experimental - simulated) footprints were reported. In yellow/white the higher pressure, in blue/black the lower pressure.

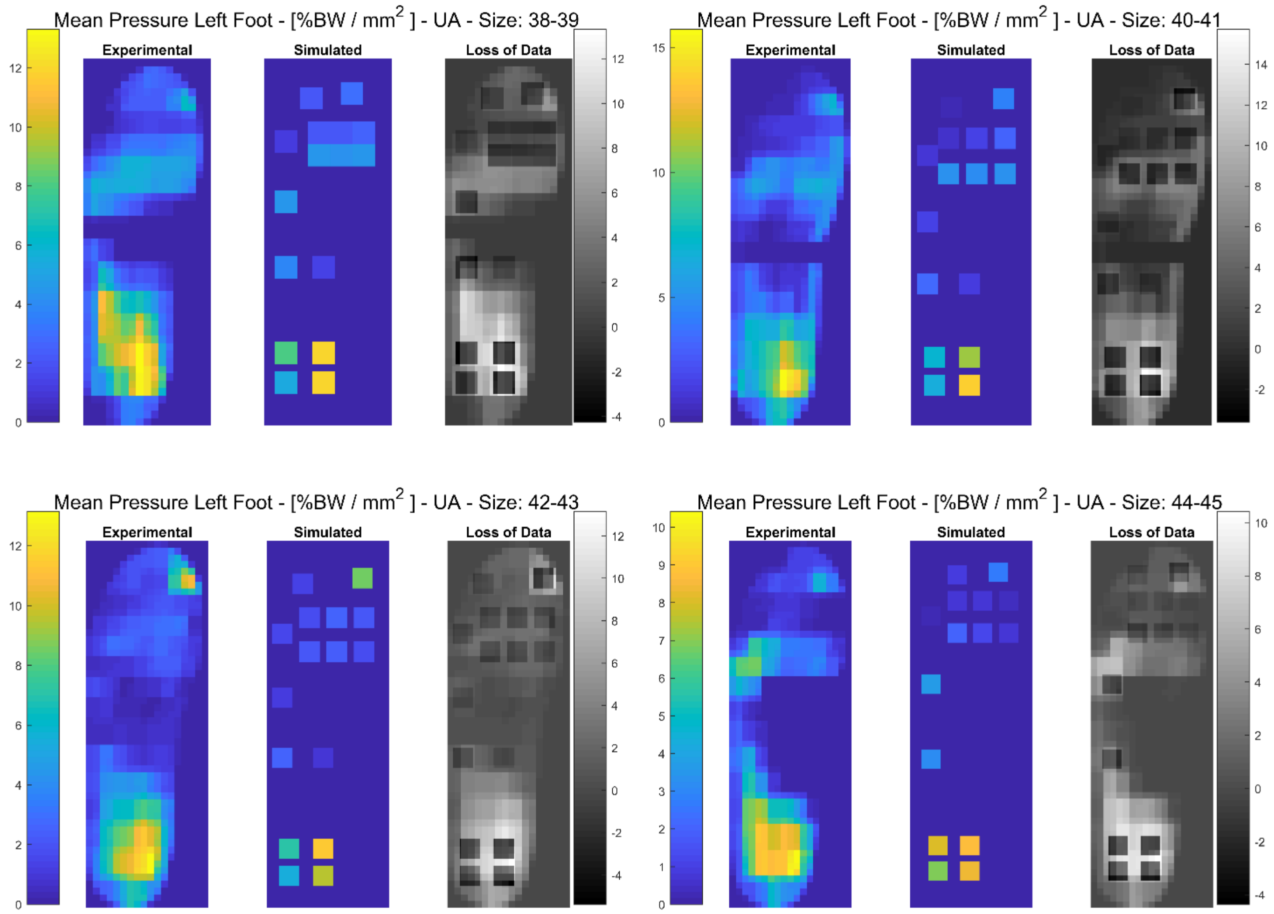

Figure S72: Mean Pressure of left foot for each insole size in %BW/mm² during unloaded ascending. Experimental (Pedar -X® system), simulated (prototype layout) and loss of data (experimental - simulated) footprints were reported. In yellow/white the higher pressure, in blue/black the lower pressure.

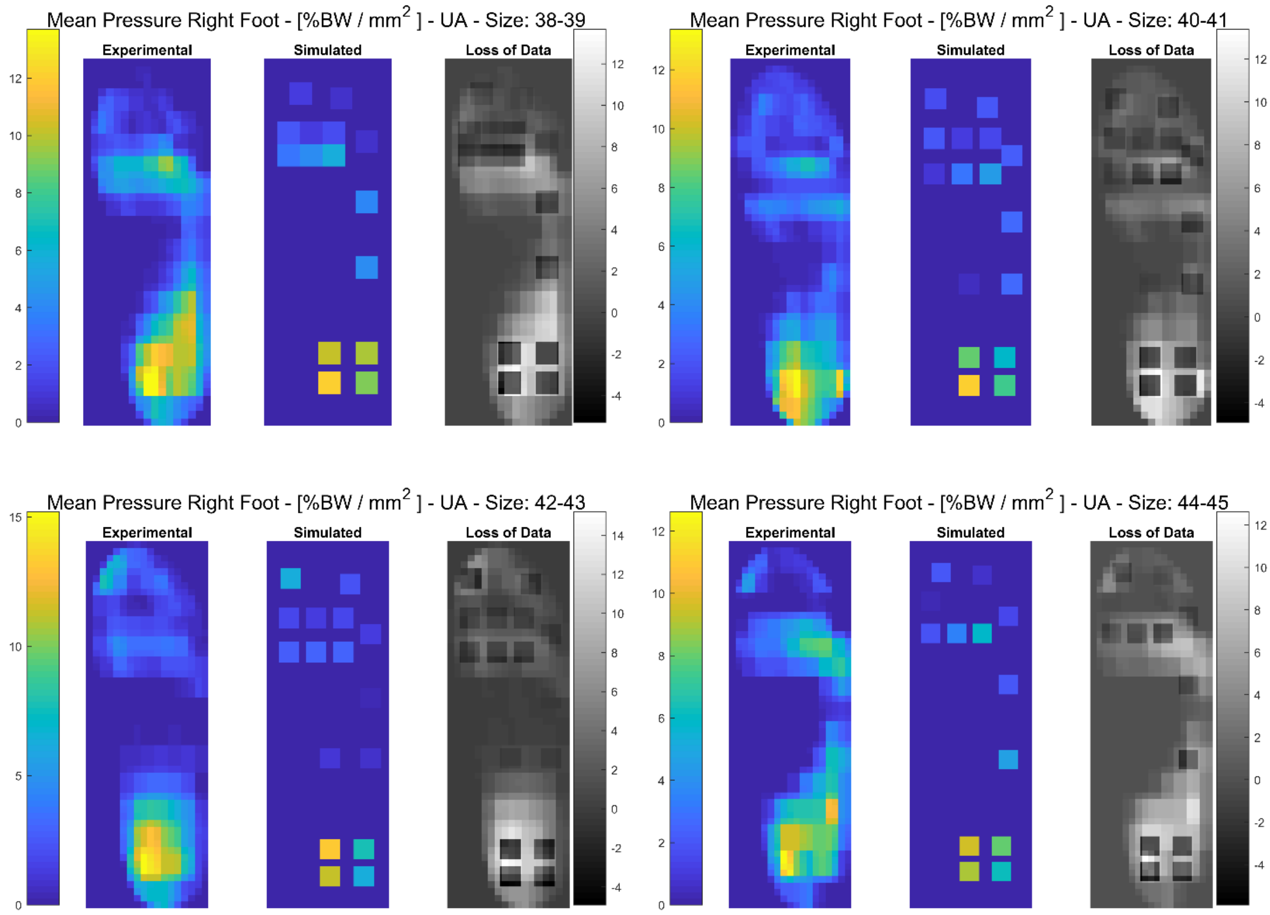

Figure S73: Mean Pressure of right foot for each insole size in %BW/mm² during unloaded ascending. Experimental (Pedar -X® system), simulated (prototype layout) and loss of data (experimental - simulated) footprints were reported. In yellow/white the higher pressure, in blue/black the lower pressure.

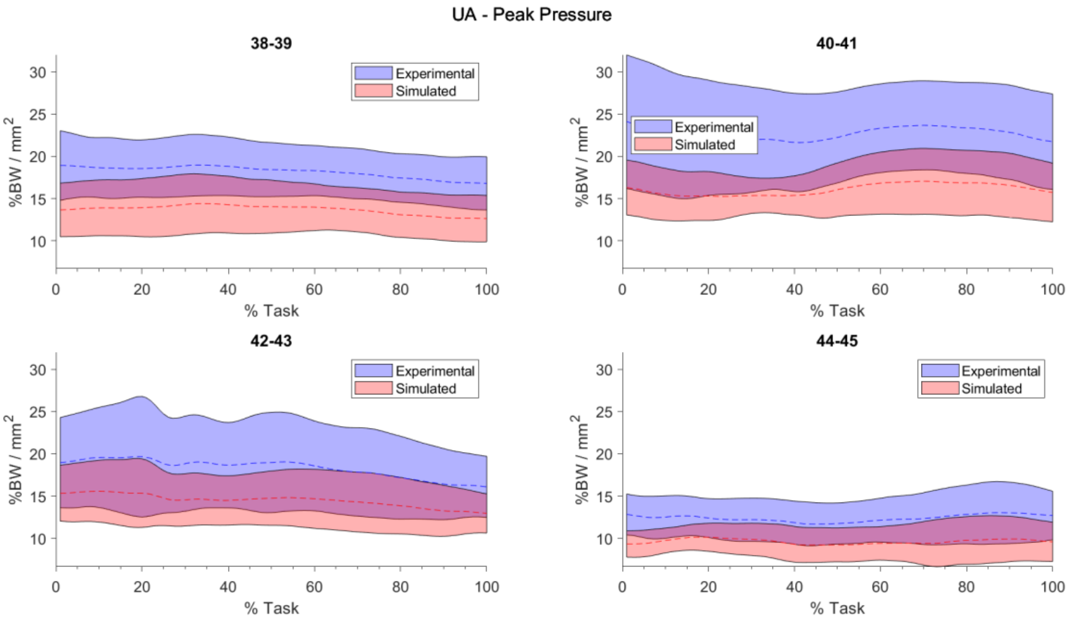

Figure S74: Peak Pressure for each insole size in %BW/mm² during unloaded ascending, expressed in terms of mean and standard deviation. Experimental (Pedar -X® system) in blue and Simulated (prototype layout) in red. Left and right insoles data were averaged.

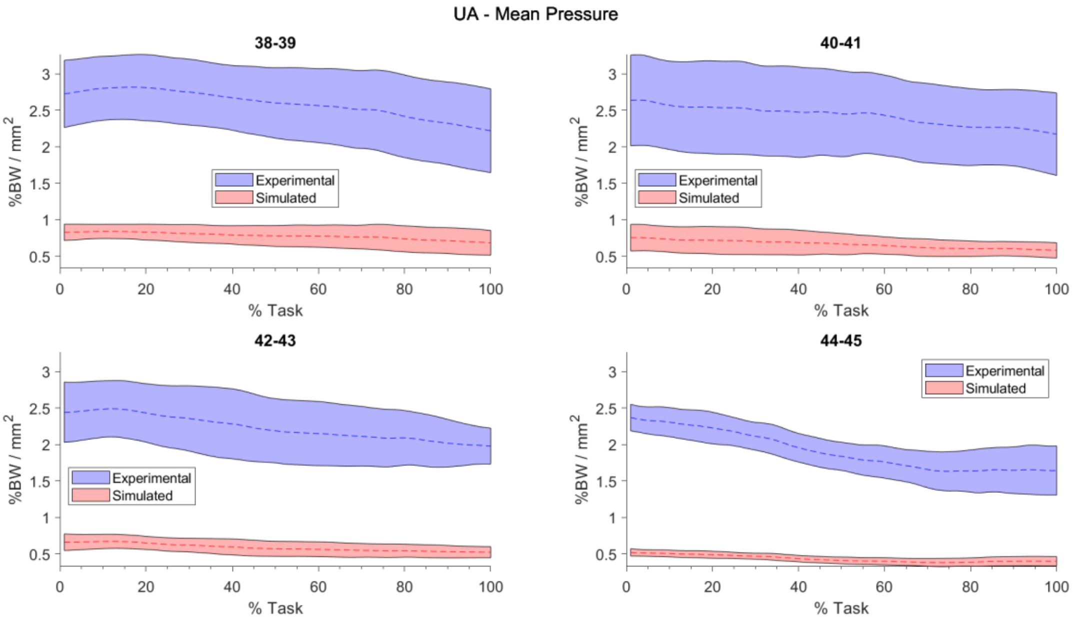

Figure S75: Mean Pressure for each insole size in %BW/mm² during unloaded ascending, expressed in terms of mean and standard deviation. Experimental (Pedar -X® system) in blue and Simulated (prototype layout) in red. Left and right insoles data were averaged.

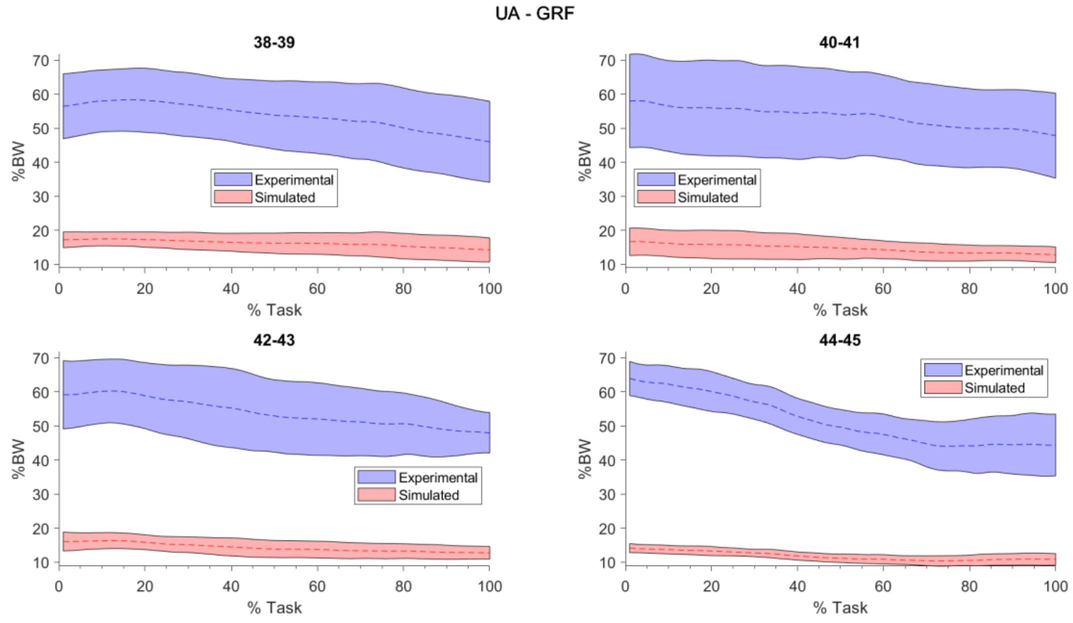

Figure S76: Ground Reaction Force (GRF) for each insole size in percentage of body weight during unloaded ascending, expressed in terms of mean and standard deviation. Experimental (Pedar -X® system) in blue and Simulated (prototype layout) in red. Left and right insoles data were averaged.

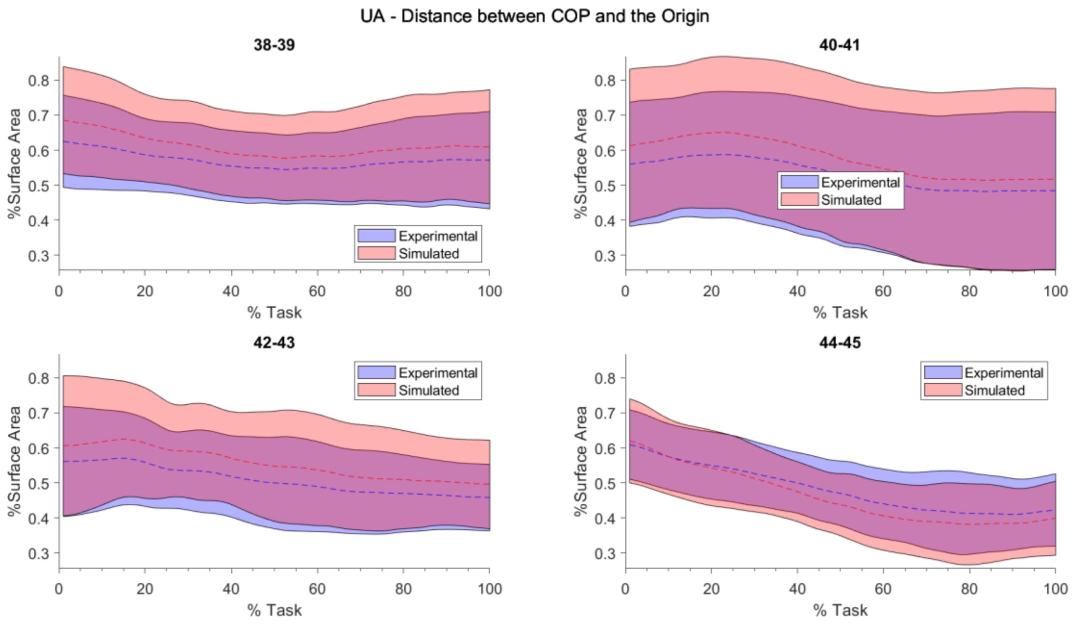

Figure S77: Distance between Center of Pressure (COP) and the Origin for each insole size in percentage of surface area during unloaded ascending, expressed in terms of mean and standard deviation. Experimental (Pedar -X® system) in blue and Simulated (prototype layout) in red. Left and right insoles data were averaged.

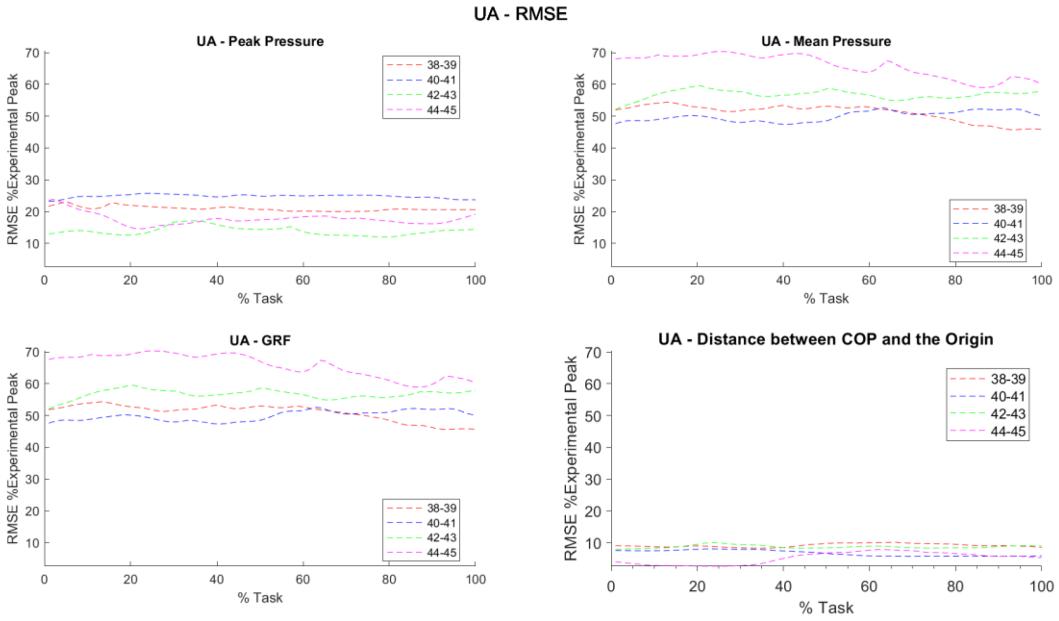

Figure S78: Root Mean Square Error (RMSE) in percentage of Experimental Peak (Pedar -X® system) for each variable and for each insole size during unloaded ascending. The insoles were represented respectively: 38-39 in red, 40-41 in blue, 42-43 in green and 44-45 in purple.

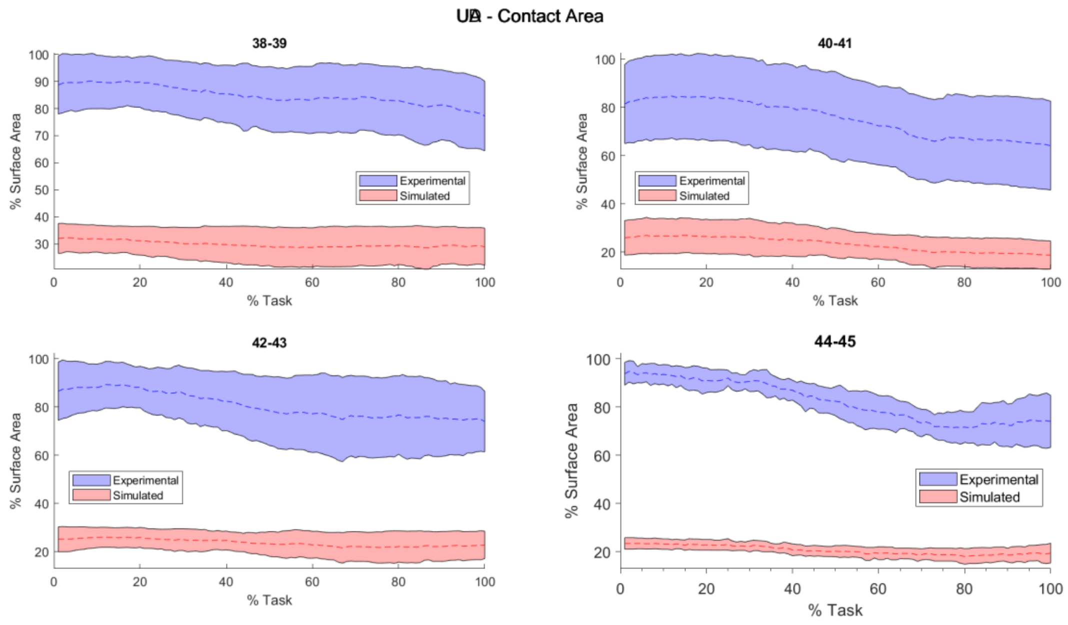

Figure S79: Contact Area for each insole size in percentage of surface area during unloaded ascending, expressed in terms of mean and standard deviation. Experimental (Pedar -X® system) in blue and Simulated (prototype layout) in red. Left and right insoles data were averaged.

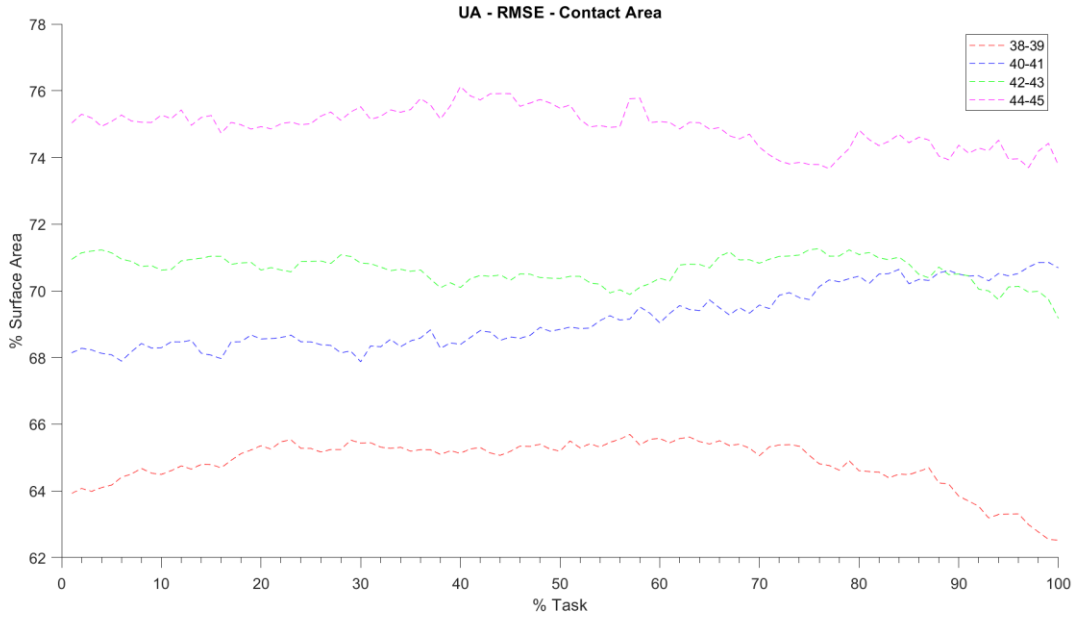

Figure S80: Contact Area Root Mean Square Error (RMSE) in percentage of Surface area for each insole size during unloaded ascending. The insoles were represented respectively: 38-39 in red, 40-41 in blue, 42-43 in green and 44-45 in purple.

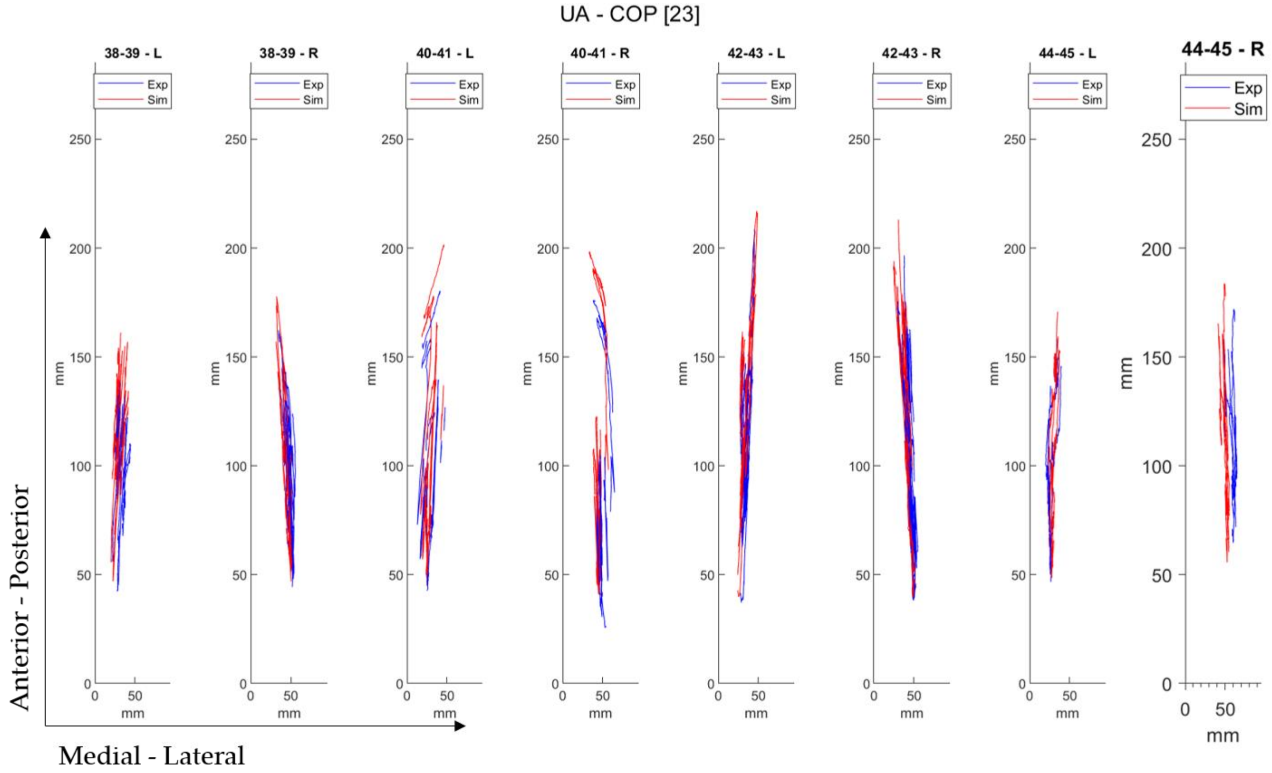

Figure S81: Anterior - Posterior and Medial - Lateral COP, calculated as in [23], Root Mean Square Error (RMSE), in percentage respectively of length insole and of width insole, of each insole size during unloaded ascending. The insoles were represented respectively: 38-39 in red, 40-41 in blue, 42-43 in green and 44-45 in purple.

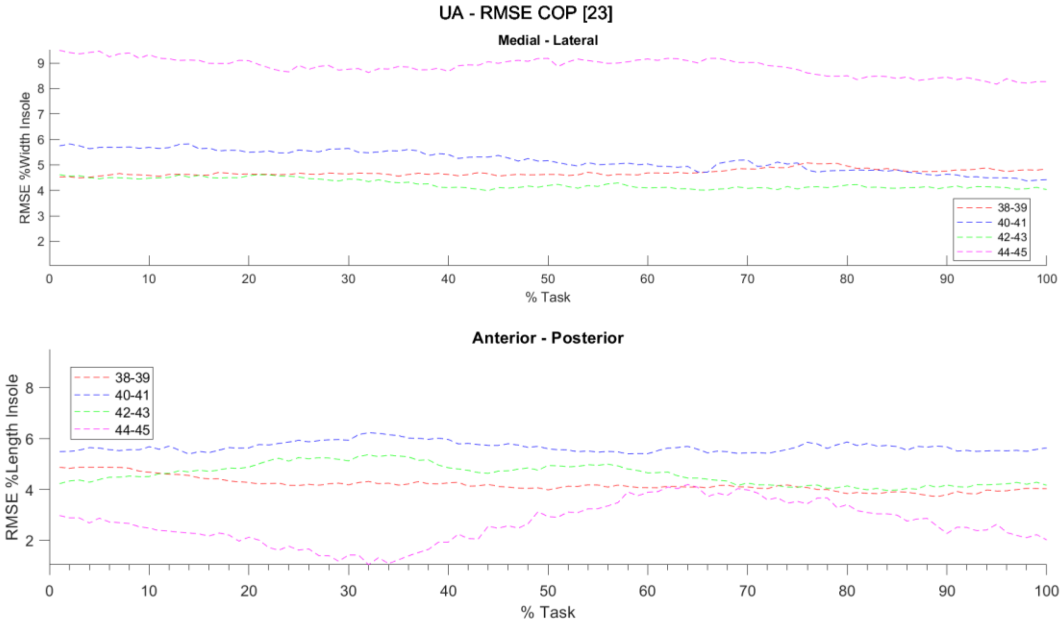

Figure S82: Medial - Lateral and Anterior - Posterior COP, calculated as in [23], Root Mean Square Error (RMSE), in percentage respectively of length insole and of width insole, of each insole size during unloaded ascending. The insoles were represented respectively: 38-39 in red, 40-41 in blue, 42-43 in green and 44-45 in purple.

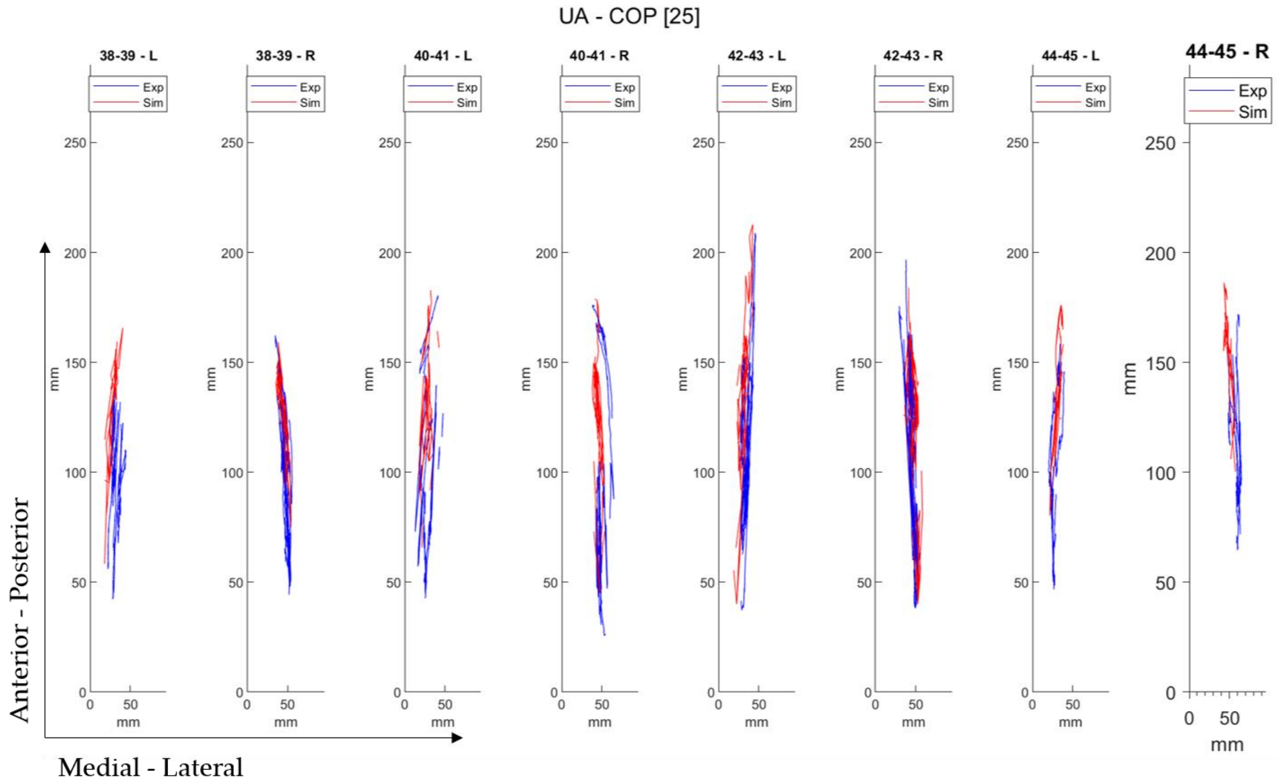

Figure 83: Anterior - Posterior and Medial - Lateral COP, calculated as in [25], Root Mean Square Error (RMSE), in percentage respectively of length insole and of width insole, of each insole size during unloaded ascending. The insoles were represented respectively: 38-39 in red, 40-41 in blue, 42-43 in green and 44-45 in purple.

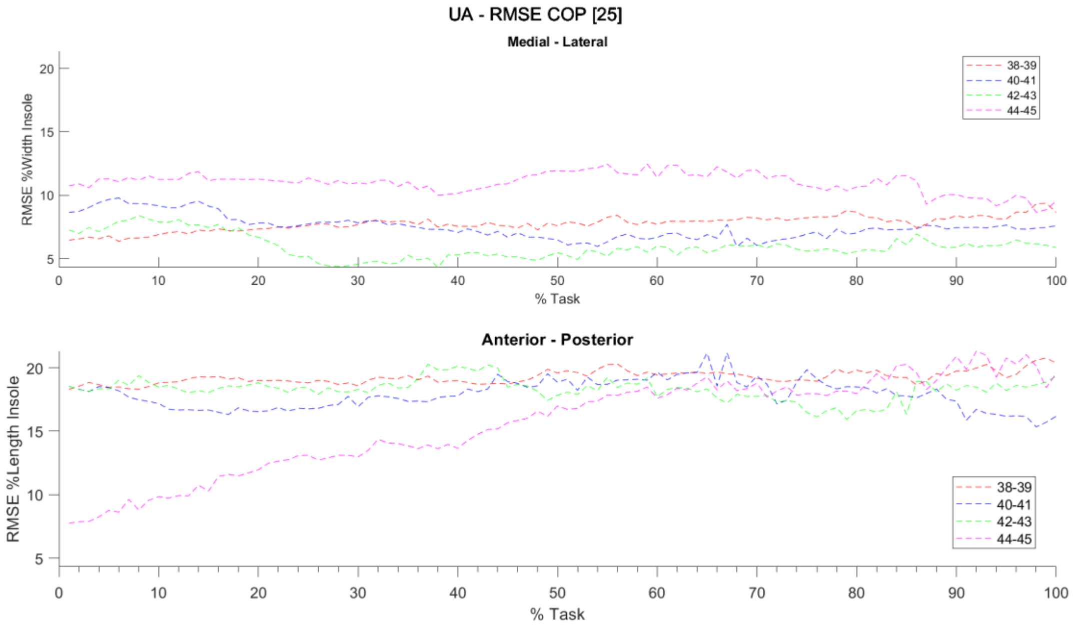

Figure S84: Medial - Lateral and Anterior - Posterior COP, calculated as in [25], Root Mean Square Error (RMSE), in percentage respectively of length insole and of width insole, of each insole size during unloaded ascending. The insoles were represented respectively: 38-39 in red, 40-41 in blue, 42-43 in green and 44-45 in purple.
